# Full genome survey and dynamics of gene expression in the greater amberjack *Seriola dumerili*

**DOI:** 10.1093/gigascience/gix108

**Published:** 2017-11-08

**Authors:** Elena Sarropoulou, Arvind Y M Sundaram, Elisavet Kaitetzidou, Georgios Kotoulas, Gregor D Gilfillan, Nikos Papandroulakis, Constantinos C Mylonas, Antonios Magoulas

**Affiliations:** Institute of Marine Biology, Biotechnology and Aquaculture Hellenic Centre for Marine Research Crete, Thalassocosmos, Gournes Pediados, P.O.Box 2214, 71003 Heraklion Crete, Greece; Norwegian High Throughput Sequencing Centre, Department of Medical Genetics, Oslo University Hospital (Ullevål), Kirkeveien 166 0450, Oslo, Norway

**Keywords:** *Seriola dumerili*; RNA-seq, genome, aquaculture, differential expression, correlation patterns, gender expression pattern

## Abstract

**Background:**

Teleosts of the genus *Seriola*, commonly known as amberjacks, are of high commercial value in international markets due to their flesh quality and worldwide distribution. The *Seriola* species of interest to Mediterranean aquaculture is the greater amberjack (*Seriola dumerili*). This species holds great potential for the aquaculture industry, but in captivity, reproduction has proved to be challenging, and observed growth dysfunction hinders their domestication. Insights into molecular mechanisms may contribute to a better understanding of traits like growth and sex, but investigations to unravel the molecular background of amberjacks have begun only recently.

**Findings:**

Illumina HiSeq sequencing generated a high-coverage greater amberjack genome sequence comprising 45 909 scaffolds. Comparative mapping to the Japanese yellowtail (*Seriola quinqueriadiata*) and to the model species medaka (*Oryzias latipes*) allowed the generation of *in silico* groups. Additional gonad transcriptome sequencing identified sex-biased transcripts, including known sex-determining and differentiation genes. Investigation of the muscle transcriptome of slow-growing individuals showed that transcripts involved in oxygen and gas transport were differentially expressed compared with fast/normal-growing individuals. On the other hand, transcripts involved in muscle functions were found to be enriched in fast/normal-growing individuals.

**Conclusion:**

The present study provides the first insights into the molecular background of male and female amberjacks and of fast- and slow-growing fish. Therefore, valuable molecular resources have been generated in the form of a first draft genome and a reference transcriptome. Sex-biased genes, which may also have roles in sex determination or differentiation, and genes that may be responsible for slow growth are suggested.

## Background Information

**Figure 1:**
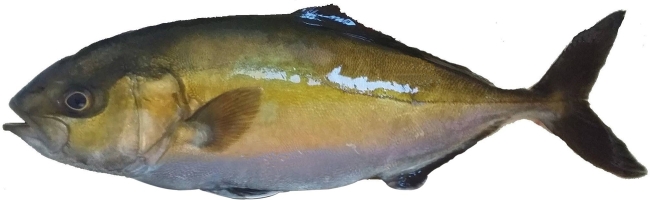
Image of the greater amberjack (*Seriola dumerilii*).

*Seriola* species, belonging to the family Carangidae and commonly known as amberjacks, are of high commercial value and have a significant international market due to their first-rate flesh quality, fast growth, and worldwide distribution. The main representatives of the family of interest to the growing aquaculture industry are the greater amberjack (*Seriola dumerili*, NCBI taxon ID: 41 447) (Fig. 1), the Japanese yellowtail (*Seriola quinqueradiata*, NCBI taxon ID:8161), the yellowtail kingfish (*Seriola lalandi*, NCBI taxon ID:302 047), and the longfin yellowtail (*Seriola rivoliana*, NCBI taxon ID: 173 321) [1, 2]*.* However, in captivity, reproductive dysfunction, as well as growth dysfunction, hinders their domestication. In addition, under captive conditions, fish may exhibit skewed sex ratios or precocious maturation before reaching market size. Teleost fishes are known to have a broad range of sex-determining mechanisms, which may differ even in closely related species, and many also show sexual dimorphism in growth. Consequently, sex control is one of the most important and highly targeted research fields in aquaculture. Concerning sex determination in the Carangidae species studied so far, no heteromorphic sex chromosome has been recorded [3]. Another important aspect in fish aquaculture is fish growth, which is a multifaceted physiological trait involving many different parameters. It can be influenced by nutrition and environment, as well as by genetic factors. Investigations to unravel the molecular background of these traits may contribute significantly to the development of reliable domestication technology.

The greater amberjack has become an attractive species for the Mediterranean industry to develop aquaculture practices due to its high growth rate. It represents the largest member of the family Carangidae [4], and it is a pelagic fish with a broad-based zoogeographical distribution and a tendency to inhabit reefs, wrecks, and artificial structures such as oil platforms [5–7]. Like other Seriola species, greater amberjack reproduction in captivity has proved to be challenging [8]. Greater amberjacks do not show obvious sexual dimorphism, but, as in a number of other teleost fish species, the ability to distinguish the sexes is an important factor for stock management and efficient fish farming. It has also been reported that growth in the greater amberjack is restricted in individuals reared in captivity. Slow-growing fish present a bottleneck in aquaculture, as small individuals have higher mortality rates, and if they were to comprise a significant number of the stock, they would contribute to inefficient farming. Insights into molecular mechanisms may lead to a better understanding of physiological traits such as growth and sex. To date, genetic resources for *Seriola* species have been developed mainly for yellowtail kingfish and the Japanese yellowtail, including genetic linkage maps [9, 10] and a radiation hybrid (RH) map [9], as well as the production of transcriptome data [11]. For the greater amberjack, very few molecular resources have been published, but they do include a cytogenetic characterization, which revealed in total 24 mainly acrocentric chromosomes (2n) and, similar to other Carangidae species, no morphologically differentiated sex chromosome [12]. The greater amberjack and the Japanese yellowtail are gonochoristic species, and phylogenetic analysis showed that they diverged 55 mya [13]. For the Japanese yellowtail, it has been shown that sex is determined by the ZZ-ZW sex-determining system, and the sex-linked locus has been localized in linkage group (LG) 12 [9, 10].

## Data Description

### Context

The present study reports for the first time gonad-specific gene expression, as well as differences between the muscle transcriptomes of slow-growing and fast/normal-growing amberjacks reared under cultured conditions. It further suggests by a comparative mapping approach a gender-specific genome region in the greater amberjack. Key molecular resources in the form of the first greater amberjack genome assembly, as well as transcriptome data for further functional studies, have therefore been generated.

### Methods

All procedures such as handling and treatment of fish used during this study were performed according to the Replacement, Reduction, Refinement (3 Rs) guiding principles for more ethical use of animals in testing, first described by Russell and Burch in 1959 (EU Directive 2010/63).

An overview of the complete workflow is given in Additional file 9.

### Sampling

Blood, sperm, and muscle sampling was performed at the aquaculture facilities of the Hellenic Centre for Marine Research (HCMR), Heraklion Crete. Blood samples obtained from adult fish were immediately placed in BD Vacutainer Plastic K3 EDTA blood collection tubes (reference number 368 857, BD, Franklin Lakes, NJ, USA). Muscle samples of slow-growing (n = 4 fish: 2 × 24 g and 2 × 20 g) and fast/normal-growing (n = 4 fish: 60 g, 94 g, 106 g, and 120 g) individuals were taken at the age of 5 months, transferred to tubes containing RNAlater (Qiagen, Hilden, Germany), and stored at –80°C until processing. Gonad samples of 4 mature female and 4 male amberjacks (n = 4 fish) were received from fish maintained at an aquaculture facility in Salamina (Argosaronikos Fishfarming S.A., Salamina, Greece) during the peak of the reproductive season (end of May/early June). At the time of sampling, fish were 4 years old and had a body size ranging between 9 and 17 kg [8]. The females were in advanced vitellogenesis, while the males were either in active spermatogenesis or contained luminal spermatozoa, with few developing spermatocysts. Gonad samples were also kept in RNAlater and stored at –80°C until processing.

### High-quality DNA extraction and genomic library preparation

Genomic DNA was extracted from 1 male and 1 female individual. High-quality female and male genomic DNA was retrieved from blood and sperm, respectively, following the protocol of the Qiagen DNeasy Blood and Tissue Kit (Hilden, Germany). Genomic DNA libraries were prepared using the TruSeq polymerase chain reaction–free library kit (San Diego, California, USA) following the manufacturer's recommendations with individual barcodes.

### RNA extraction and library preparation

Τotal RNA was extracted from all samples using the Nucleospin miRNA Kit (Macherey-Nagel GmbH & Co. KG, Duren, Germany) according to the manufacturer's instructions. In brief, gonads and muscle tissues were disrupted in liquid nitrogen using mortar and pestle, dissolved in lysis buffer, and passed through a 23-gauge (0.64-mm) needle 5 times to homogenize the mixture. RNA quantity was determined using a NanoDrop ND-1000 spectrophotometer (NanoDrop Technologies Inc, Wilmington, USA), and the quality was evaluated further by agarose (1%) gel electrophoresis as well as by capillary electrophoresis (RNA Nano Bioanalyzer chips, Bioanalyzer 2100, Agilent, Waldbronn, Germany). All RNA libraries were prepared using the TruSeq stranded total RNA library kit (Illumina, USA). RNA libraries generated from 8 different muscle samples were indexed with 8 different barcodes to be run on 1 Illumina MiSeq lane, while RNA libraries generated from female and male gonads were indexed to be run on a HiSeq2500 (Illumina, USA).

### Next-generation sequencing

The 2 genome libraries (male and female gDNA) were pooled together and paired-end (125 bp)-sequenced more than 66% of 2 lanes of HiSeq 2500 (Illumina, USA). Eight RNA-seq libraries from female and male gonads were multiplexed and also sequenced in 1 lane of a HiSeq 2500 with 125 bp of paired-end reads. RNA libraries prepared from muscle tissues were 250-bp paired-end-sequenced in 1 run of a MiSeq (Illumina, USA). Raw bcl files were analyzed and de-multiplexed using the barcodes by RTA V1.18.61.0 and bcl2fastq v1.8.4 (bcl2fastq, RRID:SCR_015058).

### Bioinformatic analysis

#### Preprocessing

Quality control of raw fastq files was assessed using the open source software FastQC, version 0.10.0 (FastQC, RRID:SCR_014583) [46]. Preprocessing of reads was performed to remove adapter contamination, followed by trimming of low-quality reads using Trimmomatic v0.33 (Trimmomatic, RRID:SCR_011848) software [47]. Reads mapping to PhiX Illumina spike-in were removed using bbmap v34.56 [48]. Reads longer than 36 nt were retained for further analyses.

#### Genome assembly

Cleaned data from male and female gDNA were concatenated and normalized to ∼50× coverage using the *in silico* read normalization tool in Trinity v2.0.6 (Trinity, RRID:SCR_013048) [49]. Resulting data were assembled using MaSuRCA v3.1.3 [50] using default parameters. The quality of the assembly was checked using BUSCO v3.0.2 (BUSCO, RRID:SCR_015008) [51], using Eukaryota_odb9 and zebrafish as lineage dataset and reference, respectively. Further analysis of the genome was performed calculating the kmer content using KmerGenie v1.6982 [51–53]. Kmer content was calculated for all trimmed data, 50× as well as 75× normalized data. GenomeScope vs 1.0 fast profiling [54] was used to assess the heterozygosity level.

#### Comparative mapping

**Figure 2:**
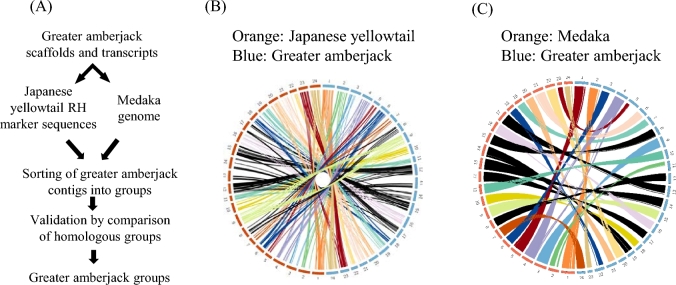
Overview of comparative mapping approach. **(a)** Workflow for the generation of *in silico* groups of the greater amberjack (*Seriola dumerilii*). **(b)** Circos illustration of mapping results between the greater amberjack (blue) and mapping results of transcripts to the Japanese yellowtail (orange)*.***(c)** Circos illustration of transcript mapping results between the greater amberjack (blue) and mapping results of transcripts to medaka (orange)*.*

Comparative mapping was applied in order to group the assembled scaffolds of the greater amberjack generated in the present study. Therefore, publicly available sequences of the Japanese yellowtail RH map [9], as well as the already established synteny of the Japanese yellowtail with medaka [17], were used as the backbone for the current comparative mapping approach. Both species contain 24 chromosomes, similar to the greater amberjack; consequently, a 1-to-1 relationship could be established. First, all available RH markers of the Japanese yellowtail were mapped using blastall 2.2.17 in the BLAST toolkit and a stringent e-value of <1E-10 to the greater amberjack reference transcriptome, as well as to the generated greater amberjack genome scaffolds. Scaffolds were grouped and named according to the linkage groups of the Japanese yellowtail. The greater amberjack reference transcriptome and the generated greater amberjack genome scaffolds were also mapped, as described above, to the medaka genome (downloaded from the Genome Browser Gateway—Oct. 2005 version 1.0 draft assembly, equivalent to the Ensembl Oct. 2005 MEDAKA1 assembly) and validated by comparing homologous groups among the greater amberjack, the Japanese yellowtail, and medaka*.* Scaffolds belonging to 1 chromosome of medaka and to the homologous group of the Japanese yellowtail were grouped together, sorted according to their match in medaka, and concatenated in order to generate *in silico* groups in the greater amberjack. The reference transcriptome was mapped to the concatenated genome scaffolds (with % identity 100% and e-value = 0), and the concatenated genome scaffolds were mapped onto the genome of medaka, three-spined stickleback, and tetraodon (*Tetraodon nigroviridis*). Syntenic groups to the Japanese yellowtail and medaka were visualized by circos v0.69–_3_ (Circos, RRID:SCR_011798) (Fig. 2b,c) [55].

#### Genome annotation and reference transcriptome assembly

Processed data from RNA samples were assembled using Trinity v2.0.6. Initially, the data were normalized to 50× coverage and then assembled using default parameters (–SS_lib_type RF). The relative abundance of each transcript/isoform was calculated using RSEM, and transcripts with low coverage were filtered using the filter_fasta_by_rsem_values.pl tool with the following parameters: tpm_cutoff 1, fpkm_cutoff 0, and isopct_cutoff 1. Two-pass iterative MAKER v2.31.8 (MAKER, RRID:SCR_005309) [56] was used to predict genes from the generated genome assembly using the Trinity assembled transcriptome as EST evidence and the UniProt Sprot protein database (UniProt, RRID:SCR_002380) as protein homology evidence. HMM files created using SNAP v2006–07-28 [57] and GeneMark-ES Suite v4.21 [58] were used on the first pass for gene prediction, and the Augustus v3.0.1 (Augustus: Gene Prediction, RRID:SCR_008417) gene prediction species model based was used during the second pass to refine gene prediction.

Predicted protein sequences were annotated using blastp in the BLAST v2.2.29 toolkit against the NCBI nr database and using InterproScan against Interpro protein domains. Blast2GO v3.3.5 (Blast2GO, RRID:SCR_005828) was used to merge the 2 results, and gene ontology (GO) mapping was performed using the same software. This reference transcriptome was used for differential expression analyses.

#### Differential expression analysis

Processed reads from 4 testis and 4 ovary samples were aligned against the assembled genome and predicted transcriptome using Tophat2 v2.0.13 (TopHat, RRID:SCR_013035), and reads mapping to genes (MAKER2 gtf) were counted using featureCounts v1.4.6-p1. Differential expression was calculated using DESeq2 v1.10.1 [59] in R v3.2.4 [60] with default methods implemented in the function “DESeq” within this tool as the data is assumed to fit the negative binomial generalized linear model. A similar pipeline was used to calculate differential expression between fast/normal- and slow-growing individuals.

#### Data evaluation

Samples were clustered in order to detect possible outliers applying the WCGNA software package [61], which detects possible outliers based on their Euclidean distance (Additional files 1 and 4). For further data evaluation, the biological replicates were validated by calculating the sample-to-sample distances, illustrated in the form of a heatmap between the samples using the free available scripts within the DeSeq2 package. The heatmap of the distance matrix gives an overview of similarities and dissimilarities between the samples. Apart from clustering using Euclidean distance, principal component (PCA) 2D plot analysis was performed to show the overall effect of experimental covariates, as well as batch effects [62]. Finally, hierarchical clustering of significantly differentially expressed transcripts was performed to illustrate the up- and downregulated transcripts.

#### Meta-analysis

Differentially expressed transcripts between male and female gonads, as well as between slow- and fast/normal-growing individuals were annotated using BLAST search (version 2.2.25) [63] against the nonredundant protein database and nonredundant nucleotide database. Blast2GO (Blast2GO, RRID:SCR_005828) software [64] was applied to determine GO terms (cellular component, molecular function, and biological process), as well as to perform enrichment analysis. Enrichment analysis was carried out using all assembled transcripts as the reference set, and the differentially expressed genes (male vs female gonads and slow- vs fast/normal-growing individuals), as well as transcripts mapped to the *in silico*–generated groups as the test set. Default parameters were chosen, i.e., 2-tailed test and a false discovery rate <0.05.

## Data Validation and Quality Control

### Genome sequencing, assembly, and annotation

Whole-genome sequencing was performed on genomic DNA from 1 female and 1 male specimen of the greater amberjack, generating 345 544 307 150 bp of paired-end reads. After data preprocessing and *in silico* normalization, 230 856 386 reads were obtained, which were further used to assemble the draft genome. Assembly of the genome produced a 669 638 422-bp (∼670-Mb) genome represented in 45 909 scaffolds made up of 62 353 contigs. The longest scaffold was 575 738 bp long, with an N50 scaffold length of 75.1 kb and N50 contig length of 36.6 kb. Kmer profiling (Additional file 1) showed that 50× normalized data had the same kmer distribution as the original sequenced (all) data. Kmer distribution for 75× genome coverage did not resemble the original dataset. KmerGenie recommended the best kmer for 50× and all data as 89 and 79, respectively. MaSuRCA independently calculated a kmer profile and used 85 as the kmer value while assembling the 50× normalized data. Further genome analysis applying GenomeScope revealed a low heterozygosity level (0.649%). Maker2 analyses predicted 108 524 genes and 116 045 transcripts in the genome, which after applying the recommended threshold [16] of annotation edit distance <1, resulted in 45 547 and 53 023 high-quality genes and transcripts, respectively. Out of 53 023 transcripts, 33.6% were successfully annotated using BLAST against the NCBI nonredundant database with an e-value <10^−5^. BUSCO was used to evaluate the assembled genome and the transcriptome, and out of 303 BUSCO groups (i.e., conserved orthologs) specific to eukaryotes, more than 93% were identified to be encoded by both the genome and the transcriptome assembly (Table 1).

**Table 1:** BUSCO results

	Genome		Trancriptome	
Complete BUSCOs	284	93.7%	283	93.4%
Complete and single-copy BUSCOs	270	89.1%	219	72.3%
Complete and duplicated BUSCOs	14	4.6%	64	21.1%
Fragmented BUSCOs	8	2.6%	13	4.3%
Missing BUSCOs	11	3.6%	7	2.3%
Total BUSCO groups searched	303		303	

### Comparative mapping

**Figure 3:**
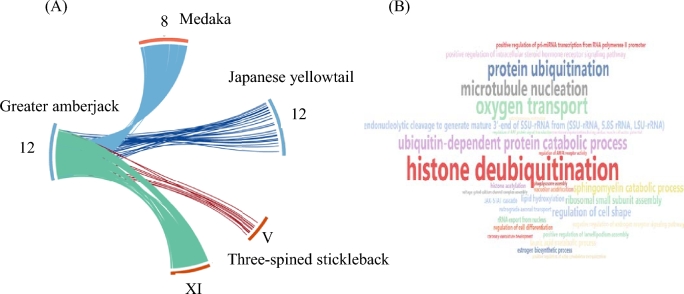
Identification by synteny of a putative amberjack sex determination *in silico* linkage group. **(a)** Putative sex-linked group, *in silico* group 12 of the greater amberjack, compared with medaka, Japanese yellowtail, and three-spined stickleback. **(b)** Word cloud illustration of transcripts mapped to SD12 as test set and all identified transcripts as the reference set.

Using a comparative mapping approach (Fig. 2a), 468 Japanese yellowtail molecular markers retrieved from the publicly available Japanese yellowtail RH map were successfully mapped to 409 greater amberjack scaffolds (Table 2), while 14 990 greater amberjack scaffolds were successful mapped to the 24 chromosomes of medaka. This enabled the generation of *in silico* groups and subsequent synteny analysis. *In silico*–generated groups of the greater amberjack were named according to the RH groups of the Japanese yellowtail (Fig. 2b) [9, 17]. Out of the 53 023 obtained transcripts, 44 371 (∼84%) were successfully mapped to the generated *in silico* groups of the greater amberjack, and 30 342 transcripts (∼57%) were mapped to the genome of medaka (Fig. 2c, Table 2). In the Japanese yellowtail, LG12 has been identified as the putative sex-determining linkage group [15]. Transcripts mapping to the greater amberjack *in silico* group 12 mapped successfully to their homologous group of the Japanese yellowtail (LG12), medaka (chr. 8), and three-spined stickleback (chr.V and chr. XI) (Fig. 3a). Analysis of transcripts successfully mapped to the *in silico* group 12 (Additional file 2) revealed an enrichment for those involved in ubiquitination and de-ubiquitination (Fig. 3b, Additional file 3).

**Table 2:** Comparative mapping of greater amberjack scaffolds and transcripts to the Japanese yellowtail RH map and to the medaka genome

Japanese yellowtail RH group	Number of Japanese yellowtail markers mapped to greater amberjack scaffolds	Homologous medaka chromosomes	Number of greater amberjack transcripts mapped to the medaka genome
SQ1	30	OL5	1451
SQ2	19	OL1	1425
SQ3	12	OL6	1423
SQ4	19	OL4	1490
SQ5	14	OL23	777
SQ6	26	OL21	1197
SQ7	13	OL19	1046
SQ8	16	OL15	1204
SQ9	25	OL3	1307
SQ10	17	OL11	1269
SQ11	12	OL2	689
SQ12	36	OL8	1575
SQ13	5	OL17	1642
SQ14	12	OL13	1378
SQ15	32	OL9	1534
SQ16	18	OL16	1514
SQ17	17	OL12	1233
SQ18	16	OL10	1095
SQ19	24	OL14	1318
SQ20	16	OL22	1350
SQ21	17	OL20	993
SQ22	17	OL18	816
SQ23	23	OL24	1139
SQ24	31	OL7	1477

OL: *Oryzias latipes*; SQ: *Seriola quinqueradiata*.

### Gender-specific gene expression profiles

**Figure 4:**
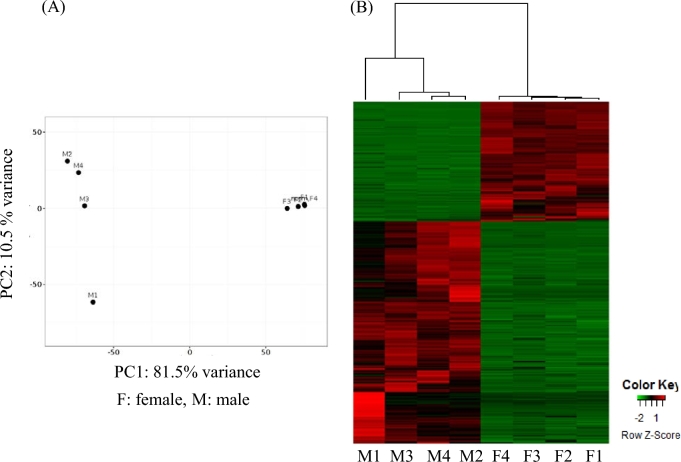
Overview of transcriptome study of female vs male gonads. **(a)** PCA plot of transcripts significantly differentially expressed. **(b)** Heatmap of transcripts significantly (*P*adj < 0.005 and log_2_ FC > |2|) differentially expressed. Green color represents upregulated transcripts in male gonads while red color signifies upregulated transcripts in female gonads.

**Figure 5:**
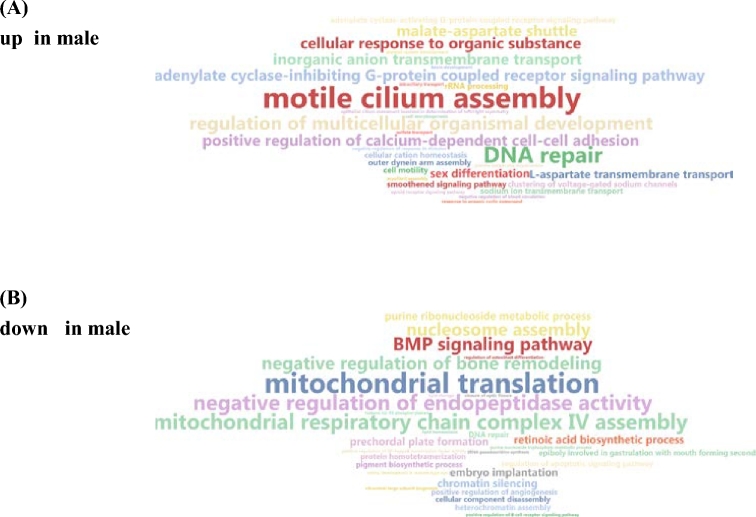
Word cloud illustration of significantly enriched GO terms of the category Biological Process. **(a)** Enriched GO terms of transcripts upregulated in male gonads. **(b)** Enriched GO terms of transcripts downregulated in male gonads.

The gonadal transcriptomes of 4 female and 4 male individuals sampled during the reproductive season were sequenced on the Illumina HiSeq platform, resulting in a total of 78 264 170 and 57 561 139 raw reads, respectively. After trimming and PhiX removal, approximately 80% of the reads remained, of which 70% aligned to the generated genome using tophat2 (Table 3). Cluster analysis demonstrated a clear division of female and male gonad expression between the 2 sample groups (Additional file 4). Significantly differentially expressed transcripts (*P*adj < 0.005 and log_2_FC > |2|) between genders amounted to 7199 transcripts, with 2522 being more highly expressed in female gonads and 4677 in male gonads (Fig. 4b, Additional file 5). Principal component clustering (Fig. 4a), as well as hierarchical clustering (Fig. 4b), illustrated in the form of a heatmap, clearly showed again the separation of female and male gonad gene expression patterns. In addition, the latter revealed that the majority of transcripts had higher expression in male gonads in comparison with the female gonads. A total of 4266 of the significant differentially expressed transcripts were successfully assigned to 1 of the generated greater amberjack *in silico* groups (Additional file 6). Enrichment analysis of transcripts more highly expressed in the male gonads resulted in GO terms involved in regulation but also in sex differentiation (Fig. 5a), while transcripts more highly expressed in the female gonads resulted in GO terms including mitochondrial translation and mitochondrial respiratory chain complex IV assembly (Fig. 5b).

**Table 3:** RNA sequencing reads derived from 4 female gonads and 4 male gonads by Illumina HiSeq sequencing

	Raw reads	Trimmed reads	After PhiX removal	% of raw reads used downstream analyses	Tophat2 align	
F1	17 268 356	14 425 361	14 391 074	83.34%	10 210 127	70.9%
F2	17 083 394	13 542 326	13 516 020	79.12%	9 601 965	71.0%
F3	23 331 846	19 330 707	19 265 030	82.57%	13 835 238	71.8%
F4	20 580 574	16 881 832	16 834 901	81.80%	12 066 021	71.7%
M1	13 860 606	11 838 653	11 805 696	85.17%	8 603 874	72.9%
M2	15 315 961	12 898 261	12 868 488	84.02%	9 205 543	71.5%
M3	15 086 732	12 036 225	11 997 908	79.53%	8 623 374	71.9%
M4	13 297 840	10 635 465	10 600 787	79.72%	7 548 436	71.2%

F: female gonads; M: male gonads

### Expression profiles of slow- vs fast/normal-growing individuals

**Figure 6:**
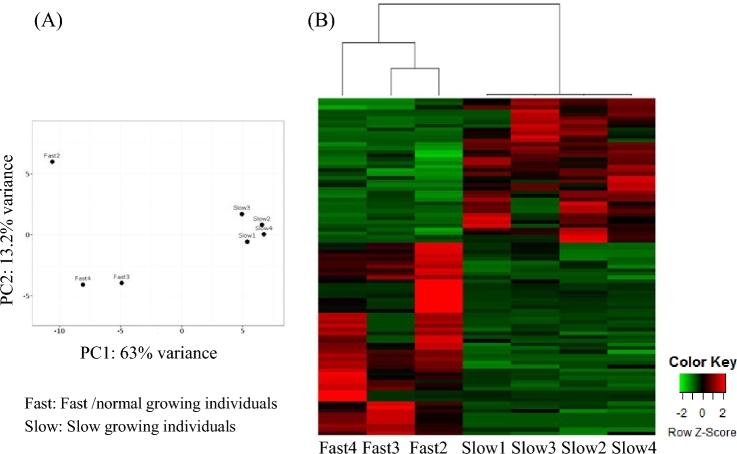
Overview of transcriptome study of slow-growing vs. fast/normal-growing amberjacks. **(a)** PCA plot of transcripts significantly differentially expressed. **(b)** Heatmap of transcripts significantly differentially expressed. Green color represents upregulated transcripts in fast/normal-growing individuals while red color signifies upregulated transcripts in slow-growing individuals.

**Figure 7:**
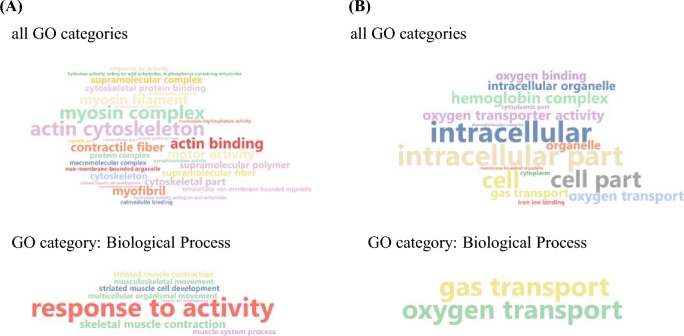
Word cloud illustration of significantly enriched GO terms. **(a)** Enriched GO terms of transcripts upregulated in fast/normal-growing individuals vs slow-growing individuals. **(b)** Enriched GO terms of transcripts upregulated in slow-growing individuals vs fast/normal-growing individuals.

The muscle transcriptomes of 4 slow- and 4 fast*/*normal-growing individuals were sequenced on the Illumina MiSeq platform, resulting in a total of 10 625 681 and 9 005 157 raw reads, respectively. After preprocessing, approximately 85% of the reads remained, of which about 50% aligned to the generated genome using tophat2 (Table 4). Outlier detection analysis led to the exclusion of 1 fast/normal grower from the downstream analysis (Additional file 7). Transcripts with *P*-values lower than 0.005 and more than a log_2_ FC were considered differentially expressed, resulting in 40 transcripts being upregulated in slow-growing individuals, and 52 transcripts being upregulated in fast/normal-growing individuals (Fig. 6). Enrichment analysis showed that transcripts upregulated in fast/normal-growing individuals comprise GO terms related to muscle physiology (Fig. 7a). On the other hand, transcripts found to be upregulated in slow-growing individuals were mainly found within the GO biological process terms “gas transport” and “oxygen transport” (Fig. 7b).

**Table 4:** RNA sequencing reads derived from muscle tissue of fast/normal- and slow-growing individuals by Illumina MiSeq sequencing

	Raw reads	Trimmed reads	After PhiX removal	% of raw reads used for downstream analyses	Tophat2 align	
Fast1	2 434 221	2 164 923	2 148 193	88.25%	914 312	42.56%
Fast2	2 335 103	2 004 168	1 990 068	85.22%	921 965	46.33%
Fast3	2 944 122	2 649 012	2 627 657	89.25%	1 265 753	48.17%
Fas4	2 912 235	2 616 056	2 600 521	89.30%	1 304 009	50.14%
Slow1	2 088 813	1 843 495	1 830 095	87.61%	868 946	47.48%
Slow2	2 263 688	1 941 004	1 925 396	85.06%	985 902	51.21%
Slow3	2 497 198	2 197 131	2 178 922	87.25%	958 565	43.99%
Slow4	2 155 458	1 892 706	1 877 917	87.12%	869 033	46.28%

## Re-use Potential and Discussion

The rapid growth and large size of greater amberjack, as well as its high-quality flesh and worldwide distribution, have drawn the attention of the aquaculture sector. The development of appropriate and efficient husbandry practices for industrial production has, however, proved difficult. Insights into its molecular background may enhance the prospects of discovering important aquaculture-related traits, and consequently contribute to the more rapid development of appropriate husbandry practices. The present study includes a draft genome assembly of the greater amberjack of approximately 670 Mb, generated from 345 544 307 paired-end reads obtained by Illumina sequencing. The genome size of the greater amberjack has been estimated to be 0.74 pg [18]. Hence, it is anticipated that its genome has been sequenced to approximately 75× coverage in this study. Similar genome sizes have been reported for 2 other aquaculture species important in the Mediterranean, the gilthead sea bream (*Sparus aurata*) [19] and the European sea bass (*Dicentrarchus labrax*) [20]. While the genome of the gilthead sea bream has still not been published, the genome of the European sea bass (v1.0c) has been sequenced to approximately 30× coverage by Sanger, 454, and Illumina sequencing [21]. The draft assembly of the European sea bass genome and the draft assembly of the greater amberjack produced similar N50 contig lengths (54 kb and 37 kb, respectively), but differed significantly in N50 scaffold length. For the European sea bass genome, an N50 scaffold length of 4.9 Mb has been reported, while the N50 scaffold length for the greater amberjack described here is only 75 kb. This result is not surprising, as here only Illumina paired-end sequencing has been performed. To increase scaffold length, the addition of longer reads from a second technology would be necessary. Nonetheless, the obtained N50 scaffold length here compares favorably with those of other teleost species (i.e., *Chatrabus melanurus, Chaenocephalus aceratus*, and *Bregmaceros cantori*), which have been sequenced to a similar depth with only Illumina paired-end data and generated N50 scaffold lengths as low as 7 kb [22].

Like the chromosome number in the Japanese yellowtail, previous cytogenetic analysis determined the haploid number of chromosomes in the greater amberjack to be 24 [12]. Applying a comparative mapping approach based on previously published genetic linkage and RH maps of Japanese yellowtail [13, 14, 22] as well as the medaka genome, the generated greater amberjack scaffolds were clustered successfully to 24 *in silico* groups (Table 2, Fig. 2b). Subsequent mapping of the generated greater amberjack transcripts to the medaka genome and to the generated greater amberjack *in silico* groups resulted in a one-to-one relationship (Fig. 2c).

Comparative mapping allows the identification of markers for traits of interest either based on candidate genes or based on previous QTL studies in the same or other species. In this way, a sex-linked locus was found in LG12 of the Japanese yellowtail [15]. Transcripts mapped to the greater amberjack *in silico* group 12 mapped to medaka Chr.8 and three-spined stickleback Chr.V and XI (Fig. 3a, Additional file 8), although in neither case have these been reported as sex-determining chromosomes [23, 24]. In the Japanese yellowtail, the sex-linked locus was without doubt linked to LG12, but despite this and the generation of a second, increased-resolution genetic linkage map [14], the sex-determining genes known to date have not been identified in the sex-determining region of the Japanese yellowtail. The authors speculated that the PDZ domain containing GIPC1 protein, found in the SD region of LG12, may be of importance in determining sex in the Japanese yellowtail. This protein was also found in the greater amberjack *in silico* group 12, but without being differentially expressed between the female and the male gonads (Additional file 2). The greater amberjack is a gonochoristic species, without any external sexual dimorphism, and genetically differentiated sex chromosomes have not yet been identified [12]. In teleost fishes, a broad range of sex-determining mechanisms have been documented, and different sex-determining genes have been reported (for a review, see [25]). The main sex-determining genes known in other teleosts were found to be located in the greater amberjack *in silico* group 1 (amhr2), group 4 (amhY), group 7 (dmY/dmrt1a), group 17 (gsdf), and group 18 (sox3Y) (Table 5). Enrichment analysis of transcripts successfully mapped in the present study to the homologous *in silico* group 12 of the greater amberjack resulted mainly in biological process GO terms related to ubiquitination (Fig. 3b). A recent and growing body of evidence points to the important role of ubiquitination in the regulation of spermatogenesis from the very beginning up to spermatid differentiation [26–28]. On the other hand, transcripts involved in ubiquination have been reported in the ovary transcriptome of the striped bass (*Morone saxtilis*) [29, 30]. Analogous enrichment analysis of the remaining greater amberjack *in silico* groups did not reveal any GO terms specific to sex regulation (Additional file 3). Τhe present findings may indicate the importance of ubiquitination during sex determination.

**Table 5:** Overview of known sex-determining regions in Teleost species identified in the greater amberjack

Gene	Abbreviation	Teleost accession number	Transcript in the greater amberjack	*In silico* group	DE* male vs female gonads	Sex determining in
DM domain gene on the Y chromosome/ doublesex and mab-3-related transcription factor 1a	*dmY/dmrt1a*	*Oryzias latipes* NM_0 011 04680/ XM_004086451 unplaced scaffold	maker-jcf7180000931253-snap-gene-0.40-mRNA-1	SD7	Up in male gonads	*Oryzias latipes* [23, 65]
Gonadal soma-derived factor	*gsdf*	*Oryzias latipes* NM_0 011 77742 chr.12	Augustus-masked-jcf7180000916295-processed-gene-0.7-mRNA-1	SD17	Up in male gonads	*Oryzias* l*uzonensis* [66]
Y chromosome–specific antimuellerian hormone	*amhY*	*Oryzias latipes* NM_0 011 04728 chr. 4	maker-jcf7180000931145-snap-gene-0.73-mRNA-2	SD4	Up in male gonads	*Odontesthes hatcheri* [67] *HM153803.1*
Antimuellerian hormone receptor 2	*amhr2*	*Lates calcarifer* KR492510 *Oryzias latipes* DQ499644.1 chr. 5 or 7	maker-jcf7180000889430-snap-gene-0.77-mRNA-5	SD1	No expression	*Takifugu genus* [68]
Sexually dimorphic o the Y chromosome	*sdY*	*Salmonidae* family	n/a	n/a	n/a	*Salmonidae family* [69]
SRY-box containing protein 3Y	*sox3Y*	Oryzias latipes AJ245396 chr.10	augustus-masked-jcf7180000920392-processed-gene-0.31-mRNA-1	SD18	Up in female gonads	*Oryzias dancena* [65]

DE: differential expression; SD: *Seriola dumerili.*

* Greater amberjack data.

In addition to genome sequencing, the gender-specific mechanisms at the transcriptome level operating in the greater amberjack were investigated. It has to be noted that a link between sex determination and sex-specific expression is neither necessary nor expected. At this point, gonad-specific transcripts were identified, with more transcripts found to be highly expressed in male than in female gonads (Fig. 4a and b, Additional file 5).

**Figure 8:**
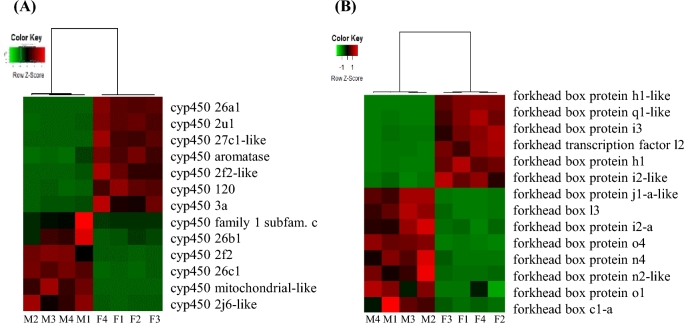
Gene expression displayed as heatmaps of 2 gene families comprising significantly differentially expressed genes in female and male gonads. **(a)** Cytochrome P450 family. **(b)** Forkhead box protein family.

Among the female gonads’ biased transcripts, 12 transcripts were identified belonging to the zona pellucida (zp) proteins (Additional file 5). It has been shown that during oocyte development, the oocyte is surrounded by an acellular envelope comprising zp proteins [31–34]. Also in other transcriptomic studies in fish, it has been reported that zp proteins are more highly expressed in female gonads (e.g., [33]). Another well-known gene family identified as being involved in sex differentiation is the cathepsin gene family. Cathepsins are responsible for the degradation of vitellogenin into yolk proteins [36]. In the present study, 7 transcripts were identified as being differentially expressed, with 5 of them being more highly expressed in female gonads (Additional file 5). Cathepsin S and z-like showed the highest log_2_ FC (∼10). Cathepsin S has also been reported to be more highly expressed in the female olive flounder (*Paralichthys olivaceus*) [35]. Furthermore, the well-documented ovary marker for teleost species cytochrome P450 aromatase gene, cyp19a [37], was also identified in the present study as being more highly expressed in the female gonads. Enzymes encoded by cyp450 genes play an important role in the synthesis and metabolisms of steroid hormones, as well as of certain fats and acids used to digest fats. The differentially expressed transcripts encoding for cyp450 genes in the present study comprised 7 cyp450 transcripts more highly expressed in female gonads and 6 more highly expressed in male gonads (Fig. 8a). Interestingly, cyp4502f2 was more highly expressed in male gonads while cyp450 2f2-like protein was more highly expressed in female gonads. Cyp4502f2 belongs to the gene family encoding monooxygenase activity that is important for detoxification. To date, cyp4502f2 has been found mainly to be expressed in the liver and lung [38], while its expression in gonads has not yet been reported. It is also known that cyp450 genes are involved in the retinoid acid (RA) pathway, which is important in ovarian differentiation. Among the cyp450 genes, cyp26 enzymes contribute to the regulation of RA levels. Interestingly, cyp26 has 2 paralogous genes, cyp26a1 and cyp26b1, which were found with opposite gender expression patterns in the greater amberjack (Fig. 8). This has also been shown in the hermaphrodite species bluehead wrasse (*Thalassoma bifasciatum*) [39], but also in Nile tilapia (*Oreochromis niloticus*) [40] and mice (*Mus musculus*) [41].

Forkhead box protein L2 (*foxl2*) has also been shown to have an important role in female sex differentiation [42]. Forkhead box proteins are transcription factors with significant regulatory roles during development and cell growth proliferation and differentiation. To our best knowledge, among foxl proteins, foxl2 has been reported as being involved during ovarian differentiation [35], and foxl3 has been detected as having gender-biased expression in fish [39]. In the present work, a total of 14 transcripts encoding for forkhead box proteins were identified as having gender-specific expression patterns, including foxl2 being more highly expressed in female gonads and foxl3 being more highly expressed in male gonads (Fig. 8b). Interestingly, in mice it has been speculated that elevated cyp26b1 levels uphold the male fate of germ cells in testes and that foxl2 antagonizes cyp26b1 expression in ovaries [43]. Both genes also showed expression in the present study consistent with this, indicating that the hypothesis that the RA signaling pathway may play a significant role in gonadal sex change regulation in hermaphroditic fish [39] may also be true for gonochoristic species.

In addition, the present study is the first to attempt to assess the molecular background of slow-growing vs fast/normal-growing greater amberjacks. White muscle was selected to investigate differential expression analysis, as it comprises the majority of the myotome and consequently is expected to isolate mainly transcripts encoding for structural proteins involved in myogenesis and growth [44]. In the present study, transcripts mainly involved in processes affecting muscle physiology were found to be enriched in fast/normal-growing individuals (Fig. 7). On the other hand, enrichment analysis of transcripts significantly upregulated in slow-growing individuals revealed that these mainly activated the processes of gas and oxygen transport (Fig. 7b). Similar results were also reported in a recent study of slow- vs fast-growing rainbow trout (*Oncorhynchus mykiss*) [45]. In comparison with the present study, slow-growing rainbow trout showed elevated mitochondrial and cytosolic creatine kinase expression levels whereas fast-growing fish revealed an elevated cytoskeletal gene component expression level. Growth is in general a multifaceted process and comprises many interacting factors. The fact that the present study identified a clear expression pattern between the 2 groups by applying a medium throughput Illumina platform points to the noteworthy possibility of applying low-depth RNA-seq, available to a number of small laboratories, in order to gain first insights into important physiological processes.

## Conclusion

The present study has provided the first insights to the molecular background of male and female individuals, as well as of fast- and slow-growing fish, of the greater amberjack. By this means, the genome of an important new aquaculture fish species was reported, as well as the gonad and muscle transcriptomes. Illumina HiSeq sequencing generated a high-coverage genome sequence comprising 45 909 scaffolds. Comparative mapping to the Japanese yellowtail, as well as to the model fish species medaka, allowed the generation of *in silico* groups comprising 83% of the obtained transcripts. Transcripts found to be more highly expressed in male and female gonads were identified, and comprised known sex-determining and sex-differentiation genes. Further differential expression analysis of fast/normal- vs slow-growing amberjacks points to an important role of oxygen and gas transport in relation to slow-growing individuals, whereas in fast/normal-growing fish important transcripts involved in muscle function are significantly upregulated.

## Availability of data and materials

Datasets supporting the results of this article are available in the *Giga*DB (*Giga*DB, RRID:SCR_004002) repository associated with this publication [70]. All datasets were submitted to the public databases of the International Nucleotide Sequence Database Collaboration (INSDC), provided by DDBJ, EMBL-EBI, and NCBI. All data and metadata were submitted under Bioproject number PRJNA384295. Raw data are available from the SRA database under accession number SRP105319.

## Additional files

Additional file 1: (a) Kmer profile plot using the GenomeScope fast reference-free genome profiling method showing the fit of the model to the observed kmer frequency. The shape of the kmer profile reflects the complexity of the genome. A homozygote repeat-free genome results in a kmer profile with a Poisson distribution. A 2-peak profile indicated a heterozygous genome. (b) Kmer profile plot: all data (red), 75× (grey), and 50× normalized data (black). KmerGenie recommended the best kmer for 50× and all data as 89 and 79, respectively. MaSuRCA calculated its own kmer profile and used 85 as the kmer value while assembling the 50× normalized data. The plot was generated with the haploid model in KmerGenie; 50× and 75× normalization was performed using the Trinity normalization tool. Dotted lines represent the kmer calculated for all, 75× and 50× data. The dashed line represents the kmer predicted and used by MaSuRCA.

Additional file 2: Annotated transcripts mapped onto the *in silico* group 12 of greater amberjack along with their expression values, i.e., fold changes of transcripts significantly more highly expressed in female gonads and in male gonads (XLSX 32 kb).

Additional file 3: Illustration in the form of a word cloud of enrichment analysis of transcripts successfully mapped onto the *in silico*–generated greater amberjack groups (DOCX 1093 kb).

Additional file 4: (a) Sample clustering for outlier detection resulting from RNA sequencing of female and male gonads. (b) Sample-to-sample distances. Heatmap generated with DeSeq2 software packages showing the Euclidean distances between the samples (PPTX 55 kb).

Additional file 5: Count file of individual data values showing transcripts significantly more highly expressed in female gonads and in male gonads with DEG threshold *P*adj < 0.005, |log2FC| >2, along with their putative annotations (XLSX 904 kb).

Additional file 6: Transcripts significantly more highly expressed in female gonads and in male gonads, along with their fold change value, as well as their position within the generated *in silico* groups of greater amberjack (XLSX 229 kb).

Additional file 7: (a) Fish weight of slow- and fast/normal-growing individuals. (b) Sample clustering for outlier detection resulting from RNA sequencing of fast/normal- vs slow-growing individuals (PPTX 65 kb).

Additional file 8: Illustration of comparative mapping approach of Japanese yellowtail with medaka and three-spined stickleback, respectively (PPTX 1006 kb).

Additional file 9: Workflow overview (PPTX 87 kb).

## Abbreviations

BLAST: basic local alignment search tool; bp: base pairs; chr.: chromosome; DE: differential expression; dhp: days post hatched; FC: fold change; GO: gene ontology; LG: linkage group; mya: million years ago; Mb: mega base; kb: kilo base; NCBI: National Centre for Biotechnology Information; nr: nonredundant; pg: pictograms; RH: radiation hybrid.

## Funding

This project has received funding from the Greek Ministry of Education in the frame of the National Strategic Reference Framework 2007–2013 Program (Project Marine Biology, Biotechnology and AquaCulture, Development Proposals from Research Institutions—KRIPIS) as well as from the European Union Horizon 2020 Research and Innovation Program European Marine Biological Research Infrastructure Cluster (EMBRIC) under grant agreement No. 654008.

## Author contributions

E.S. participated in designing the study, performed next-generation sequencing meta-analysis and comparative mapping analysis, and conceived and wrote the main manuscript text. A.Y.M.S. carried out the transcriptome and genome assembly and generated the differential expression matrices. E.K. performed RNA extraction, RNA library preparation, and MiSeq sequencing. G.D.G. performed genome library preparation and Illumina sequencing, N.P. contributed to the writing and interpretation of the data and carried out muscle sampling of slow- and fast/normal-growing fish. C.C M. contributed to writing and the interpretation of the data and conceived gonad and blood sampling. G.K. participated in designing the study and contributed to writing and the interpretation of the data. A.M. coordinated and designed the study and contributed to writing. All authors reviewed and approved the manuscript.

## Supplementary Material

GIGA-D-17-00141_Original-Submission.pdf

GIGA-D-17-00141_Revision-1.pdf

GIGA-D-17-00141_Revision-2.pdf

GIGA-D-17-00141_Revision-3.pdf

Response-to-Reviewer-Comments_Original-Submission.pdf

Response-to-Reviewer-Comments_Revision-1.pdf

Response-to-Reviewer-Comments_Revision-2.pdf

Reviewer-1-Report-(Original-Submission).pdf

Reviewer-1-Report-(Revision-1).pdf

Reviewer-2-Report-(Original-Submission).pdf

Reviewer-3-Report-(Original-Submission).pdf

Reviewer-3-Report-(Revision-1).pdf

Additional Files
