# Supplementary material for: Full genome survey and dynamics of gene expression in the greater amberjack Seriola dumerili
Source: Gigascience. 2017 Nov 8;6(12):1–13. doi: 10.1093/gigascience/gix108 (PMC5751066; doi:10.1093/gigascience/gix108)
Supplement: GIGA-D-17-00141_Revision-3.pdf [file gix108_giga-d-17-00141_revision-3.pdf]

GigaScience

Full Genome Survey and Dynamics of Gene Expression in the Greater Amberjack Seriola dumerili

--Manuscript Draft--

**Manuscript Number:**	GIGA-D-17-00141R3
**Full Title:**	Full Genome Survey and Dynamics of Gene Expression in the Greater Amberjack Seriola dumerili
**Article Type:**	Data Note
**Funding Information:**	Greek Ministry of Education, NSRF 2007-2013 Program (Project MBBC, Development Proposals from Research Institutions - KRIPIS) — Not applicable<br>European Unions Horizon 2020 Research and Innovation Program European Marine Biological Research Infrastructure Cluster (EMBRIC) (No. 654008) — Not applicable
**Abstract:**	Background:<br>Teleosts of the genus Seriola, commonly known as amberjacks, are of high commercial value in international markets due to their flesh quality and worldwide distribution. The Seriola species of interest to Mediterranean aquaculture is the greater amberjack (Seriola dumerili). This species holds great potential for the aquaculture industry, but in captivity, reproduction has proved to be challenging and observed growth dysfunction hinders their domestication. Insights into molecular mechanisms may contribute to a better understanding of traits like growth and sex, but investigations to unravel the molecular background of amberjacks have begun only recently.<br>Results:<br>Illumina HiSeq sequencing generated a high coverage greater amberjack genome sequence comprising 45,909 scaffolds. Comparative mapping to the Japanese yellowtail (Seriola quinqueriadiata) and to the model species medaka (Oryzias latipes) allowed the generation of in silico groups. Additional gonad transcriptome sequencing identified sex-biased transcripts, including known sex-determining and differentiation genes. Investigation of the muscle transcriptome of slow-growing individuals showed that transcripts involved in oxygen and gas transport were differentially expressed compared to fast/normal-growing individuals. On the other hand, transcripts involved in muscle functions were found to be enriched in fast/normal-growing individuals.<br>Conclusion:<br>The present study provides first insights into the molecular background of male and female amberjacks and of fast and slow-growing fish. Therefore, valuable molecular resources have been generated in the form of a first draft genome and a reference transcriptome. Sex-biased genes which may also have roles in sex determination or differentiation, and genes that may be responsible for slow growth are suggested.
**Corresponding Author:**	Elena Sarropoulou<br><br>GREECE
**Corresponding Author Secondary Information:**	
**Corresponding Author's Institution:**	
**Corresponding Author's Secondary Institution:**	
**First Author:**	Elena Sarropoulou
**First Author Secondary Information:**	
**Order of Authors:**	Elena Sarropoulou<br>Arvind Y.M. Sundaram

	Elisavet Kaitetzidou
	Georgios Kotoulas
	Gregor D. Gilfillan
	Nikos Papandroulakis
	Constantinos C Mylonas
	Antonios Magoulas
**Order of Authors Secondary Information:**	
**Response to Reviewers:**	All comments of the reviewer are addressed and the manuscript has been transformed to the Data note format as requested by the editor following the instruction at https://academic.oup.com/gigascience/pages/data_note
**Additional Information:**	
**Question**	**Response**
Are you submitting this manuscript to a special series or article collection?	No
**Experimental design and statistics**<br><br>Full details of the experimental design and statistical methods used should be given in the Methods section, as detailed in our Minimum Standards Reporting Checklist. Information essential to interpreting the data presented should be made available in the figure legends.<br><br>Have you included all the information requested in your manuscript?	Yes
**Resources**<br><br>A description of all resources used, including antibodies, cell lines, animals and software tools, with enough information to allow them to be uniquely identified, should be included in the Methods section. Authors are strongly encouraged to cite Research Resource Identifiers (RRIDs) for antibodies, model organisms and tools, where possible.<br><br>Have you included the information requested as detailed in our Minimum Standards Reporting Checklist?	Yes
**Availability of data and materials**<br><br>All datasets and code on which the conclusions of the paper rely must be either included in your submission or deposited in publicly available repositories	Yes

(where available and ethically appropriate), referencing such data using a unique identifier in the references and in the “Availability of Data and Materials” section of your manuscript.<br><br>Have you have met the above requirement as detailed in our Minimum Standards Reporting Checklist?	

Manuscript

Click here to download Manuscript DataNote_R2-SarropoulouED.docx

Full Genome Survey and Dynamics of Gene Expression in the Greater Amberjack *Seriola dumerili*

Sarropoulou E[1*], Sundaram A.Y.M[2]., Kaitetzidou E[1] , Kotoulas G[1], Gilfillan G.D.[2], Papandroulakis N[1], Mylonas C.C[1]., Magoulas A.[1]

[1]Institute of Marine Biology, Biotechnology and Aquaculture, Hellenic Centre for Marine Research, Greece

[2]Department of Medical Genetics, Oslo University Hospital and University of Oslo, Oslo, Norway

[*]Corresponding author

[*]ES: sarris@hcmr.gr
AS: arvind.sundaram@medisin.uio.no
EK: ekaitetz@hcmr.gr
GK: kotoulas@hcmr.gr
GG: gregorg@medisin.uio.no
NP: npap@hcmr.gr
CM: mylonas@hcmr.gr
AM: magoulas@hcmr.gr

Abstract (250 words)

Background: Teleosts of the genus *Seriola*, commonly known as amberjacks, are of high commercial value in international markets due to their flesh quality and worldwide distribution. The *Seriola* species of interest to Mediterranean aquaculture is the greater amberjack (*Seriola dumerili*). This species holds great potential for the aquaculture industry, but in captivity, reproduction has proved to be challenging and observed growth dysfunction hinders their domestication. Insights into molecular mechanisms may contribute to a better understanding of traits like growth and sex, but investigations to unravel the molecular background of amberjacks have begun only recently.

Findings: Illumina HiSeq sequencing generated a high coverage greater amberjack genome sequence comprising 45,909 scaffolds. Comparative mapping to the Japanese yellowtail (*Seriola quinqueriadiata*) and to the model species medaka (*Oryzias latipes*) allowed the generation of *in silico* groups. Additional gonad transcriptome sequencing identified sex-biased transcripts, including known sex-determining and differentiation genes. Investigation of the muscle transcriptome of slow-growing individuals showed that transcripts involved in oxygen and gas transport were differentially expressed compared to fast/normal-growing individuals. On the other hand, transcripts involved in muscle functions were found to be enriched in fast/normal-growing individuals.

Conclusion: The present study provides first insights into the molecular background of male and female amberjacks and of fast and slow-growing fish. Therefore, valuable molecular resources have been generated in the form of a first draft genome and a reference transcriptome. Sex-biased genes which may also have roles in sex determination or differentiation, and genes that may be responsible for slow growth are suggested.

Keywords: *Seriola dumerili*, RNA-seq, Genome, Aquaculture, Differential expression, Correlation patterns, Gender expression pattern

Background information

Seriola species, belonging to the family Carangidae and commonly known as amberjacks, are of high commercial value and have a significant international market due to their first-rate flesh quality, fast growth and worldwide distribution. The main representatives of the family of interest to the growing aquaculture industry are the greater amberjack (*Seriola dumerili*, NCBI taxon ID*:* 41447, Fig.1), the Japanese yellowtail (*Seriola quinqueradiata*, NCBI taxon ID:8161), the yellowtail kingfish (*Seriola lalandi*, NCBI taxon ID*:*302047*)* and the longfin yellowtail (*Seriola rivoliana*, NCBI taxon ID: 173321) [1, 2]. However, in captivity, reproductive as well as growth dysfunction hinders their domestication. In addition, under captive conditions, fish may exhibit skewed sex ratios or precocious maturation before reaching market size. Teleost fishes are known to have a broad range of sex-determining mechanisms, which may differ even in closely related species, and many also show sexual dimorphism in growth. Consequently, sex control is one of the most important and highly targeted research fields in aquaculture. Concerning sex determination in the Carangidae species studied so far, no heteromorphic sex chromosome has been recorded [3]. Another important aspect in fish aquaculture is fish growth, which is a multifaceted physiological trait involving many different parameters. It can be influenced by nutrition, environment, as well as by genetic factors. Investigations to unravel the molecular background of these traits may contribute significantly to the development of reliable domestication technology.

The greater amberjack has become an attractive species for the Mediterranean industry for which to develop aquaculture practices due to its high growth rate. It represents the largest member of the family Carangidae [4], is a pelagic fish with a broad-based zoogeographical distribution and a tendency to inhabit reefs, wrecks and artificial structures such as oil platforms [5–7]. Like for the other Seriola species, greater amberjack reproduction in captivity has proved to be challenging [8]. Greater amberjacks do not show obvious sexual dimorphism, but, as in a number of other teleost fish species, the ability to distinguish the

sexes is an important factor for stock management and efficient fish farming. It has also been reported that growth in the greater amberjack is restricted in individuals reared in captivity. Slow-growing fish present a bottleneck in aquaculture, as small individuals have higher mortality rates, and if they were to comprise a significant number of the stock, they would contribute to inefficient farming. Insights into molecular mechanisms may lead to a better understanding of physiological traits such as growth and sex. To date, genetic resources for *Seriola* species have been developed mainly for yellowtail kingfish and the Japanese yellowtail, including genetic linkage maps [9,10], a radiation hybrid (RH) map [9], as well as the production of transcriptome data [11]. For the greater amberjack very few molecular resources have been published, but do include a cytogenetic characterization, which revealed in total 24 mainly acrocentric chromosomes (2n) and, similar to other Carangidae species, no morphologically differentiated sex chromosome [12]. The greater amberjack and the Japanese yellowtail are gonochoristic species and phylogenetic analysis showed that they diverged 55 mya [13]. For the Japanese yellowtail, it has been shown that sex is determined by the ZZ-ZW sex-determining system, and the sex-linked locus has been localized in linkage group (LG) 12 [9, 10].

Data description

Context

The present study reports for the first time gonad-specific gene expression, as well as differences between the muscle transcriptomes of slow-growing and fast/normal-growing amberjacks reared under cultured conditions. It further suggests by a comparative mapping approach a gender-specific genome region in the greater amberjack. Key molecular resources in the form of the first greater amberjack genome assembly, as well as transcriptome data for further functional studies, have therefore been generated.

Methods

All procedures such as handling and treatment of fish used during this study were performed according to the three Rs (Replacement, Reduction, Refinement) guiding principles for more ethical use of animals in testing, first described by Russell and Burch in 1959 (EU Directive 2010/63).

An overview of the complete workflow is given in Additional file 9.

Sampling

Blood, sperm and muscle sampling was performed at the aquaculture facilities of the Hellenic Centre for Marine Research (HCMR), Heraklion Crete. Blood samples obtained from adult fish were immediately placed in BD Vacutainer® Plastic K3 EDTA blood collection tubes (reference number 368857, BD, Franklin Lakes, NJ, USA). Muscle samples of slow-growing (n = 4 fish: 2 x 24 g and 2x 20 g) and fast/normal-growing (n = 4 fish: 60 g, 94 g, 106 g and 120 g) individuals were taken at the age of 5 months, transferred to tubes containing RNAlater and stored at -80 °C until processing. Gonad samples of four mature female and four male amberjacks (n = 4 fish) were received from fish maintained at an aquaculture facility in Salamina (Argosaronikos Fishfarming S.A., Salamina, Greece) during the peak of the reproductive season (end of May early June). At the time of sampling, fish were 4 years old and had a body size ranging between 9 and 17 kg [8]. The females were in advanced vitellogenesis, while the males were either in active spermatogenesis or contained luminal spermatozoa with only limited developing spermatocysts. Gonad samples were also kept in RNAlater and stored at -80 °C until processing.

High quality DNA extraction and genomic library preparation

Genomic DNA was extracted from one male and one female individual. High-quality female and male genomic DNA was retrieved from blood and sperm, respectively, following the

protocol of Qiagen DNeasy Blood and Tissue Kit. Genomic DNA libraries were prepared using TruSeq PCR-free library kit (Illumina, USA) following the manufacturer's recommendations with individual barcodes.

RNA extraction and library preparation

Total RNA was extracted from all samples using the Nucleospin miRNA Kit (Macherey-Nagel GmbH & Co. KG, Duren, Germany) according to the manufacturer's instructions. In brief, gonads and muscle tissues were disrupted in liquid nitrogen using mortar and pestle, dissolved in lysis buffer and passed through a 23-gauge (0.64 mm) needle five times to homogenize the mixture. RNA quantity was determined using a NanoDrop ND-1000 spectrophotometer (NanoDrop Technologies Inc, Wilmington, USA) and the quality was evaluated further by agarose (1 %) gel electrophoresis as well as by capillary electrophoresis (RNA Nano Bioanalyzer chips, Bioanalyzer 2100, Agilent, USA). All RNA libraries were prepared using the TruSeq stranded total RNA library kit (Illumina, USA). RNA libraries generated from eight different muscle samples were indexed with eight different barcodes to be run on one Illumina MiSeq lane, while RNA libraries generated from female and male gonads were indexed to be run on a HiSeq2500 (Illumina, USA).

Next generation sequencing

The two genome libraries (male and female gDNA) were pooled together and paired end (125 bp) sequenced over 66% of two lanes of HiSeq 2500 (Illumina, USA). Eight RNA-seq libraries from female and male gonads were multiplexed and also sequenced in one lane of a HiSeq 2500 with 125bp paired end reads. RNA libraries prepared from muscle tissues were 250 bp pair end sequenced in one run of a MiSeq (Illumina, USA). Raw bcl files were analyzed and de-multiplexed using the barcodes by RTA V1.18.61.0 and bcl2fastq v1.8.4 (bcl2fastq, RRID:SCR_015058).

Bioinformatic analysis

Pre-processing

Quality control of raw fastq files was assessed using the open source software FastQC version 0.10.0 (FastQC, RRID:SCR_014583) [46]. Pre-processing of reads was performed to remove adapter contamination followed by trimming of low-quality reads using Trimmomatic v0.33 (Trimmomatic, RRID:SCR_011848) software [47]. Reads mapping to PhiX Illumina spike-in were removed using bbmap v34.56 [48]. Reads longer than 36 nt were retained for further analyses.

Genome assembly

Cleaned data from male and female gDNA were concatenated and normalized to ~ 50 x coverage using the *in silico* read normalization tool in Trinity v2.0.6 (Trinity, RRID:SCR_013048) [49]. Resulting data was assembled using MaSuRCA v3.1.3 [50] using default parameters. The quality of the assembly was checked using BUSCO v3.0.2 (BUSCO, RRID:SCR_015008) [51] using Eukaryota_odb9 and zebrafish as lineage dataset and reference, respectively. Further analysis of the genome was performed calculating the k-mer content using KmerGenie v1.6982 [51, 52]. Kmer content was calculated for all trimmed data, 50x as well as 75x normalized data. GenomeScope vs 1.0 fast profiling [54] was used to assess the heterozygosity level.

Comparative mapping

Comparative mapping was applied in order to group the assembled scaffolds of the greater amberjack generated in the present study. Therefore, publicly available sequences of the Japanese yellowtail RH map [9], as well as the already established synteny of the Japanese yellowtail with medaka [17] were used as the backbone for the current comparative mapping

approach. Both species contain 24 chromosomes, similar to the greater amberjack; consequently, a one-to-one relationship could be established. Firstly, all available RH markers of the Japanese yellowtail were mapped using blastall 2.2.17 in BLAST toolkit and a stringent e-value of < 1E-10 to the greater amberjack reference transcriptome, as well as to the generated greater amberjack genome scaffolds. Scaffolds were grouped and named according to the linkage groups of the Japanese yellowtail. The greater amberjack reference transcriptome and the generated greater amberjack genome scaffolds were also mapped, as described above, to the medaka genome (downloaded from the Genome Browser Gateway - Oct. 2005 version 1.0 draft assembly equivalent to the Ensembl Oct. 2005 MEDAKA1 assembly) and validated by comparing homologous groups among the greater amberjack, the Japanese yellowtail and medaka. Scaffolds belonging to one chromosome of medaka and to the homologous group of the Japanese yellowtail were grouped together, sorted according to their match in medaka and concatenated in order to generate *in silico* groups in the greater amberjack. The reference transcriptome was mapped to the concatenated genome scaffolds (with % identity 100 % and e-value = 0) and the concatenated genome scaffolds were mapped on to the genome of medaka, three-spine stickleback and tetraodon (*Tetraodon nigroviridis*). Syntenic groups to the Japanese yellowtail and medaka were visualized by circos v0.69-3 (Circos, RRID:SCR_011798) [55] (Figure 2b, c).

Genome annotation and reference transcriptome assembly

Processed data from RNA samples were assembled using Trinity v2.0.6. Initially, the data were normalized to 50x coverage and then assembled using default parameters (--SS_lib_type RF). Relative abundance of each transcript/isoform was calculated using RSEM and transcripts with low coverage were filtered using filter_fasta_by_rsem_values.pl tool with the following parameters - tpm_cutoff 1, fpkm_cutoff 0 and isopct_cutoff 1. Two-pass iterative MAKER v2.31.8 (MAKER, RRID:SCR_005309) [56] was used to predict genes

from the generated genome assembly using the Trinity assembled transcriptome as EST evidence and the UniProt Sprot protein database (UniProt, RRID:SCR_002380) as protein homology evidence. HMM files created using SNAP v2006-07-28 [57] and GeneMark-ES Suite v4.21 [58] were used on the first pass for gene prediction and Augustus v3.0.1 (Augustus: Gene Prediction, RRID:SCR_008417) gene prediction species model based was used during the second pass to refine gene prediction.

Predicted protein sequences were annotated using blastp in BLAST v2.2.29 toolkit against NCBI nr database and using InterproScan against Interpro protein domains. Blast2GO v3.3.5 (Blast2GO, RRID:SCR_005828) was used to merge the two results and GO-mapping was performed using the same software. This reference transcriptome was used for differential expression analyses.

Differential expression analysis

Processed reads from four testis and four ovary samples were aligned against the assembled genome and predicted transcriptome using Tophat2 v2.0.13 (TopHat, RRID:SCR_013035) and reads mapping to genes (MAKER2 gtf) were counted using featureCounts v1.4.6-p1. Differential expression was calculated using DESeq2 v1.10.1 [59] in R v3.2.4 [60] with default methods implemented in the function 'DESeq' within this tool as the data is assumed to fit the Negative binomial generalized linear model. A similar pipeline was used to calculate differential expression between fast/normal and slow-growing individuals.

Data evaluation

Samples were clustered in order to detect possible outliers applying the WCGNA software package [61], which detects possible outliers based on their Euclidean distance (additional files 1 and 4). For further data evaluation, the biological replicates were validated by calculating the sample–to-sample distances, illustrated in the form of a heatmap between the

samples using the free available scripts within the DeSeq2 package. The heatmap of the distance matrix gives an overview of similarities and dissimilarities between the samples. Besides clustering using Euclidean distance, Principal component (PCA) 2D plot analysis was performed to show the overall effect of experimental covariates, as well as batch effects [62]. Finally, hierarchical clustering of significantly differentially expressed transcripts was performed to illustrate the up and downregulated transcripts.

Meta-analysis

Differentially expressed transcripts between male and female gonads, as well as between slow and fast/normal-growing individuals were annotated using BLAST search (version 2.2.25) [36] against the non-redundant protein database and non-redundant nucleotide database. Blast2GO (Blast2GO, RRID:SCR_005828) software [37] was applied to determine GO terms (cellular component, molecular function and biological process), as well as to perform enrichment analysis. Enrichment analysis was carried out using all assembled transcripts as the reference set, and the differentially expressed genes (male vs. female gonads and slow vs. fast/normal-growing individuals) as well as transcripts mapped to the *in silico* generated groups as test set. Default parameters were chosen, i.e. two tailed test and FDR<0.05.

Data Validation and quality control

Genome sequencing, assembly and annotation

Whole genome sequencing was performed on genomic DNA from one female and one male specimen of the greater amberjack, generating 345,544,307 150 bp paired end reads. After data pre-processing and *in silico* normalization 230,856,386 reads were obtained, which were further used to assemble the draft genome. Assembly of the genome produced a 669,638,422 bp (~670 Mb) genome represented in 45,909 scaffolds made up of 62,353 contigs. The

longest scaffold was 575,738 bp long with an N50 scaffold length of 75.1 kb and the N50 contig length of 36.6 kb. Kmer profiling (Additional file 1) showed that 50x normalized data had the same k-mer distribution as the original sequenced (all) data. Kmer distribution for 75x genome coverage did not resemble the original dataset. KmerGenie recommended the best kmer for 50x and all data as 89 and 79, respectively. MaSuRCA independently calculated a kmer profile and used 85 as the kmer value while assembling the 50x normalized data. Further genome analysis applying GenomeScope revealed a low heterozygosity level (0.649%). Maker2 analyses predicted 108,524 genes and 116,045 transcripts in the genome and after applying the recommended threshold [16] of AED (annotation Edit Distance) < 1 resulted in 45,547 and 53,023 high quality genes and transcripts, respectively. Out of 53,023 transcripts 33.6 % were successfully annotated using BLAST against NCBI non-redundant (nr) database with an e-value < 10^{-5}. BUSCO was used to evaluate the assembled genome and the transcriptome and out of 303 BUSCO groups (aka conserved orthologs) specific to eukaryotes, more than 93% were identified to be encoded by both genome and the transcriptome assembly (Table 1).

Comparative mapping

Using a comparative mapping approach (Fig. 2a), 468 Japanese yellowtail molecular markers retrieved from the publicly available Japanese yellowtail RH map were successfully mapped to 409 greater amberjack scaffolds (Table 2), while 14,990 greater amberjack scaffolds were successful mapped to the 24 chromosomes of medaka. This enabled the generation of *in silico* groups and subsequent synteny analysis. *In silico* generated groups of the greater amberjack were named according to the RH groups of the Japanese yellowtail (Fig. 2b) [9,17]. Out of the 53,023 obtained transcripts, 44,371 (~84%) were successfully mapped to the generated *in silico* groups of the greater amberjack and 30,342 transcripts (~57 %) to the genome of medaka (Fig. 2c, Table 2). In the Japanese yellowtail, LG12 has been identified as

the putative sex determining linkage group [15]. Transcripts mapping to the greater amberjack *in silico* group 12, mapped successfully to their homologous group of the Japanese yellowtail (LG12), medaka (chr. 8) and three-spine stickleback (chr.V and chr. XI) (Fig. 3a). Analysis of transcripts successfully mapped to the *in silico* group 12 (Additional file 2) revealed an enrichment for those involved in ubiquitination and de-ubiquitination (Fig. 3b, Additional file 3).

Gender-specific gene expression profiles

The gonadal transcriptome of four female and four male individuals sampled during the reproductive season were sequenced on the Illumina HiSeq platform, resulting in a total of 78,264,170 and 57,561,139 raw reads, respectively. After trimming and PhiX removal, approximately 80 % of the reads remained, and of which 70 % of these aligned to the generated genome using tophat2 (Table 3). Cluster analysis demonstrated a clear division of female and male gonad expression between the two sample groups (Additional file 4). Significantly differentially expressed transcripts (padj <0.005 and $log_2FC >|2|$) between genders amounted to 7,199 transcripts with 2,522 being higher expressed in female gonads and 4,677 in male gonads (Fig. 4b, Additional file 5). Principal component clustering (Fig. 4a), as well as hierarchical clustering (Fig. 4b), illustrated in the form of a heatmap, clearly showed again the separation of female and male gonad gene expression patterns. In addition, the latter revealed that the majority of transcripts had higher expression in male gonads in comparison to the female gonads. A total of 4,266 of the significant differentially expressed transcripts were successfully assigned to one of the generated greater amberjack *in silico* groups (Additional file 6). Enrichment analysis of transcripts more highly expressed in the male gonads resulted in GO terms involved in regulation but also in sex differentiation (Fig. 5a), while transcripts more highly expressed in the female gonads resulted in GO terms including mitochondrial translation and mitochondrial respiratory chain complex IV assembly (Fig. 5b).

Expression profiles of slow vs. fast/normal growing individuals

The muscle transcriptome of four slow and four fast/normal-growing individuals were sequenced on the Illumina MiSeq platform, resulting in a total of 10,625,681 and 9,005,157 raw reads, respectively. After preprocessing, approximately 85% of the reads remained, and of which about 50% of these aligned to the generated genome using tophat2 (Table 4). Outlier detection analysis led to the exclusion of one fast/normal grower from the downstream analysis (Additional file 7). Transcripts with p-values lower than 0.005 and more than a $\log_2$ FC were considered differentially expressed, resulting in 40 transcripts being upregulated in slow-growing individuals, and 52 transcripts being upregulated in fast/normal-growing individuals (Fig. 6). Enrichment analysis showed that transcripts upregulated in fast/normal growing individuals comprise GO terms related to muscle physiology (Fig. 7a). On the other hand, transcripts found to be upregulated in slow growing individuals were mainly found within the GO Biological Process terms "gas transport" and "oxygen transport" (Fig. 7b).

Re-use potential and discussion

The rapid growth and large size of greater amberjack, as well as its high-quality flesh and worldwide distribution has drawn the attention of the aquaculture sector. The development of appropriate and efficient husbandry practices for industrial production has, however, proved difficult. Insights into its molecular background may enhance the prospects of discovering important aquaculture-related traits, and consequently contribute to the more rapid development of appropriate husbandry practices. The present study includes a draft genome assembly of the greater amberjack of approximately 670 Mb, generated from 345,544,307 paired end reads obtained by Illumina sequencing. The genome size of the greater amberjack has been estimated to be 0.74 pg [18]. Hence, it is anticipated that its genome has been sequenced to approximately 75 x coverage in this study. Similar genome sizes have been reported for two other aquaculture species important in the Mediterranean , the gilthead sea

bream (*Sparus aurata*) [19] and the European sea bass (*Dicentrarchus labrax*) [20]. While the genome of the gilthead sea bream has still not been published, the genome of the European sea bass (v1.0c) has been sequenced to approximately 30 x coverage by Sanger, 454 and Illumina sequencing [21]. The draft assembly of the European sea bass genome and the draft assembly of the greater amberjack produced similar N50 contig lengths (54 kb and 37 kb, respectively), but differ significantly in N50 scaffold length. For the European sea bass genome an N50 scaffold length of 4.9 Mb has been reported, while the N50 scaffold length for the greater amberjack described here is only 75 kb. This result is not surprising, as here only Illumina paired end sequencing has been performed. To increase scaffold length, the addition of longer reads from a second technology would be necessary. Nonetheless, the obtained N50 scaffold length here compares favorably to those of other teleost species (i.e. *Chatrabus melanurus, Chaenocephalus aceratus* and *Bregmaceros cantori*), which have been sequenced to a similar depth with only Illumina paired end data and generated N50 scaffold lengths as low as 7 kb [22].

Like the chromosome number in the Japanese yellowtail, previous cytogenetic analysis determined the haploid number of chromosomes in the greater amberjack to be 24 [12]. Applying a comparative mapping approach based on previously published genetic linkage and RH maps of Japanese yellowtail [13, 14, 22] as well as to the medaka genome, the generated greater amberjack scaffolds were clustered successfully to 24 *in silico* groups (Table 2, Fig. 2b). Subsequent mapping of the generated greater amberjack transcripts to the medaka genome and to the generated greater amberjack *in silico* groups resulted in a one-to-one relationship (Fig. 2c).

Comparative mapping allows the identification of markers for traits of interest either based on candidate genes, or based on previous QTL studies in the same or in other species. In this way, a sex-linked locus was found in LG12 of the Japanese yellowtail [15]. Transcripts mapped to the greater amberjack *in silico* group 12 mapped to medaka Chr.8 and

three-spine stickleback Chr.V and XI (Fig. 3a, Additional file 8), although in neither case, have these been reported as sex determining chromosomes [23, 24]. In the Japanese yellowtail the sex-linked locus was without doubt linked to LG12, but despite this and the generation of a second, increased resolution genetic linkage map [14], the sex determining-genes, known up-to date, have not been identified in the sex-determining region of the Japanese yellowtail. The authors speculated that the PDZ domain containing GIPC1 protein, found in the SD region of LG12, may be of importance in determining sex in the Japanese yellowtail. This protein was also found in the greater amberjack *in silico* group 12, but without being differentially expressed between the female and the male gonads (Additional file 2). The greater amberjack is a gonochoristic species, without any external sexual dimorphism, and genetically differentiated sex chromosomes have not yet been identified [12]. In teleost fishes, a broad range of sex determining mechanisms have been documented, and different sex-determining genes have been reported (for a review see [28]). The main sex-determining genes known in other teleosts were found to be located in the greater amberjack *in silico* group 1 (amhr2), group 4 (amhY), group 7 (dmY/dmrt1a), group 17 (gsdf) and group 18 (sox3Y) (Table 5). Enrichment analysis of transcripts successfully mapped in the present study to the homologous *in silico* group 12 of the greater amberjack resulted mainly in Biological Process GO terms related to ubiquitination (Fig. 3b). A recent and growing body of evidence points to the important role of ubiquitination in the regulation of spermatogenesis from the very beginning up to spermatid differentiation [26–28]. On the other hand, transcripts involved in ubiquination have been reported in the ovary transcriptome of the striped bass (*Morone saxtilis*) [29, 30]. Analogous enrichment analysis of the remaining greater amberjack *in silico* groups did not reveal any GO terms specific to sex regulation (Additional file 3). The present findings may indicate the importance of ubiquitination during sex determination.

In addition to genome sequencing, the gender specific mechanisms at the transcriptome level operating in the greater amberjack were investigated. It has to be noted, that a link between sex determination and sex specific expression is neither necessary nor expected. At this point, gonadal specific transcripts were identified, with more transcripts found to be highly expressed in male than in female gonads (Fig. 4a and b, Additional file 5).

Among the female gonads biased transcripts, 12 transcripts were identified belonging to the zona pellucida (zp) proteins (Additional file 5). It has been shown that during oocyte development, the oocyte is surrounded by an acellular envelope comprising zp proteins [31–34]. Also in other transcriptomic studies in fish, it has been reported that zp proteins are more highly expressed in the female gonads (e.g. [33]). Another well-known gene family, identified as being involved in sex differentiation are cathepsins. Cathepsins are responsible for the degradation of vitellogenin into yolk proteins [36]. In the present study, seven transcripts were identified as being differentially expressed, with five of them being more highly expressed in female gonads (Additional file 5). Cathepsin S and z-like showed the highest $\log_2$ FC (~ 10). Cathepsin S has also been reported to be more highly expressed in the female olive flounder (*Paralichthys olivaceus*) [35]. Furthermore, the well-documented ovary marker for teleost species, cytochrome P450 aromatase gene, cyp19a [37], was also identified in the present study as being more highly expressed in the female gonads. Enzymes encoded by cyp450 genes play an important role in the synthesis and metabolisms of steroid hormones, as well as of certain fats and acids used to digest fats. The differentially expressed transcripts encoding for cyp450 genes in the present study comprised 7 cyp450 transcripts more highly expressed in female, and 6 more highly expressed in male gonads (Fig. 8a). Interestingly, cyp4502f2 was more highly expressed in male while cyp450 2f2-like protein was higher expressed in female gonads. Cyp4502f2 belongs to the gene family encoding monooxygenase activity that is important for detoxification. To date, cyp4502f2 has been found mainly to be expressed in the liver and lung [38], while its expression in gonads has

not yet been reported. It is also known that cyp450 genes are involved in the retinoid acid (RA) pathway, which is important in ovarian differentiation. Among the cyp450 genes, cyp26 enzymes contribute to the regulation of RA level. Interestingly, cyp26 has two paralogous genes, cyp26a1 and cyp26b1, which were found with opposite gender-expression pattern in the greater amberjack (Fig. 8). This has also been shown in the hermaphrodite species bluehead wrasse (*Thalassoma bifasciatum*) [39], but also in Nile tilapia (*Oreochromis niloticus*) [40] and mice (*Mus musculus*) [41].

Forkhead box protein L2 (*foxl2*) has also been shown to have an important role in female sex differentiation [42]. Forkhead box proteins are transcription factors with significant regulatory roles during development, cell growth proliferation and differentiation. To our best knowledge, among foxl proteins, foxl2 has been reported as being involved during ovarian differentiation [35], and foxl3 has been detected as having gender-biased expression in fish [39]. In the present work a total of 14 transcripts encoding for forkhead box proteins were identified as having a gender-specific expression patterns, including foxl2 being more highly expressed in female and foxl3 being more highly expressed in male gonads. (Fig. 8b). Interestingly, in mice it has been speculated that elevated cyp26b1 levels uphold the male fate of germ cells in testes, and foxl2 antagonizes cyp26b1 expression in ovaries [43]. Both genes also showed expression in the present study consistent with this, indicating that the hypothesis that the RA signaling pathway may play a significant role in gonadal sex change regulation in hermaphroditic fish [39] may also be true for gonochoristic species.

In addition, the present study is also the first to attempt to assess the molecular background of slow-growing vs. fast/normal-growing greater amberjacks. White muscle was selected to investigate differential expression analysis, as it comprises the majority of the myotome and consequently it is expected to isolate mainly transcripts encoding for structural proteins involved in myogenesis and growth [44]. In the present study, transcripts mainly involved in processes affecting muscle physiology were found to be enriched in fast /normal-growing

individuals (Fig.7). On the other hand, enrichment analysis of transcripts significantly upregulated in slow-growing individuals revealed that these mainly activated the processes of gas and oxygen transport (Fig. 7b). Similar results were also reported in a recent study of slow vs. fast-growing rainbow trout (*Oncorhynchus mykiss*) [45]. In comparison with the present study, slow-growing rainbow trout showed elevated mitochondrial and cytosolic creatine kinase expression levels whereas fast-growing fish revealed an elevated cytoskeletal gene component expression level. Growth is in general a multifaceted process and comprises many interacting factors. The fact that the present study identified a clear expression pattern between the two groups by applying a medium throughput Illumina platform points to the noteworthy possibility of applying low-depth RNA-seq, available to a number of small laboratories, in order to gain first insights into important physiological processes.

Conclusion

The present study has provided the first insights to the molecular background of male and female individuals as well as of fast and slow-growing fish, of the greater amberjack. By this means, the genome of an important new aquaculture fish species was reported, as well as the gonad and muscle transcriptomes. Illumina HiSeq sequencing generated a high coverage genome sequence comprising 45,909 scaffolds. Comparative mapping to the Japanese yellowtail, as well as to the model fish species medaka, allowed the generation of *in silico* groups comprising 83% of the obtained transcripts. Transcripts found to be more highly expressed in male and in female gonads were identified, and comprised known sex-determining and sex-differentiation genes. Further differential expression analysis of fast/normal vs. slow-growing amberjacks points to an important role of oxygen and gas transport in relation to slow-growing individuals, whereas in fast/normal-growing fish important transcripts involved in muscle function are significantly upregulated.

Figure legends

Figure 1: Image of the greater amberjack (*Seriola dumerilii*)

Figure 2: Overview of comparative mapping approach. **a** workflow for the generation of *in silico* groups of the greater amberjack (*Seriola dumerilii*). **b** circos illustration of mapping results between the greater amberjack (blue) and mapping results of transcripts to the Japanese yellowtail (orange).**c** circos illustration of transcript mapping results between the greater amberjack (blue) and mapping results of transcripts to medaka (orange).

Figure 3: Identification by synteny of a putative amberjack sex-determination *in silico* linkage group. **a** putative sex linked group, *in silico* group 12 of the greater amberjack, compared to medaka, Japanese yellowtail and three-spine stickleback. **b** word cloud illustration of transcripts mapped to SD12 as test set and all identified transcripts as reference set.

Figure 4: Overview of transcriptome study of female vs. male gonads. **a** PCA plot of transcripts significantly differentially expressed. **b** heatmap of transcripts significantly (padj <0.005 and $\log_2$ FC >|2|) differentially expressed. Green color represents upregulated transcripts in male gonads while red color signifies upregulated transcripts in female gonads.

Figure 5: Word cloud illustration of significant enriched GO terms of the category Biological Process. **a**. enriched GO terms of transcripts upregulated in male gonads. **b**. enriched GO terms of transcripts downregulated in male gonads.

Figure 6: Overview of transcriptome study of slow growing vs. fast/normal-growing amberjacks. **a** PCA plot of transcripts significantly differentially expressed. **b** heatmap of

transcripts significantly differentially expressed. Green color represents upregulated transcripts in fast/normal-growing individuals while red color signifies upregulated transcripts in slow-growing individuals.

Figure 7: Word cloud illustration of significant enriched GO terms. **a**. enriched GO terms of transcripts upregulated in fast/normal-growing individuals vs. slow growing-individuals. **b**. enriched GO terms of transcripts upregulated in slow growing individuals vs. fast/normal-growing individuals.

Figure 8: Gene expression displayed as heatmaps of two gene families comprising significantly differentially expressed genes in female and male gonads. **a** Cytochrome P450 family **b.** Forkhead box protein family.

Additional files:

Additional file 1: a. K-mer profile plot using GenomeScope fast reference-free genome profiling method showing the fit of the model to the observed kmer frequency. The shape of the kmer profile reflects the complexity of the genome. A homozygote repeat-free genome results in a k-mer profile with a Poisson distribution. A two peak profile indicated a heterozygous genome. **b.** Kmer profile plot: all data (red), 75x (grey) and 50x normalised data (black). KmerGenie recommended the best kmer for 50x and all data as 89 and 79, respectively. MaSuRCA calculated its own kmer profile and used 85 as the kmer value while assembling the 50x normalised data. The plot was generated with the haploid model in KmerGenie. 50x and 75x normalisation was performed using Trinity normalisation tool. Dotted lines represent the kmer calculated for all, 75x and 50x data. Dashed line represents the kmer predicted and used by MaSuRCA.

Additional file 2: Annotated transcripts mapped onto the *in silico* group 12 of greater amberjack along with their expression values i.e. fold changes of transcripts significantly, more highly expressed in female gonads and in male gonads. (XLSX 32 kb).

Additional file 3: Illustration in form of word cloud of enrichment analysis of transcripts successfully mapped onto the *in silico* generated greater amberjack groups. (DOCX 1093 kb).

Additional file 4: a. Sample clustering for outlier detection resulting from RNA sequencing of female and male gonads. **b.** Sample-to-sample distances**.** Heatmap generated with DeSeq2 software packages showing the Euclidean distances between the samples. (PPTX 55 kb).

Additional file 5: Count file of individual data values showing transcripts significantly higher expressed in female gonads and in male gonads with DEG threshold padj<0.005 |log2FC| >2 along with their putative annotations. (XLSX 904 kb).

Additional file 6: Transcripts significantly higher expressed in female gonads and in male gonads along with their fold change value as well as their position within the generated *in silico* groups of greater amberjack. (XLSX 229 kb).

Additional file 7: a. Fish weight of slow and fast/normal growing-individuals. **b.** Sample clustering for outlier detection resulting from RNA sequencing of fast /normal vs slow-growing individuals. (PPTX 65 kb).

Additional file 8: Illustration of comparative mapping approach of Japanese yellowtail with medaka and three-spine stickleback respectively. (PPTX 1006 kb).

Additional file 9: Workflow overview (PPTX 87 kb).

Tables

Table 1: BUSCO results

	Genome		Trancriptome	
Complete BUSCOs	284	93.7 %	283	93.4 %
Complete and single-copy BUSCOs	270	89.1 %	219	72.3 %
Complete and duplicated BUSCOs	14	4.6 %	64	21.1 %
Fragmented BUSCOs	8	2.6 %	13	4.3 %
Missing BUSCOs	11	3.6 %	7	2.3 %
Total BUSCO groups searched	303		303	

Table 2. Comparative mapping of greater amberjack scaffolds and transcripts to the Japanese yellowtail RH map and to the medaka genome.

Japanese yellowtail RH Group	Number of Japanese yellowtail markers mapped to greater amberjack scaffolds	Homologous medaka chromosomes	Number of greater amberjack transcripts mapped to the medaka genome
SQ1	30	OL5	1451
SQ2	19	OL1	1425
SQ3	12	OL6	1423
SQ4	19	OL4	1490
SQ5	14	OL23	777
SQ6	26	OL21	1197
SQ7	13	OL19	1046
SQ8	16	OL15	1204
SQ9	25	OL3	1307
SQ10	17	OL11	1269
SQ11	12	OL2	689
SQ12	36	OL8	1575
SQ13	5	OL17	1642
SQ14	12	OL13	1378
SQ15	32	OL9	1534
SQ16	18	OL16	1514
SQ17	17	OL12	1233
SQ18	16	OL10	1095
SQ19	24	OL14	1318

SQ20	16	OL22	1350
SQ21	17	OL20	993
SQ22	17	OL18	816
SQ23	23	OL24	1139
SQ24	31	OL7	1477

SQ: *Seriola quinqueradiata* OL: *Oryzias latipes*

Table 3. RNA sequencing reads derived from four female gonads (F) and four male gonads (M) by Illumina HiSeq sequencing.

	**Raw reads**	**Trimmed reads**	**After PhiX removal**	**% of raw reads used downstream analyses**	**Tophat2 align**	
F1	17,268,356	14,425,361	14,391,074	83.34%	10,210,127	70.9%
F2	17,083,394	13,542,326	13,516,020	79.12%	9,601,965	71.0%
F3	23,331,846	19,330,707	19,265,030	82.57%	13,835,238	71.8%
F4	20,580,574	16,881,832	16,834,901	81.80%	12,066,021	71.7%
M1	13,860,606	11,838,653	11,805,696	85.17%	8,603,874	72.9%
M2	15,315,961	12,898,261	12,868,488	84.02%	9,205,543	71.5%
M3	15,086,732	12,036,225	11,997,908	79.53%	8,623,374	71.9%
M4	13,297,840	10,635,465	10,600,787	79.72%	7,548,436	71.2%

F: female gonads, M: male gonads

Table 4. RNA sequencing reads derived from muscle tissue of fast/normal and slow growing individuals by Illumina MiSeq sequencing.

	**Raw reads**	**Trimmed reads**	**After PhiX removal -**	**% of raw reads used for downstream analyses**	**Tophat2 align**	
Fast1	2,434,221	2,164,923	2,148,193	88.25%	914,312	42.56%
Fast2	2,335,103	2,004,168	1,990,068	85.22%	921,965	46.33%
Fast3	2,944,122	2,649,012	2,627,657	89.25%	1,265,753	48.17%
Fas4	2,912,235	2,616,056	2,600,521	89.30%	1,304,009	50.14%
Slow1	2,088,813	1,843,495	1,830,095	87.61%	868,946	47.48%
Slow2	2,263,688	1,941,004	1,925,396	85.06%	985,902	51.21%
Slow3	2,497,198	2,197,131	2,178,922	87.25%	958,565	43.99%
Slow4	2,155,458	1,892,706	1,877,917	87.12%	869,033	46.28%

Table 5: Overview of known sex determining regions in Teleost species identified in the greater amberjack.

**Gene**	**Abbreviation**	**Teleost accession number**	**Transcript in the greater amberjack**	***in silico* group**	***DE male vs female gonads**	**sex determining in**
DM-domain gene on the Y chromosome / doublesex and mab-3 related transcription factor 1a	*dmY/ dmrt1a*	*Oryzias latipes* NM_001104680 / XM_004086451 unplaced scaffold	maker-jcf7180000931253-snap-gene-0.40-mRNA-1	SD7	up in male gonads	***Oryzias latipes*** [23,65]
gonadal soma derived factor	*gsdf*	*Oryzias latipes* NM_001177742 chr.12	Augustus-masked-jcf7180000916295-processed-gene-0.7-mRNA-1	SD17	up in male gonads	***Oryzias luzonensis*** [66]
Y chromosome specific anti-muellerian hormone	*amhY*	*Oryzias latipes* NM_001104728 chr. 4	maker-jcf7180000931145-snap-gene-0.73-mRNA-2	SD4	up in male gonads	***Odontesthes hatcheri*** [67] *HM153803.1*
anti-muellerian hormone receptor 2	*amhr2*	*Lates calcarifer* KR492510 *Oryzias latipes* DQ499644.1 chr. 5 or 7	maker-jcf7180000889430-snap-gene-0.77-mRNA-5	SD1	no expression	***Takifugu genus*** [68]
sexually dimorphic o the Y chromosome	*sdY*	*Salmonidae* family	n/a	n/a	n/a	***Salmonidae family*** [69]
SRY-box containing protein 3Y	*sox3Y*	Oryzias latipes AJ245396 chr.10	augustus-masked-jcf7180000920392-processed-gene-0.31-mRNA-1	SD18	up in female gonads	***Oryzias dancena*** [65]

DE: differential expression, SD: *Seriola dumerili*

* Greater amberjack data

References

1. Benetti DD, Nakada M, Minemoto Y, Hutchinson W. Aquaculture of yellowtail amberjacks Carangidae: Current status, progress and constraints. Aquac. 2001 B. Abstr. 2001;56.

2. Holthus, A. Lovatelli PF. Capture-based aquaculture of yellowtail. Capture-Based Aquac. Glob. Overview. FAO Fish. Tech. Pap. 2008;No. 508:199–215.

3. X. C, X. L, R. L, S C. Karyotype analyis of the yellowtail kingfish *Seriola lalandi lalandi* (Perciformes:Carangidae) from South Australia. Aquac. Res. 2009;40:1735–41.

4. Hoese HD, Moore R.H. Fishes of the Gulf of Mexico: Texas, Louisiana, and adjacent waters. Texas A&M Univ. Press. Coll. Station. TX. 1977;327.

5. Manooch CS, Potts JC. Age, growth, and mortality of greater amberjack, *Seriola dumerili*, from the U.S. Gulf of Mexico headboat fishery. Bull. Mar. Sci. 1997;61:671–83.

6. Manooch CS, Potts JC. Age, growth and mortality of greater amberjack from the southeastern United States. Fish. Res. 1997;30:229–40.

7. Thompson BA, Beasley M, Wilson CA. Age distribution and growth of greater amberjack, *Seriola dumerili*, from the north-central Gulf of Mexico. Fish. Bull. 1999;97:362–71.

8. Zupa R, Rodriguez C, Mylonas C, Rosenfeld H, Fakriadis I, Papadaki M, et al. Comparative Study of Reproductive Development in Wild and Captive-Reared Greater Amberjack *Seriola dumerili* (Risso, 1810). PLoS One. 2017.

9. Aoki J, Kai W, Kawabata Y, Ozaki A, Yoshida K, Tsuzaki T, et al. Construction of a radiation hybrid panel and the first yellowtail (*Seriola quinqueradiata*) radiation hybrid map

using a nanofluidic dynamic array. BMC Genomics. 2014;15:165.

10. Ohara E, Nishimura T, Nagakura Y, Sakamoto T, Mushiake K, Okamoto N. Genetic linkage maps of two yellowtails (*Seriola quinqueradiata* and *Seriola lalandi*). Aquaculture. 2005;244:41–8.

11. Patel A, Dettleff P, Hernandez E, Martinez V. A comprehensive transcriptome of early development in yellowtail kingfish (*Seriola lalandi*). Mol. Ecol. Resour. 2016;16:364–76.

12. Sola L, Cipelli O, Gornung E, Rossi AR, Andaloro F, Crosetti D. Cytogenetic characterization of the greater amberjack, *Seriola dumerili* (Pisces: Carangidae), by different staining techniques and fluorescence in situ hybridization. Mar. Biol. 1997;128:573–7.

13. Swart BL, von der Heyden S, Bester-van der Merwe A, Roodt-Wilding R. Molecular systematics and biogeography of the circumglobally distributed genus Seriola (Pisces: Carangidae). Mol. Phylogenet. Evol. 2015;93:274–80.

14. Koyama T, Ozaki A, Yoshida K, Suzuki J, Fuji K, Aoki J ya, et al. Identification of Sex-Linked SNPs and Sex-Determining Regions in the Yellowtail Genome. Mar. Biotechnol. 2015;17:502–10.

15. Fuji K, Yoshida K, Hattori K, Ozaki A, Araki K, Okauchi M, et al. Identification of the sex-linked locus in yellowtail, *Seriola quinqueradiata*. Aquaculture. 2010;308.

16. Holt C, Yandell M. MAKER2: an annotation pipeline and genome-database management tool for second-generation genome projects. BMC Bioinformatics. 2011;12:491.

17. Aoki J, Kai W, Kawabata Y, Ozaki A, Yoshida K, Koyama T, et al. Second generation physical and linkage maps of yellowtail (*Seriola quinqueradiata*) and comparison of synteny with four model fish. BMC Genomics. 2015;16:406.

18. Hardie DC, Hebert PD. Genome-size evolution in fishes. Can. J. Fish. Aquat. Sci. 2004;61:1636–46.

19. Garrido-Ramos MA, Jamilena M, Lozano R, Cárdenas S, Rejón CR, Rejón MR. Cytogenetic analysis of gilthead seabream *Sparus aurata* (pisces, perciformes), a deletion

affecting the NOR in a hatchery stock. Cytogenet. Genome Res. 1995;68:3–7.

20. Aref'yev VA. Cytogenetic Analysis and Nuclear Organization of the Sea Bass Dicentrachus labrax. J. Ichthyol. 1990;1–12.

21. Tine M, Kuhl H, Gagnaire P-A, Louro B, Desmarais E, Martins RST, et al. European sea bass genome and its variation provide insights into adaptation to euryhalinity and speciation. Nat. Commun. 2014;5:5770.

22. Malmstrom M, M. M, OK. T, Jakobsen KS, S. J. Whole genome sequencing data and de novo draft assemblies for 66 teleost species. Sci. data. 2017;4.

23. Matsuda M, Nagahama Y, Shinomiya A, Sato T, Matsuda C, Kobayashi T, et al. DMY is a Y-specific DM-domain gene required for male development in the medaka fish. Nature. 2002;417:559–63.

24. Peichel CL, Ross JA, Matson CK, Dickson M, Grimwood J, Schmutz J, et al. The master sex-determination locus in threespine sticklebacks is on a nascent Y chromosome. Curr. Biol. 2004;14:1416–24.

25. Martínez P, Viñas AM, Sánchez L, Díaz N, Ribas L, Piferrer F. Genetic architecture of sex determination in fish: Applications to sex ratio control in aquaculture. Front. Genet. 2014.

26. Suresh B, Lee J, Hong SH, Kim KS, Ramakrishna S. The role of deubiquitinating enzymes in spermatogenesis. Cell. Mol. Life Sci. 2015. p. 4711–20.

27. Baarends WM, Hoogerbrugge JW, Roest HP, Ooms M, Vreeburg J, Hoeijmakers JH., et al. Histone Ubiquitination and Chromatin Remodeling in Mouse Spermatogenesis. Dev. Biol. 1999;207:322–33.

28. Sheng K, Liang X, Huang S, Xu W. The role of histone ubiquitination during spermatogenesis. Biomed Res. Int. 2014.

29. Reading BJ, Chapman RW, Schaff JE, Scholl EH, Opperman CH, Sullivan C V. An ovary transcriptome for all maturational stages of the striped bass (*Morone saxatilis*), a highly advanced perciform fish. BMC Res. Notes. 2012;5:111.

30. Chapman RW, Reading BJ, Sullivan C V. Ovary transcriptome profiling via artificial intelligence reveals a transcriptomic fingerprint predicting egg quality in striped bass, Morone saxatilis. PLoS One. 2014;9.

31. Modig C, Modesto T, Canario A, Cerdà J, Von Hofsten J, Olsson P-E. Molecular characterization and expression pattern of zona pellucida proteins in gilthead seabream (*Sparus aurata*). Biol. Reprod. 2006;75:717–25.

32. Wassarman PM. Zona pellucida glycoproteins. J. Biol. Chem. 2008. p. 24285–9.

33. Lyons CE, Payette KL, Price JL, Huang RCC. Expression and structural analysis of a teleost homolog of a mammalian zona pellucida gene. J. Biol. Chem. 1993;268:21351–8.

34. Wassarman P, Chen J, Cohen N, Litscher E, Liu C, Qi H, et al. Structure and function of the mammalian egg zona pellucida. J. Exp. Zool. 1999;285:251–8.

35. Fan Z, You F, Wang L, Weng S, Wu Z, Hu J, et al. Gonadal transcriptome analysis of male and female olive flounder (*Paralichthys olivaceus*). Biomed Res. Int. 2014;2014.

36. Sire M-F, Babin PJ, Vernier J-M. Involvement of the lysosomal system in yolk protein deposit and degradation during vitellogenesis and embryonic development in trout. J. Exp. Zool. 1994;269:69–83.

37. Guiguen Y, Fostier A, Piferrer F, Chang CF. Ovarian aromatase and estrogens: A pivotal role for gonadal sex differentiation and sex change in fish. Gen. Comp. Endocrinol. 2010;165:352–66.

38. Renaud HJ, Cui JY, Khan M, Klaassen CD. Tissue distribution and gender-divergent expression of 78 cytochrome p450 mRNAs in mice. Toxicol. Sci. 2011;124:261–77.

39. Liu H, Lamm MS, Rutherford K, Black MA, Godwin JR, Gemmell NJ. Large-scale transcriptome sequencing reveals novel expression patterns for key sex-related genes in a sex-changing fish. Biol. Sex Differ. 2015;6:26.

40. Feng R, Fang L, Cheng Y, He X, Jiang W, Dong R, et al. Retinoic acid homeostasis through aldh1a2 and cyp26a1 mediates meiotic entry in Nile tilapia (*Oreochromis niloticus*).

Sci. Rep. 2015;5:10131.

41. MacLean G, Li H, Metzger D, Chambon P, Petkovich M. Apoptotic extinction of germ cells in testes of Cyp26b1 knockout mice. Endocrinology. 2007;148:4560–7.

42. Ottolenghi C, Omari S, Garcia-Ortiz JE, Uda M, Crisponi L, Forabosco A, et al. Foxl2 is required for commitment to ovary differentiation. Hum. Mol. Genet. 2005;14:2053–62.

43. Kashimada K, Svingen T, Feng C-W, Pelosi E, Bagheri-Fam S, Harley VR, et al. Antagonistic regulation of Cyp26b1 by transcription factors SOX9/SF1 and FOXL2 during gonadal development in mice. FASEB J. 2011;25:3561–9.

44. Garcia de la Serrana D, Estevez A, Andree K, Johnston IA. Fast skeletal muscle transcriptome of the gilthead sea bream (*Sparus aurata*) determined by next generation sequencing. BMC Genomics. 2012/05/15 ed. 2012;13:181.

45. Danzmann RG, Kocmarek AL, Norman JD, Rexroad CE, Palti Y. Transcriptome profiling in fast versus slow-growing rainbow trout across seasonal gradients. BMC Genomics. 2016;17:60.

46. Http://www.bioinformatics.babraham.ac.uk/projects/fastqc. Fastqc.

47. Bolger AM, Lohse M, Usadel B. Trimmomatic: A flexible trimmer for Illumina sequence data. Bioinformatics. 2014;30:2114–20.

48. Bushnell B. BBMap (version 35.14). Available at https://sourceforge.net/projects/bbmap/. 2015

49. Grabherr MG., Brian J. Haas, Moran Yassour Joshua Z. Levin, Dawn A. Thompson, Ido Amit, Xian Adiconis, Lin Fan, Raktima Raychowdhury, Qiandong Zeng, Zehua Chen, Evan Mauceli, Nir Hacohen, Andreas Gnirke, Nicholas Rhind, Federica di Palma, Bruce W. N, Friedman and AR. Trinity: reconstructing a full-length transcriptome without a genome from RNA-Seq data. Nat. Biotechnol. 2013;29:644–52.

50. Zimin A V., Marçais G, Puiu D, Roberts M, Salzberg SL, Yorke JA. The MaSuRCA genome assembler. Bioinformatics. 2013;29:2669–77.

51. Simão FA, Waterhouse RM, Ioannidis P, Kriventseva E V., Zdobnov EM. BUSCO: Assessing genome assembly and annotation completeness with single-copy orthologs. Bioinformatics. 2015;31:3210–2.

52. Https://doi.org/10.1093/bioinformatics/btt310 H bx. psu. edu/. KmerGenie v1.6982.

53. Chikhi R, Medvedev P. Informed and automated k-mer size selection for genome assembly. Bioinformatics. 2014;30:31–7.

54. Vurture GW, Sedlazeck FJ, Nattestad M, Underwood CJ, Fang H, Gurtowski J, et al. GenomeScope: fast reference-free genome profiling from short reads. Bioinformatics. 2017;1–3.

55. Krzywinski M, Schein J, Birol I, Connors J, Gascoyne R, Horsman D, et al. Circos: An information aesthetic for comparative genomics. Genome Res. 2009;19:1639–45.

56. Campbell MS, Holt C, Moore B, Yandell M. Genome Annotation and Curation Using MAKER and MAKER-P. Curr. Protoc. Bioinforma. 2014;2014:4.11.1–4.11.39.

57. Grenon P, Smith B. SNAP and SPAN: Towards dynamic spatial ontology. Spat. Cogn. Comput. 2004;1:69–103.

58. Borodovsky M, Lomsadze A. Eukaryotic gene prediction using GeneMark.hmm-E and GeneMark-ES. Curr. Protoc. Bioinformatics. 2011;Chapter 4:Unit 4.6.1–10.

59. Love MI, Huber W, Anders S. Moderated estimation of fold change and dispersion for RNA-seq data with DESeq2. Genome Biol. 2014;15:550.

60. R Development Core Team R. R: A Language and Environment for Statistical Computing. R Found. Stat. Comput. 2011.

61. Langfelder P, Horvath S. WGCNA: an R package for weighted correlation network analysis. BMC Bioinformatics. 2008;9:559.

62. Metsalu T, Vilo J. ClustVis: A web tool for visualizing clustering of multivariate data using Principal Component Analysis and heatmap. Nucleic Acids Res. 2015;43:W566–70.

63. Altschul SF, Gish W, Miller W, Myers EW, Lipman DJ. Basic local alignment search

tool. J. Mol. Biol. 1990;215:403–10.

64. Conesa A, Götz S. Blast2GO: A Comprehensive Suite for Functional Analysis in Plant Genomics. Int. J. Plant Genomics. 2008;2008:619832.

65. Takehana Y, Matsuda M, Myosho T, Suster ML, Kawakami K, Shin-I T, et al. Co-option of Sox3 as the male-determining factor on the Y chromosome in the fish *Oryzias dancena*. Nat. Commun. 2014;5.

66. Myosho T, Otake H, Masuyama H, Matsuda M, Kuroki Y, Fujiyama A, et al. Tracing the emergence of a novel sex-determining gene in medaka, *Oryzias luzonensis*. Genetics. 2012;191:163–70.

67. Hattori RS, Murai Y, Oura M, Masuda S, Majhi SK, Sakamoto T, et al. A Y-linked anti-Mullerian hormone duplication takes over a critical role in sex determination. Proc. Natl. Acad. Sci. 2012;109:2955–9.

68. Kamiya T, Kai W, Tasumi S, Oka A, Matsunaga T, Mizuno N, et al. A trans-species missense SNP in Amhr2 is associated with sex determination in the tiger Pufferfish, *Takifugu rubripes* (Fugu). PLoS Genet. 2012;8.

69. Yano A, Guyomard R, Nicol B, Jouanno E, Quillet E, Klopp C, et al. An immune-related gene evolved into the master sex-determining gene in rainbow trout, *Oncorhynchus mykiss*. Curr. Biol. 2012;22:1423–8.

70. Sarropoulou E, Sundaram AY., Kaitetzidou E, Kotoulas G, Gilfillan GD, Papandroulakis N, et al. Supporting data for “Full Genome Survey and Dynamics of Gene Expression in the Greater Amberjack, *Seriola dumerili*”. GigaScience Database. 2017. http://dx.doi.org/10.5524/100362

Abbreviations

BLAST, basic local alignment search tool; bp, base pairs; chr., chromosome; DE, differential expression; dhp, days post hatched; FC, fold change; LG, linkage group; mya, million years ago; Mb, mega base; kb, kilo base; NCBI, National Centre for Biotechnology Information; nr, non-redundant; pg, pictograms; RH, radiation hybrid.

Acknowledgments

This project has received funding from the Greek Ministry of Education in the frame of the NSRF 2007-2013 Program (Project MBBC, Development Proposals from Research Institutions - KRIPIS) as well as from the European Union Horizon 2020 Research and Innovation Program European Marine Biological Research Infrastructure Cluster (EMBRIC) under grant agreement No. 654008.

Availability of data and materials

Datasets supporting the results of this article are available in the GigaDB (GigaDB, RRID:SCR_004002) repository associated with this publication [70]. All datasets were submitted to the public databases of the International Nucleotide Sequence Database Collaboration (INSDC), provided by DDBJ, EMBL-EBI and NCBI. All data and metadata were submitted under the Bioproject number PRJNA384295. Raw data are available from the SRA database under the accession number SRP105319.

Author contributions

E.S. participated in designing of the study, performed NGS meta analysis, comparative mapping analysis and conceived and wrote the main manuscript text. **A.Y.M.S**. carried out the transcriptome and genome assembly and generated the differential expression matrices. **E.K.**.performed RNA extraction, RNA library preparation and MiSeq sequencing. **G.D.G**. performed genome library preparation and Illumina sequencing, **N.P**. contributed to writing

and interpretation of the data, carried out muscle sampling of slow and fast/normal growing fish. **C.C M.** contributed to writing and interpretation of the data, conceived gonad and blood sampling. **G.K**. participated in designing of the study and contributed to writing and interpretation of the data. **A.M.** coordinated and designed the study as well as contributed to writing. All authors reviewed and approved the manuscript.

Figure 1: Image of the greater amberjack (Seriola dumerilii)

Click here to download Figure Figure 1-300.jpg

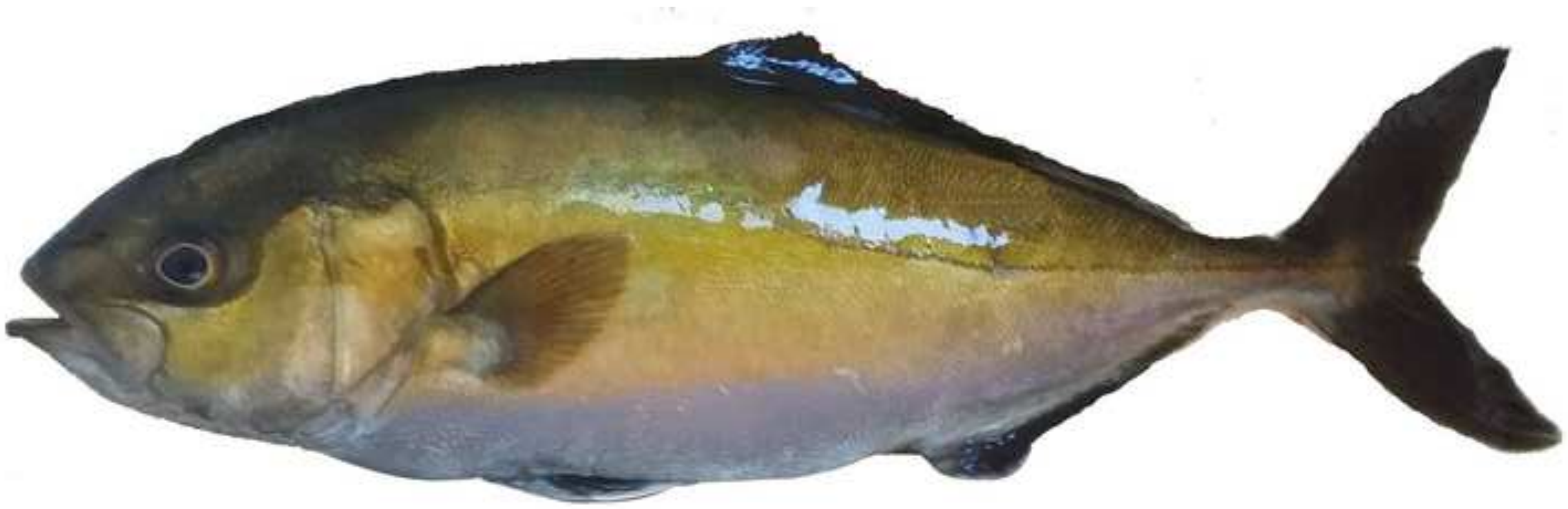

Figure 3: Identification by synteny of a putative amberjack sex-determination in silico linkage group.

Click here to download Figure Figure3.pptx

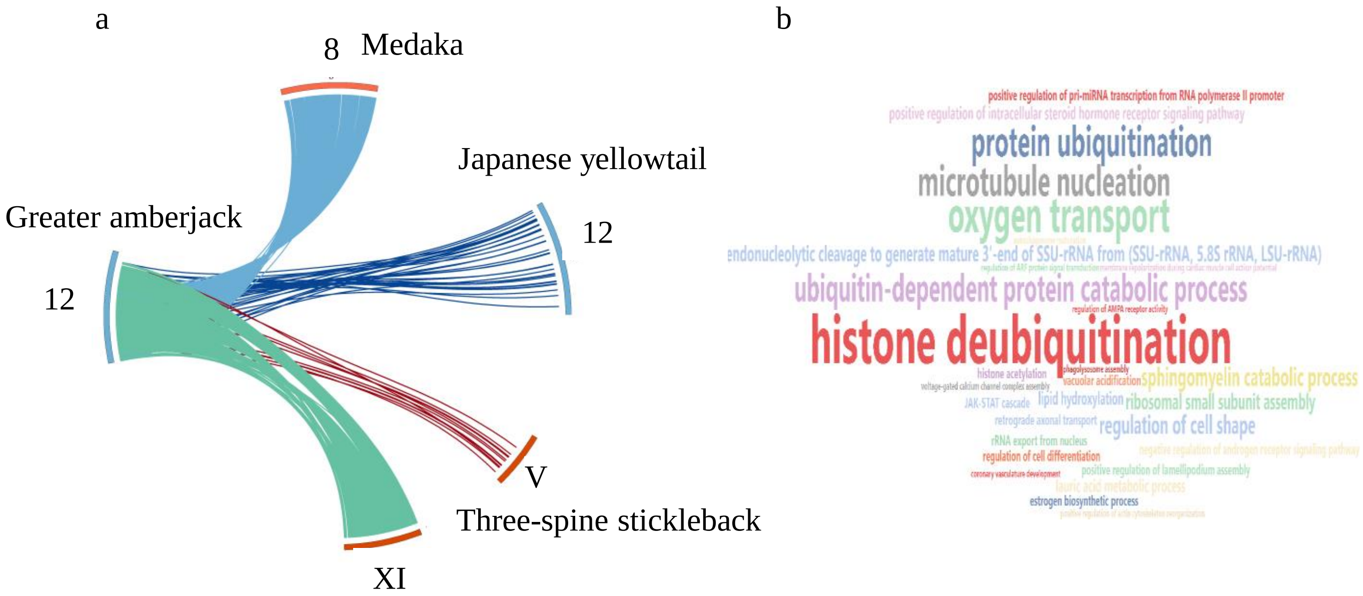

Figure 4: Overview of transcriptome study of female vs. male gonads.

Click here to download Figure Figure4.pptx

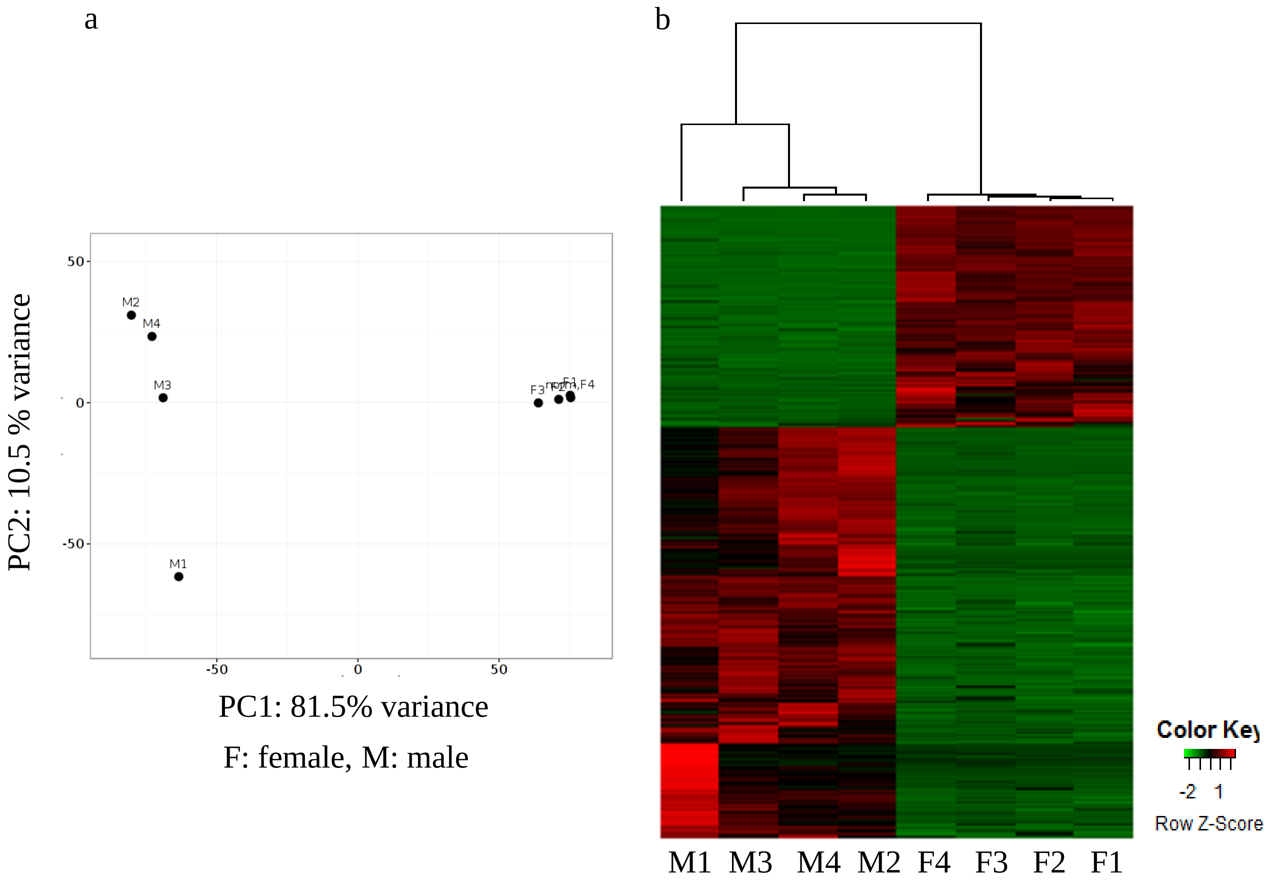

Figure 5: Word cloud illustration of significant enriched GO terms of the category Biological Process.

Click here to download Figure Figure5.pptx

Enrichment analysis: GO terms of molecular function are shown

a
up in male

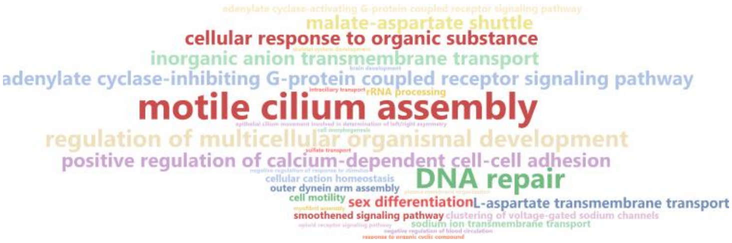

b
down in male

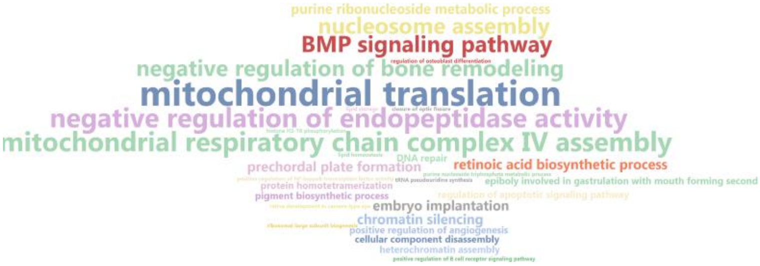

Figure 6: Overview of transcriptome study of slow growing vs. fast/normal-growing amberjacks.

Click here to download Figure Figure6.pptx

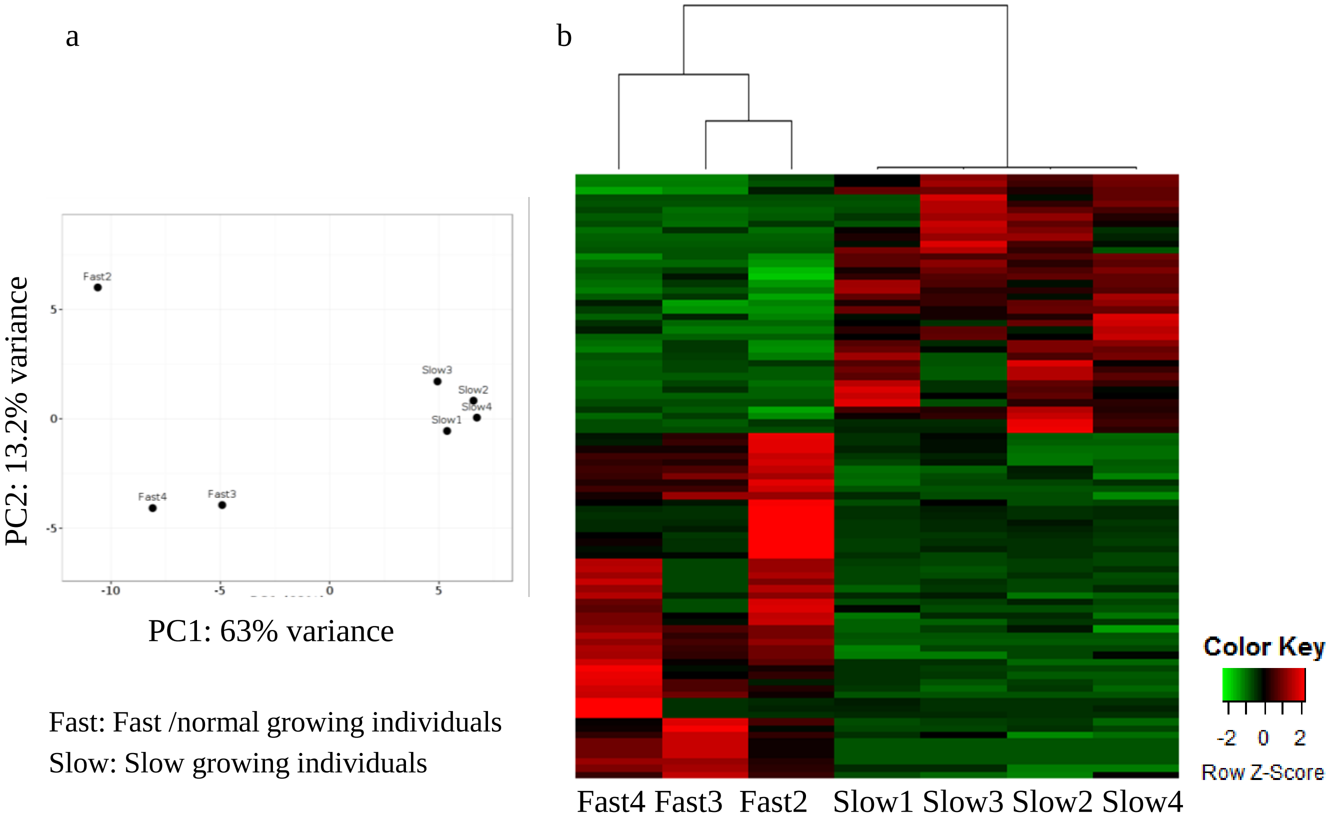

Figure 7: Word cloud illustration of significant enriched GO terms.

Click here to download Figure Figure7.pptx

a

all GO categories

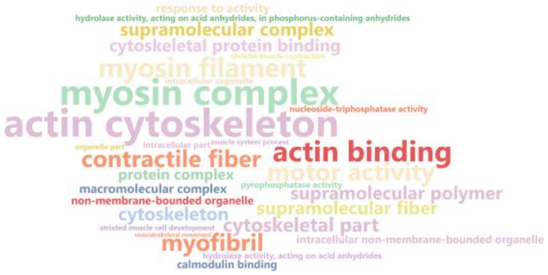

GO category: Biological Process

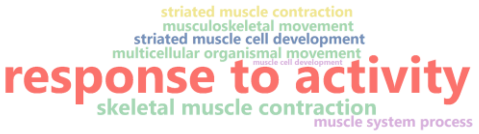

b

all GO categories

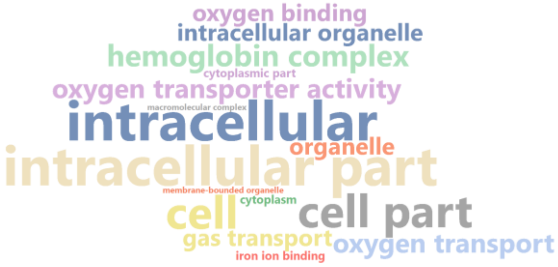

GO category: Biological Process

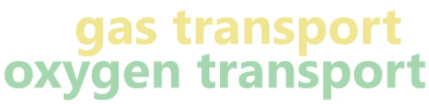

Figure 8: Gene expression displayed as heatmaps.

Click here to download Figure Figure8.pptx

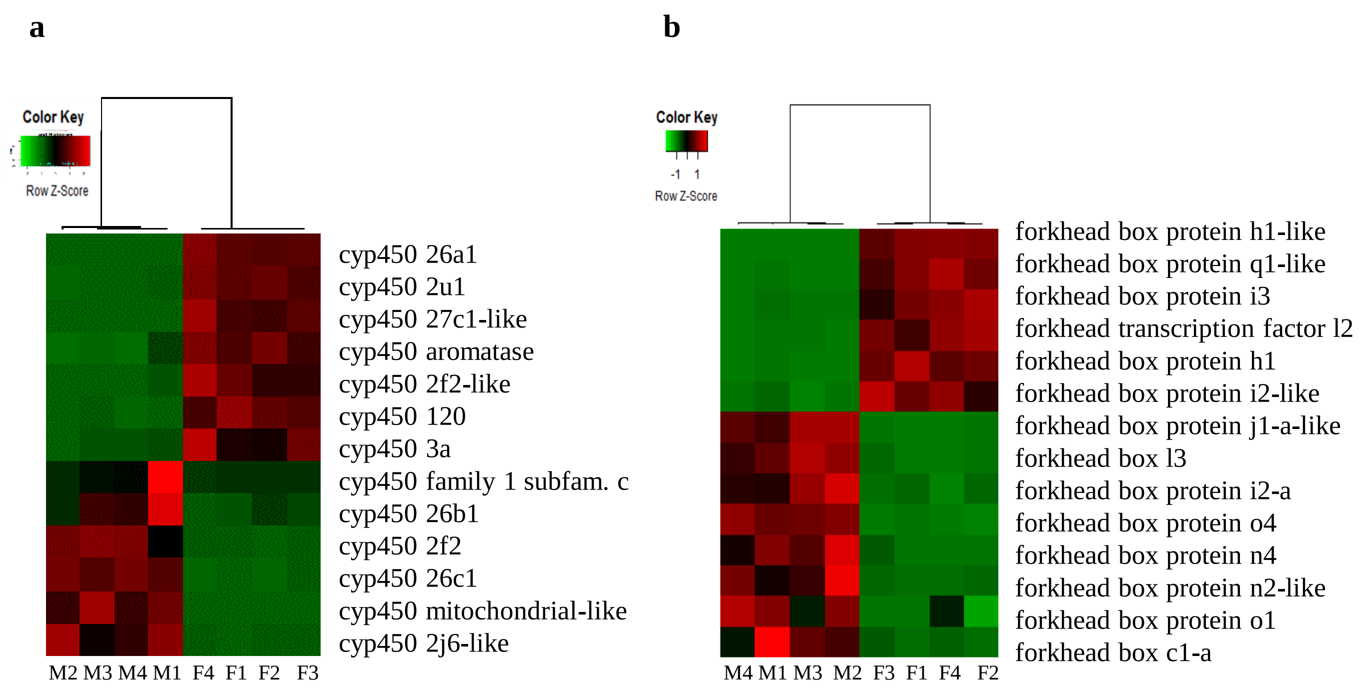

Figure 2: Overview of comparative mapping approach. Click here to download Figure Figure2.pptx

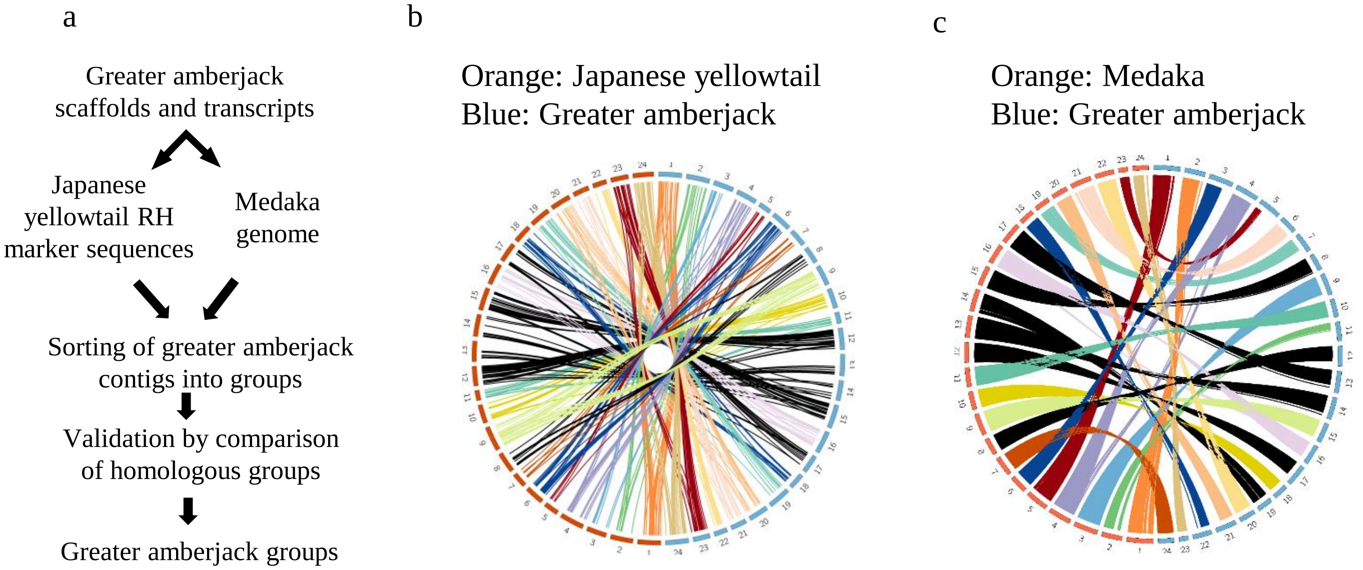

Additional file 3: Illustration in form of word cloud.

Click here to access/download
Supplementary Material
Additional file-3.docx

Additional file 4: RNA sequencing of female and male gonads.

Click here to access/download
Supplementary Material
Additional file-4.pptx

Additional file 7: RNA sequencing of fast /normal vs slow-growing individuals.

Click here to access/download
Supplementary Material
Additional file-7.pptx

Additional file 8: Illustration of comparative mapping approach.

Click here to access/download
Supplementary Material
Additional file-8.pptx

Additional file 9: Workflow overview.

Click here to access/download
Supplementary Material
Additional file-9.pptx

Additional file 1: K-mer profile plot using GenomeScope.

Click here to access/download
Supplementary Material
Additional file-1.pptx

Additional file 2: Annotated transcripts mapped onto the in silico group 12 of greater amberjack along with their expression values

Click here to access/download
Supplementary Material
Additional file-2.xlsx

Additional file 5: Count file of individual data values showing transcripts significantly higher expressed in female gonads and in

Click here to access/download
Supplementary Material
Additional file-5.xlsx

Additional file 6: Transcripts significantly higher expressed in female gonads and in male gonads along with their fold change

Click here to access/download
Supplementary Material
Additional file-6.xlsx
