# Supplementary material for: Full genome survey and dynamics of gene expression in the greater amberjack Seriola dumerili
Source: Gigascience. 2017 Nov 8;6(12):1–13. doi: 10.1093/gigascience/gix108 (PMC5751066; doi:10.1093/gigascience/gix108)
Supplement: Additional Files [file gix108_supp.zip › Additional file-7.pptx]

## Slide 1
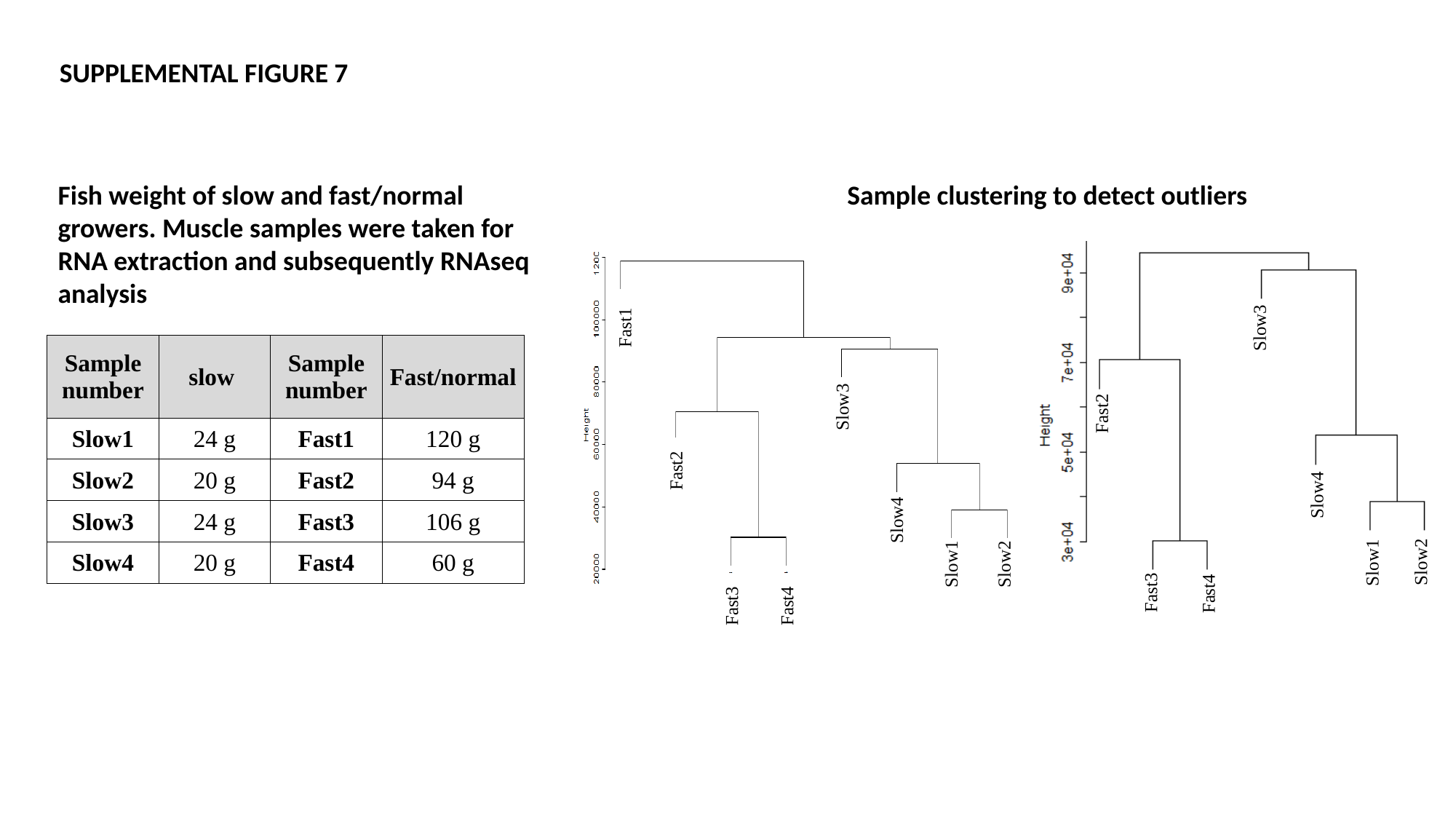

SUPPLEMENTAL FIGURE 7
Fish weight of slow and fast/normal growers. Muscle samples were taken for RNA extraction and subsequently RNAseq analysis
Sample clustering to detect outliers
Fast1
Slow3
| Sample number | slow | Sample number | Fast/normal |
| --- | --- | --- | --- |
| Slow1 | 24 g | Fast1 | 120 g |
| Slow2 | 20 g | Fast2 | 94 g |
| Slow3 | 24 g | Fast3 | 106 g |
| Slow4 | 20 g | Fast4 | 60 g |
Slow3
Fast2
Fast2
Slow4
Slow4
Slow2
Slow1
Slow1
Slow2
Fast3
Fast4
Fast3
Fast4
