# Supplementary material for: Full genome survey and dynamics of gene expression in the greater amberjack Seriola dumerili
Source: Gigascience. 2017 Nov 8;6(12):1–13. doi: 10.1093/gigascience/gix108 (PMC5751066; doi:10.1093/gigascience/gix108)
Supplement: Additional Files [file gix108_supp.zip › Additional file-8.pptx]

## Slide 1
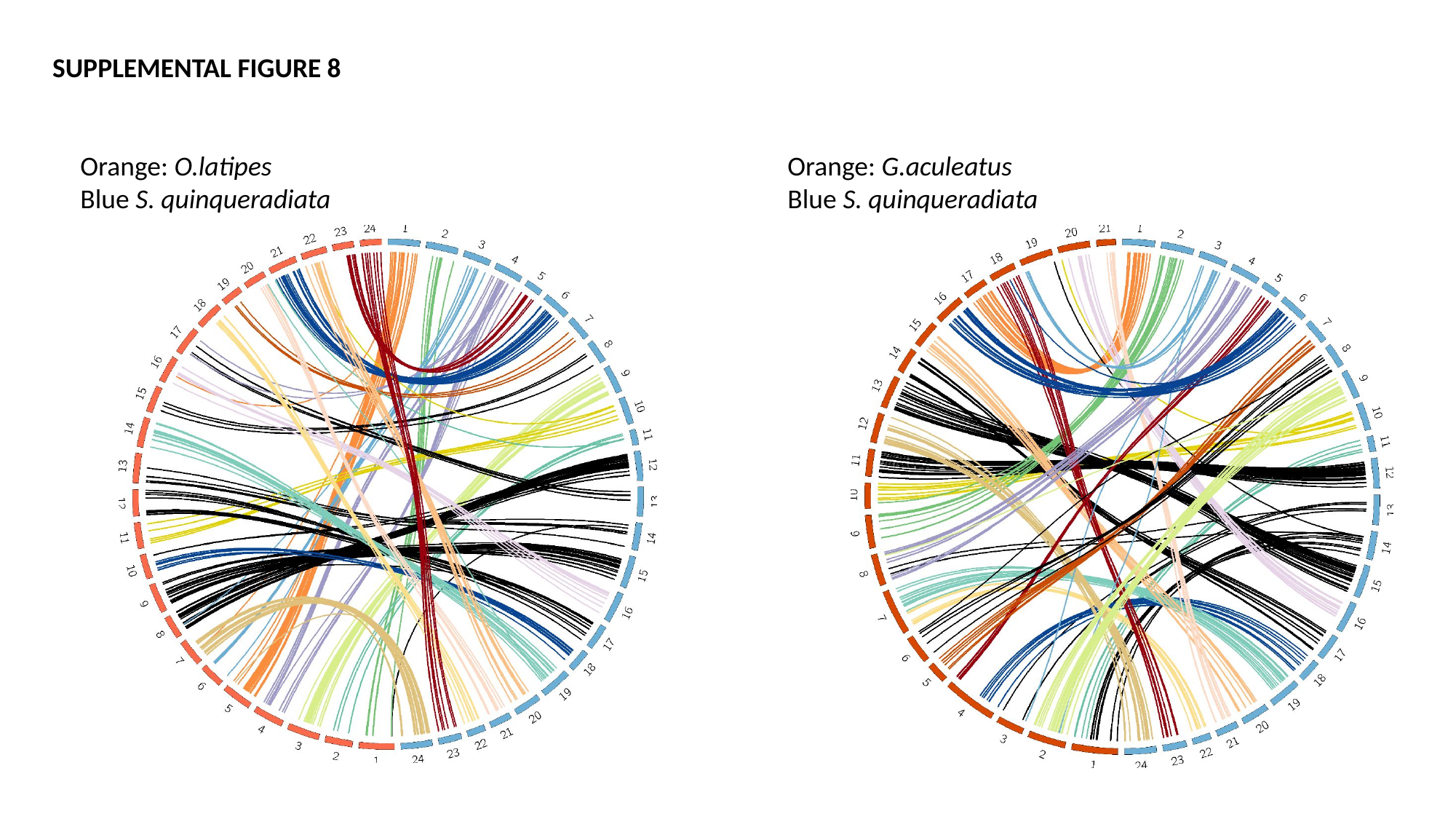

SUPPLEMENTAL FIGURE 8
Orange: O.latipes
Blue S. quinqueradiata
Orange: G.aculeatus
Blue S. quinqueradiata
