# Supplementary material for: Full genome survey and dynamics of gene expression in the greater amberjack Seriola dumerili
Source: Gigascience. 2017 Nov 8;6(12):1–13. doi: 10.1093/gigascience/gix108 (PMC5751066; doi:10.1093/gigascience/gix108)
Supplement: Additional Files [file gix108_supp.zip › Additional file-9.pptx]

## Slide 1
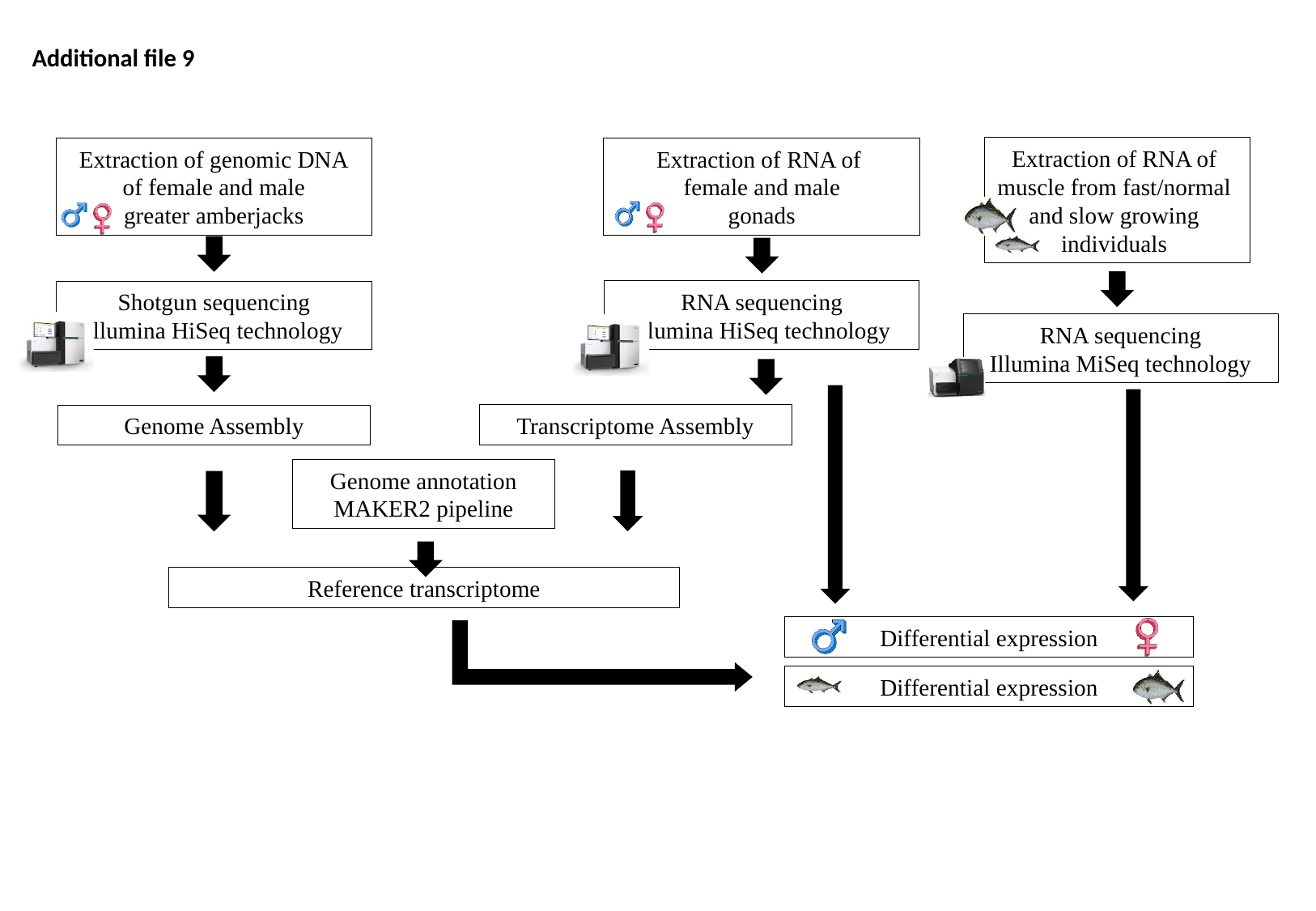

Additional file 9
Extraction of RNA of
muscle from fast/normal
and slow growing
individuals
Extraction of genomic DNA
of female and male
greater amberjacks
Extraction of RNA of
female and male
gonads
RNA sequencing
Illumina HiSeq technology
Shotgun sequencing
Illumina HiSeq technology
RNA sequencing
Illumina MiSeq technology
Genome Assembly
Transcriptome Assembly
Genome annotation MAKER2 pipeline
Reference transcriptome
Differential expression
Differential expression
