# Supplementary figures and images for: Full genome survey and dynamics of gene expression in the greater amberjack Seriola dumerili
Source: Gigascience. 2017 Nov 8;6(12):1–13. doi: 10.1093/gigascience/gix108 (PMC5751066; doi:10.1093/gigascience/gix108)

## Slide 1
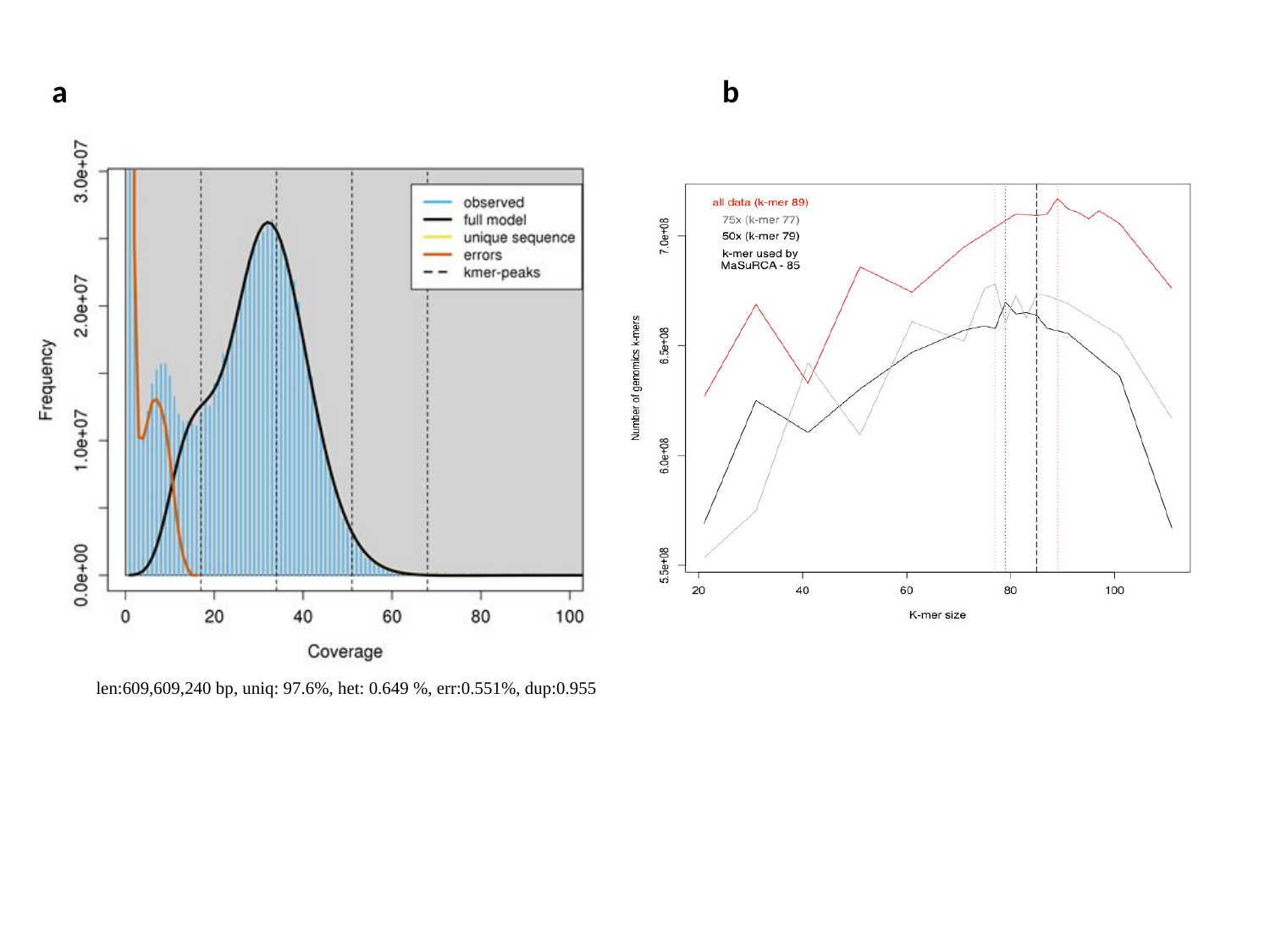

a
b
len:609,609,240 bp, uniq: 97.6%, het: 0.649 %, err:0.551%, dup:0.955

Supplement: Additional Files [file gix108_supp.zip › Additional file-1.pptx]

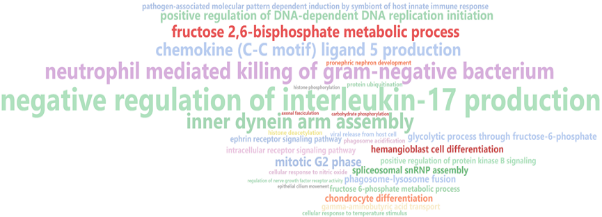


SD01

SD02


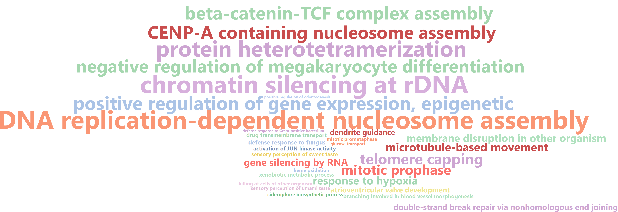


SD03

SD04


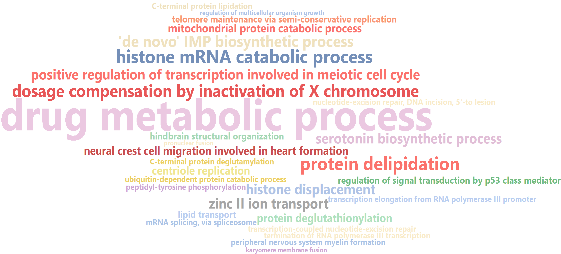

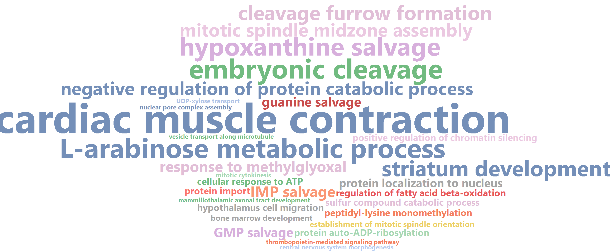


SD06

SD05


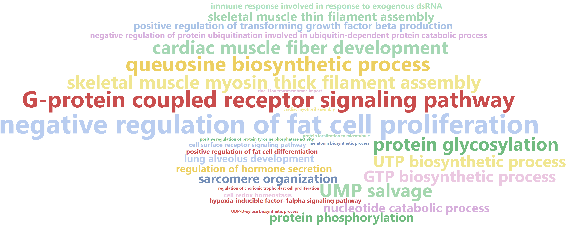

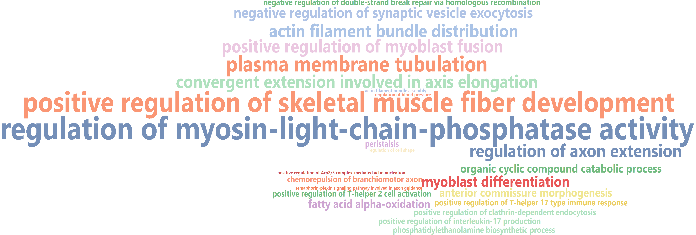


SD07

SD08


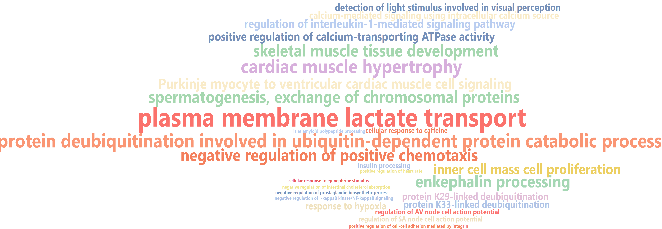

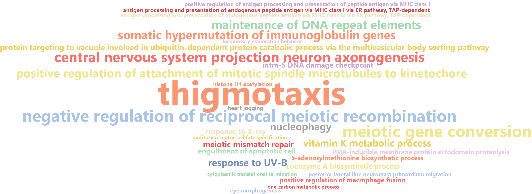


SD10

SD09


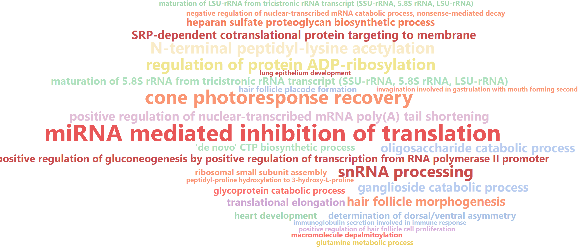

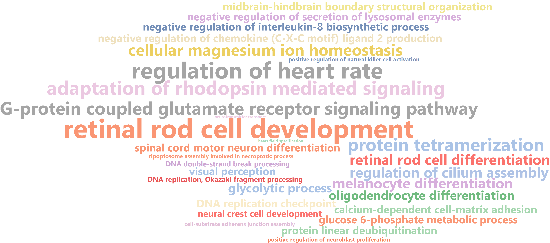


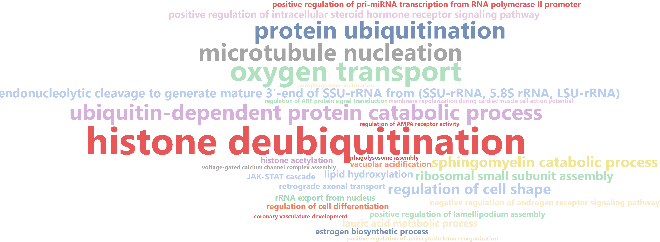

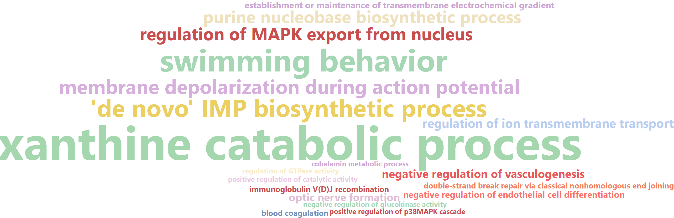


SD11

SD12


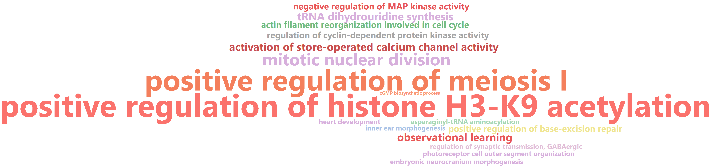

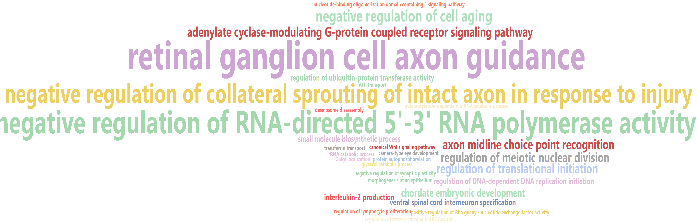


SD14

SD13

SD15


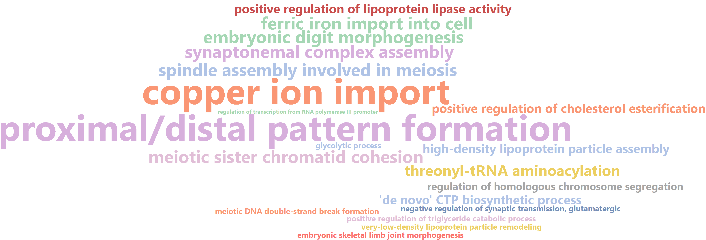

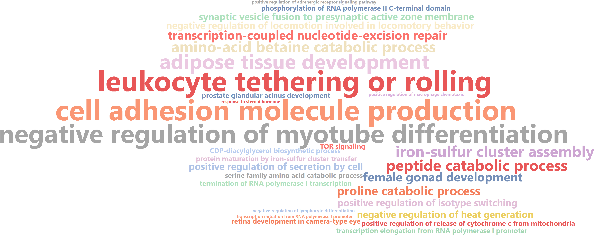


SD16


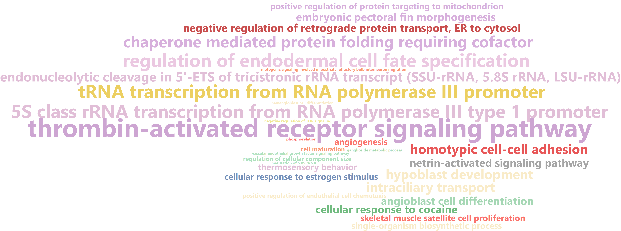


SD17

SD18


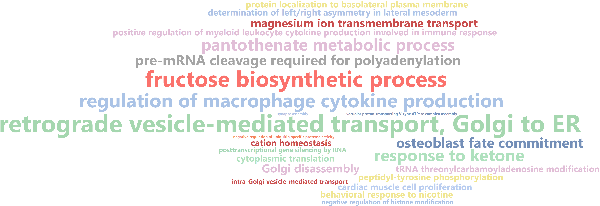


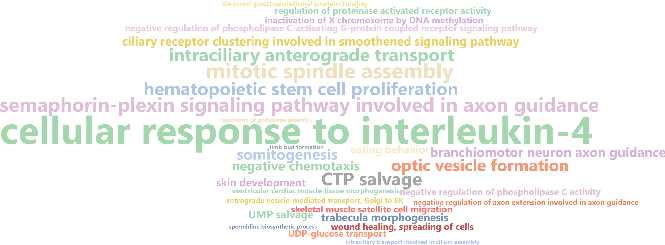

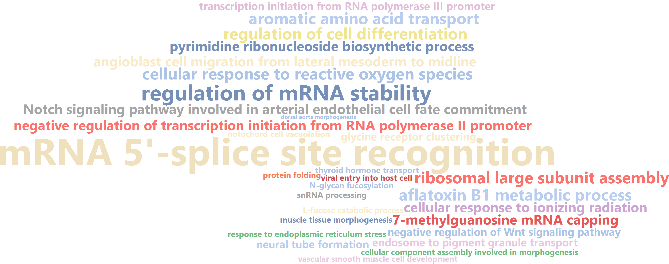

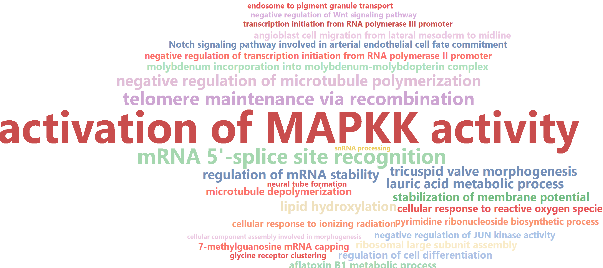

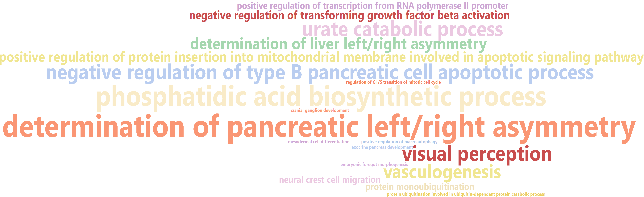

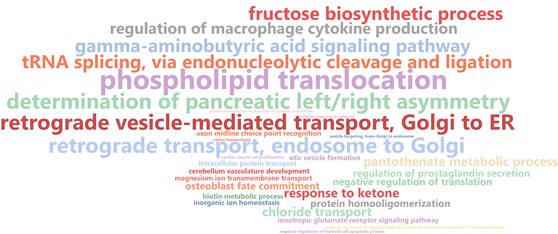

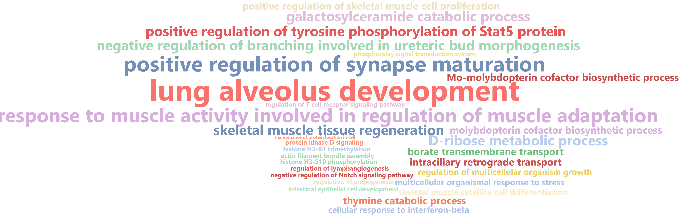


SD23

SD24

SD22

SD21

SD20

SD19

Supplement: Additional Files [file gix108_supp.zip › Additional file-3.docx]

## Slide 1
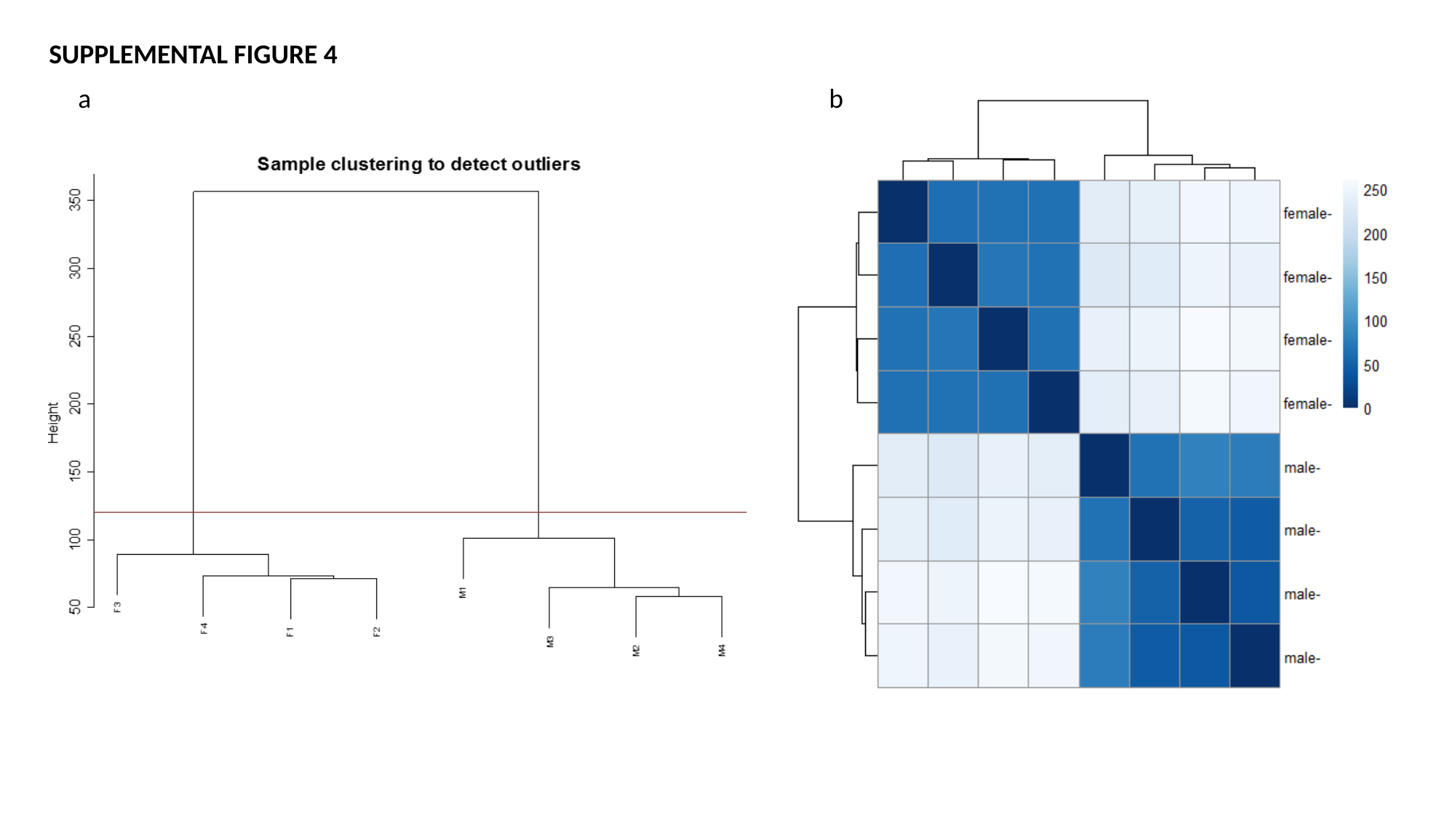

SUPPLEMENTAL FIGURE 4
a
b

Supplement: Additional Files [file gix108_supp.zip › Additional file-4.pptx]
