# Supplementary material for: Full genome survey and dynamics of gene expression in the greater amberjack Seriola dumerili
Source: Gigascience. 2017 Nov 8;6(12):1–13. doi: 10.1093/gigascience/gix108 (PMC5751066; doi:10.1093/gigascience/gix108)
Supplement: Response-to-Reviewer-Comments_Original-Submission.pdf [file gix108_response-to-reviewer-comments_original-submission.pdf]

Author's Response To Reviewer Comments

REVIEWER REPORTS:

AUTHORS WOULD LIKE TO THANK ALL THE REVIEWERS AND THE EDITOR FOR USEFUL COMMENTS ON OUR MANUSCRIPT.

REVIEWER #1:

1. Please add in references to all Supplementary files in main body of text. I was only able to find two references (supp 1 +4) in the main body of the text.
Please add a descriptive paragraph for each supplementary file that you provide.
Please add summary statistics on the genome annotation output from Maker. How many genes were identified, isoforms etc.

R: ALL SUPPLEMENTARY FILES WERE PRESENTED IN THE ORIGINAL MAIN BODY OF THE TEXT INDICATED AS "ADDITIONAL FILE 1, ADDITIONAL FILE 2 ETC." DESCRIPTION OF THE SUPPLEMENTARY FILES WERE ALSO GIVEN AFTER THE FIGURE LEGENDS. REVIEWER IS KINDLY ASKED TO CONFIRM THIS.

SUMMARY STATISTICS ON THE GENOME ANNOTATION HAS BEEN ADDED AND READS NOW:
"MAKER ANALYSIS PREDICTED 108,524 GENES AND 116,045 TRANSCRIPTS IN THE GENOME AND AFTER APPLYING THE RECOMMENDED THRESHOLD OF AED (ANNOTATION EDIT DISTANCE) < 1 RESULTED IN 45,547 AND 53,023 HIGH QUALITY GENES AND TRANSCRIPTS, RESPECTIVELY."
2. Line 396:
2.1. Please use BUSCO instead of CEGMA for determining quality of this assembly. CEGMA is now outdated as stated by authors. Please also report the results of your quality check. How many genes are missing, fragmented etc?

R: BUSCO WAS USED AS RECOMMENDED BY THE REVIEWER FOR DETERMINING QUALITY OF THE ASSEMBLY AND RESULTS ARE SHOWN BELOW AND PRESENTED IN THE RESULT SECTION.

"BUSCO WAS USED TO EVALUATE THE ASSEMBLED GENOME AND THE TRANSCRIPTOME AND OUT OF 303 BUSCO GROUPS (AKA CONSERVED ORTHOLOGS), SPECIFIC TO EUKARYOTES MORE THAN 93% WERE IDENTIFIED TO BE ENCODED BY BOTH GENOME AND THE TRANSCRIPTOME ASSEMBLY (TABLE 1)."

2.2. Please include how close is the zona pellucida sperm-binding protein 4 gene you identify on Linkage group 12 to the Japanese marker that identifies the sex locus in S. quinqueradiata. It would be nice to see this explored more to build up more of a
story to the article.

R: THE MARKER IDENTIFIED TO BE LINKED TO SEX IN S. QUINQUERADIATA ACCORDING TO KOYAMA ET AL. 2015 SSR263G21 WHICH HAS BEEN IDENTIFIED TO CODE FOR V-TYPE PROTON ATPASE 116 KDA SUBUNIT A ISOFORM 1 ISOFORM X. THIS GENE HAS ALSO BEEN FOUND IN GREATER AMBERJACK AND WAS SUCCESSFULLY MAPPED TO IN SILICO GROUP 12. THE DISTANCE TO ZONA PELLUCIDA SPERM-BINDING PROTEIN 4 GENE CAN ONLY BE DETERMINED ON THE GENOMES OF MEDAKA OR STICKLEBACK AS THOSE GENES ARE LOCATED ON DIFFERENT SCAFFOLDS. ADDITIONAL FILE-2 (PREVIOUS ADDITIONAL FILE 1) HAS BEEN CHANGED IN ORDER TO PROVIDE THIS INFORMATION NOW.

3. Please describe developmental differences observed in the 20 dph and 40dph small vs large fish. In RNA-seq experiments related to growth rate, it can be difficult to separate out differences in size due to genetics vs differences in developmental stage. In your experimental design it is possible that these two factors are confounded and it should be stated as such or not.

R: IN THE PRESENT STUDY INDIVIDUALS WERE TAKEN AT THE AGE OF 5 MONTHS, AS WRITTEN IN THE MATERIAL AND METHODS PART. CONSEQUENTLY THERE IS NO ISSUE DUE TO THE DEVELOPMENTAL STAGE. THE TWO GROWTH PERIODS WERE MENTIONED IN THE INTRODUCTION IN ORDER TO DESCRIBE THE BIOLOGY OF GREATER AMBERJACK GROWTH. THIS SENTENCE HAS NOW BEEN REMOVED IN ORDER NOT TO MISLEAD THE READER.

4. The biggest revision I have is that the authors are using the gene naming schema output from maker. A typical gene model schema for Seriola dumerili would be Sedum.G00000001 for Genes and Sedum.T00000001.1 Sedum.T00000001.2 etc for transcript isoforms of the genes. Renaming the gene models is important as other researchers will start using your gene model annotations and referring to them by name. Ideally, the authors will rename all the figures and tables with the new names. However, at a bare minimum the GFF file should be updated with new names and a 2 column reference file associating the maker names with the new naming schema supplied.

Please upload the renamed GFF file to GIGA Science.

R: UPDATED GFF FILES WITH NEW GENE MODEL SCHEME HAS BEEN UPLOADED. WE HAVE MODIFIED THE GENE AND TRANSCRIPT NAMES AS SUGGESTED BY THE REVIEWER. PLEASE FIND BELOW A SAMPLE OF THE REVISED NAMES.
MAKER2_TRANSCRIPTS.FASTA – UPDATED TRANSCRIPT FILE WITH FASTA HEADER AS BELOW
>SEDUM.T00000001.1 GENEID=SEDUM.G00000001 MAKER2_ID=MAKER-JCF7180000933113-SNAP-GENE-0.77-MRNA-1
TTCCTCTCTCCTACAGAAGCTCCTGGACAGGACTTTGTGACACTGGACTACCGTCTGCGC
MAKER2_PROTEINS.FASTA – UPDATED PROTEIN FILE WITH FASTA HEADER AS BELOW
>SEDUM.T00000001.1 GENEID=SEDUM.G00000001 MAKER2_ID=MAKER-

JCF7180000933113-SNAP-GENE-0.77-MRNA-1
FLSPTEAPGQDFVTLDYRLRYYPSITYAVIGSAVIFVLVVALLALVLHHQRKRSVLLPRG

REVIEWER #2:

General Comments:

Lines 147-149: "Out of the 53,023 obtained transcripts 44,371 were successfully mapped to the generated in silico groups of the greater amberjack and 30,342 transcripts to the genome of medaka." Do the authors have any indication as to why more than 20,000 predicted gene transcripts were identified and mapped to the generated in silico groups compared to the medaka genome? This (i.e., 53,000 gene transcripts) is substantially more than would be predicted for a typical diploid teleost genome (about 30,000). Splice variation or other reasons such as gene duplications?

R: IN THE PRESENT WORK THE OBTAINED TRANSCRIPTS WERE MAPPED AND NOT THE GENES, CONSEQUENTLY THE NUMBER IS HIGHER AS DIFFERENT TRANSCRIPT VARIANTS ARE INCLUDED. CONCERNING THE RNASEQ DATA WE PREFERRED TO WORK AT TRANSCRIPT LEVEL RATHER THAN THE GENE LEVEL AS THE WHOLE GENOME OF THE GREATER AMBERJACK IS NOT YET KNOWN. WITHIN THE MANUSCRIPT WE DID NOT USE THE EXPRESSION “GENE TRANSCRIPTS” LIKE IN THE COMMENT OF THE REFEREE.

Line 232: What do the authors mean by "sex-linked chromosomes"? Genes/loci that are found on sex chromosomes are "sex-linked", however the chromosomes themselves are considered "sex chromosomes" or "sex determining chromosomes".

Please clarify.

R: AUTHOR AGREE WITH THE REVIEWER THAT THE EXPRESSION “SEX-LINKED CHROMOSOMES” IS MISLEADING. THIS EXPRESSION HAS BEEN CHANGED TO “SEX DETERMINING CHROMOSOMES”.

Lines 349-350: "Gonad samples of four female and four male amberjacks (4n) were received from fish maintained at an aquaculture facility in Salamina." What were the sizes and maturity status of the fish? (i.e., sexually mature, and reproductive state, etc.). The "reproductive season (May)" is indicated, but were the males spermiating and females in post-vitellogenesis/ovarian maturation/ovulated?

R: HAS NOW BEEN REPHRASED AND READS “GONAD SAMPLES OF FOUR MATURE FEMALE AND FOUR MALE AMBERJACKS (N = 4 FISH) WERE RECEIVED FROM FISH MAINTAINED AT AN AQUACULTURE FACILITY IN SALAMINA (ARGOSARONIKOS FISHFARMING S.A., SALAMINA, GREECE) DURING THE PEAK OF THE REPRODUCTIVE SEASON (END OF MAY EARLY JUNE). AT THE TIME OF SAMPLING, FISH WERE 4 YEARS OLD AND HAD A BODY SIZE RANGING BETWEEN 9 AND 17 KG (ZUPA ET AL., 2016). THE FEMALES WERE IN ADVANCED VITELLOGENESIS,

WHILE THE MALES WERE EITHER IN ACTIVE SPERMATOGENESIS OR CONTAINED LUMINAL SPERMATOZOA WITH ONLY LIMITED DEVELOPING SPERMATOCYSTS."

Lines 252-253: "Thus, the present finding may indicate the importance of ubiquitination during sex determination." The role of the ubiquitin path also is related to control of the cell cycle (expression and degradation of regulatory proteins such as cyclins), including meiosis and germ cell proliferation and differentiation for gametogenesis. Especially when considering Figure 2, where "Histone Deubiquitination", "Protein Ubiquitination", and "Ubiquitin-dependent protein catabolic process" are considered enriched processes on putative sex chromosomes. Were any such gene transcripts identified in the gonad transcriptomes?
See below references and citations within:

Chapman, R.W., Reading, B.J., and Sullivan, C.V. 2014. Ovary transcriptome profiling via artificial intelligence reveals a transcriptomic fingerprint predicting egg quality in striped bass, Morone saxatilis. PLoS ONE 9(5):e96818.

Reading, B.J., Chapman, R.W., Schaff, J.E., Scholl, E.H., Opperman, C.H., and Sullivan, C.V. 2012. An ovary transcriptome for all maturational stages of the striped bass (Morone saxatilis), a highly advanced perciform fish. BMC Research Notes 5:111 (doi: 10.1186/1756-0500-5-111).

Also, "evidence points to the important role of ubiquitination in the regulation of spermatogenesis from the very beginning up to the spermatid differentiation". The same applies to oogenesis (from primary growth through ovulation and also directing early embryonic development; see citations above).

R: THE REQUESTED CITATIONS HAVE BEEN ADDED TO THE REFERENCE LIST. AUTHORS WOULD LIKE TO NOTE HERE, THAT THIS ANALYSIS DOES NOT REFER TO THE TRANSCRIPTOME ANALYSIS PERFORMED IN THE PRESENT STUDY, BUT TO THE TRANSCRIPTS WHICH WERE SUCCESSFULLY MAPPED TO THE GREATER AMBERJACK GENOME IN SILICO GROUP 12. IN COMPARISON TO THE WORK PERFORMED IN THE JAPANESE YELLOWTAIL WHERE AUTHORS DETERMINED THE LG12 (HOMOLOGOUS TO THE IN SILICO GROUP 12 OF THE GREATER AMBERJACK) AS THE SEX-DETERMINING LINKAGE GROUP.
ALL TRANSCRIPTS SIGNIFICANTLY HIGER EXPRESSED IN FEMALE GONADS AND IN MALE GONADS WITH DEG THRESHOLD PADJ<0.005 |LOG2FC| >2 ALONG WITH THEIR PUTATIVE ANNOTATIONS ARE GIVEN IN ADDITIONAL FILE 5 (FORMER ADDITIONAL FILE 4).

Line 345-346: "EDTA containing tubes". What concentration (mM) of EDTA was used as an anticoagulant?

R: BD VACUTAINER® PLASTIC K3EDTA BLOOD COLLECTION TUBES (REFERENCE NUMBER 368857, BD, FRANKLIN LAKES, NJ, USA) WERE USED FOR BLOOD SAMPLING. THIS INFORMATION HAS NOW BEEN ADDED.

Specific Comments:

Line 88: Plural/singular agreement. "sex determining mechanism" should be "sex determining mechanisms" or "sex determination mechanisms".

R: DONE. THANK YOU.

Line 92: Plural/singular agreement. "highly targeted research field" should be "highly targeted research fields".

R: DONE. THANK YOU.

Line 95: The word "been" can be omitted from the sentence "has been proved to be" ("has proved to be").

R: DONE. THANK YOU.

Line 138: "NCBI nr" the authors may consider stating the definition of "nr" as "NCBI non-redundant".

R: DONE. THANK YOU.

Line 165: The authors may consider changing "as differential expressed resulting" to "to be differentially expressed, resulting".

R: DONE. THANK YOU.

Line 166: The authors may consider changing "illustrated in form of a" to "illustrated in the form of a".

R: DONE. THANK YOU.

Line 167: Plural/singular agreement. "gonads gene expression" should be "gonad gene expression".

R: DONE. THANK YOU.

Line 221: The authors may consider revising the sentence "determined the number of chromosomes in the greater amberjack to 24" to "determined the haploid number of chromosomes in the greater amberjack to be 24".

R: DONE. THANK YOU.

Lines 230-231: The sentence "The homologous to the greater amberjack in silico group12 in medaka were found to be Chr.8 and in three-spine stickleback Chr.V and XI" isawkward. Do the authors mean to state "The greater amberjack in silico group 12 was found to be homologous to

medaka Chr.8 and three-spine stickleback Chr.V and XI"?

R: THE SENTENCE WAS RE-PHRASED. THANK YOU.

Line 246: "Enrichment analysis of transcripts successfully in the present study" is unclear ("successfully" what?).

R: WORD ORDER NOW CORRECTED. THANK YOU.

Line 295: "hypothesis that RA signaling pathway" should be "hypothesis that the RA signaling pathway".

R: DONE. THANK YOU.

Line 306: "their processes" should be "the processes".

R: DONE. THANK YOU.

Line 344: Should "HCMR" be spelled out at first use?

R: DONE. THANK YOU.

Lines 346-349: The authors might consider defining "n" here as "fish" in relation to biological sample size (e.g., n = 4 fish).

R: DONE. THANK YOU.

Table 4: Approved gene names should be in italic font.

R: GENE NAMES AND SYMBOLS WERE MODIFIED ACCORDING TO THE GUIDELINES FOR FORMATTING GENE AND PROTEIN NAMES (KATHERINE A., SCIENTIFIC WRITING, JANUARY 30, 2014) “MANY GENES ARE LISTED TOGETHER IN A TABLE, IT IS USUALLY UP TO THE AUTHORS’ (OR THE JOURNAL’S) DISCRETION AS TO WHETHER THEY SHOULD BE ITALICIZED. GENE NAMES THAT ARE WRITTEN OUT IN FULL ARE NOT ITALICIZED (E.G., INSULIN-LIKE GROWTH FACTOR 1).
THE SYMBOLS FOR GENES IN TABLE 4 ARE NOW ITALICIZED. THANK YOU.

REVIEWER #3:

There are several issues I would ask the authors to address:

1. The full assembly process is somewhat unconventional, and the manuscript could be improved with additional details and clarifications. Specifically:
1a. The 'in silico groups' (first introduced in the abstract, l. 58, and then in Results l. 145) are

hypothetical linkage groups/pseudochromosomes. It would be helpful if they are immediately introduced as such.

R: THE NAMING OF “IN SILICO GROUPS” WERE CHOSEN AS NO LINKAGE ANALYSIS HAS BEEN PERFORMED BUT GROUPS WERE IDENTIFIED USING COMPUTATIONAL MEANS (IN SILICO). CONSEQUENTLY THE TERM “LINKAGE” WOULD NOT BE APPROPRIATE HERE. ALSO THE TERM “PSEUDOCHROMOSOMES” DOES NOT SEEM TO US AS APPROPRIATE NEITHER, AS WE DID NOT IDENTIFY CHROMOSOMES. IN ADDITION IT SOUNDS LIKE “PSEUDO GENE” WHICH IS COMPLETELY MISLEADING.

1b. Why did you normalize the genomic reads (l. 133 and l. 394) using a transcriptome assembler? Is this standard practice for the MaSuRCA assembler? Doesn't it introduce artifacts?

R: DIGITAL NORMALIZATION HAS BEEN SHOWN TO DRAMATICALLY IMPROVE DE NOVO ASSEMBLY (FOR BOTH GENOMES AND TRANSCRIPTOMES) BY REDUCING THE COMPUTATIONAL REQUIREMENTS, WITHOUT SUBSTANTIALLY AFFECTING THE ASSEMBLY RESULTS. DETAILED REVIEW OF THIS METHOD CAN BE FOUND IN BROWN, ET.AL (2012, HTTPS://ARXIV.ORG/PDF/1203.4802.PDF). AS DESCRIBED IN THE REVIEW, THE TOOLS TRY TO REMOVE READS WITHOUT SIGNIFICANTLY AFFECTING THE TRUE K-MER CONTENT OF THE DATA SET AND TRIES TO ELIMINATE SEQUENCING ERRORS BY REMOVE SUCH READS WHICH MIGHT OTHERWISE INCREASE THE MEMORY/CPU REQUIREMENT SIGNIFICANTLY.
NORMALIZATION TO 50X WAS DECIDED ON TWO FACTORS:
1. REVIEW ON OPTIMUM SEQUENCING DEPTH REQUIRED FOR ASSEMBLIES WHICH SUGGESTS 50X AS DECENT BALANCE BETWEEN MEMORY REQUIREMENTS AND GENERATING A GOOD GENOME ASSEMBLY (HTTPS://DOI.ORG/10.1371/JOURNAL.PONE.0060204).
2. BASED ON TRIAL AND ERROR METHOD, WE FOUND THAT MASURCA WAS ABLE TO PRODUCE AN ASSEMBLY WITHIN A CONSIDERABLE TIME FRAME WITH 50X COVERAGE. WE DID TRY TO ASSEMBLE THE GENOME WITH THE 60X COVERAGE OR MORE AND THE ASSEMBLIES NEVER COMPLETED WITHOUT ERRORS.

TRINITY’S NORMALIZATION PROTOCOL WAS USED FOR DIGITAL NORMALIZATION DUE TO THREE KEY POINTS:
1. THE TOOL IS INDEPENDENT OF WHETHER THE DATA IS ORIGINATED FROM DNA OR RNA.
2. IT IS BASED ON K-MER SEARCH TOOL AS SUGGESTED BY THE ABOVE REVIEW.
3. EASY AND EFFICIENT TO USE.

1c. It is not immediately clear that the scaffolds and contigs in l. 135 are two different representations of the same assembly, i.e. there is no single dataset consisting of the combined 45909 scaffolds and 62353 contigs.

R: AUTHORS AGREE WITH THE REVIEWER'S COMMENT THAT GENOME ASSEMBLIES CANNOT CONSIST OF BOTH SCAFFOLDS 'AND' CONTIGS. THE LINE HAS BEEN MODIFIED AS BELOW TO REFLECT THE FACT THAT THE ASSEMBLER GENERATED SCAFFOLDS THAT CONSISTED OF CONTIGS AND GAPS.

"ASSEMBLY OF THE GENOME PRODUCED A 669,638,422 BP (~670 MB) GENOME REPRESENTED IN 45,909 SCAFFOLDS MADE UP OF 62,353 CONTIGS."

1d. The genome completeness analysis results (l. 396) should be included in the Results, and preferably performed using BUSCO instead of/in addition to CEGMA.

R: BUSCO WAS USED AS RECOMMENDED BY THE REVIEWER FOR DETERMINING QUALITY OF THE ASSEMBLY AND RESULTS ARE SHOWN BELOW AND PRESENTED IN THE RESULT SECTION.

"BUSCO WAS USED TO EVALUATE THE ASSEMBLED GENOME AND THE TRANSCRIPTOME AND OUT OF 303 BUSCO GROUPS (AKA CONSERVED ORTHOLOGS), SPECIFIC TO EUKARYOTES MORE THAN 93% WERE IDENTIFIED TO BE ENCODED BY BOTH GENOME AND THE TRANSCRIPTOME ASSEMBLY (TABLE 1)."

1e. According to l. 143, 14990 scaffolds were mapped to the medaka genome to construct the 'in silico groups'. The number suggests this is only a small fraction of the genome, but I assume these are primarily larger scaffolds. A more informative number would therefore be the percentage of the assembly that could be assigned to a group. (I believe it is ~80%, based on a sequence file provided for review but not listed in the manuscript).

R: VERY STRICT PARAMETERS AS NOTED WITHIN THE MANUSCRIPT WERE USED IN ORDER TO GENERATE THE IN SILICO GROUPS. PERCENTAGES OF SUCCESSFULLY MAPPED TRANSCRIPTS TO THE GENERATED IN SILICO GROUPS ARE NOW ADDED AT LINES 156-158 (RESULT SECTION).

1f. The Results suggest 'synteny analysis' (l. 145) is possible with the hypothetical groups. This is not really performed, except for figures 1b-c. In the end, the 'on-to-one relationship' between Seriola and medaka (Discussion l. 225-227) has some aspects of circular reasoning, as it is based on only 468 marker matches. Therefore, this alignment is mostly based on an indirect alignment of the medaka genome with itself. (Incidentally - the manuscript in different places refers to 'circus plots', which should be 'Circos plots'.)

R: SYNTENY ANALYSIS WAS PERFORMED USING THE GENERATED TRANSCRIPTS WHEREAS THE IN SILICO GROUPS WERE BASED ON THE SCAFFOLDS. THE 468 MARKERS MENTIONED BY THE REFEREE ARE CONCERNING THE PUBLIC AVAILABLE MARKERS OBTAINED FROM THE JAPANESE YELLOWTAIL. HOWEVER THE GENERATED IN SILICO GROUPS WERE NOT ONLY BASED ON THE HOMOLOGY SEARCH OF THE GREATER AMBERJACK WITH THE JAPANESE YELLOWTAIL BUT ALSO WITH THE MODEL FISH SPECIES MEDAKA (FIG.1) AND

STICKLEBACK (ADD.FILE 7). TRANSCRIPTS WERE MAPPED TO EACH FISH GENOME SEPARATELY. THE CORRECTNESS OF THE ASSIGNED IN SILICO GROUPS WERE EVALUATED BASED ON THE PUBLISHED SYNTENY ANALYSIS OF THE JAPANESE YELLOWTAIL AND MEDAKA. CONSEQUENTLY THERE CANNOT BE ANY ASPECTS OF CIRCULAR REASONING.

THE TYPO IS NOW CORRECTED, THANK YOU.

2. The gonadal transcriptome analysis is based on a (technically sound) RNA-seq comparison of testis with ovaries. However, I think the interpretation could be improved:
2a. These are completely different tissues. Therefore, 'upregulation' and 'downregulation' are not really suitable descriptions of gene expression level differences, as there is no developmental path leading from one condition to the other by tweaking expression levels. Therefore, 'higher/lower expression in' would be more appropriate.

R: THE AUTHORS AGREE WITH THE REFEREE AND THE DESCRIPTIONS FOR THE GONAD ANALYSIS HAVE BEEN CHANGED TO 'HIGHER/LOWER EXPRESSION' AS SUGGESTED.

2b. Related: when comparing different tissues, these differences are best interpreted qualitatively rather than quantitatively (and without statistical tests), as there is no reasonable way to align the profiles. In this study, standard DESeq2 normalization (l. 444) is presumably applied, but using different tissues almost certainly violates the assumptions behind its method (i.e. minimize gene expression differences). Its validity needs to be demonstrated for this data, for example by showing the overall similarity between the profiles and their successful normalization.

R: AUTHORS AGREE WITH THE DIFFERENT TERM FOR THE DESCRIPTION OF THE GONAD EXPRESSION RESULTS. IN ORDER TO OBTAIN HOWEVER GENDER SPECIFIC GENE EXPRESSION, THE PATTERNS HAVE TO BE COMPARED AND DIFFERENCES HAVE TO BE TESTED FOR SIGNIFICANCE. CONCERNING THE USAGE OF DESEQ2 IT HAS TO BE NOTED THAT THIS IS A STANDARD ANALYSIS METHOD USED IN MANY SIMILAR TRANSCRIPTOME ANALYSIS STUDIES, E.G. FAN ET AL.2014, LIU ET AL., 2015. VALIDATION OF THE DATA HAS BEEN PERFORMED EXTENSIVELY IN THE FORM OF PCA ANALYSIS, HIERARCHICAL CLUSTERING ANALYSIS AS WELL AS PRESENTING A HEATMAP SHOWING THE CONSISTENCY OF EXPRESSION AMONG BIOLOGICAL REPLICATES (FIG.3). IN ADDITION THE OVERALL SIMILARITY HAS ALSO BEEN EXAMINED BY CALCULATING THE SAMPLE-TO-SAMPLE DISTANCES (ADDITIONAL FILE 3).

2c. In general, this study examines sex-specific gene expression, but also discusses sex determination. Is there a reason to assume the genes highly 'differentially expressed' in gonads are the genes responsible for sex determination?

R: THE ASSUMPTION OF THE REFEREE WAS NOT DONE IN THE MANUSCRIPT. THE PRESENT WORK PRESENTS THE DRAFT GENOME OF THE GREATER AMBERJACK AND PERFORMS A COMPARATIVE MAPPING APPROACH. SEX DETERMINATION IS

DISCUSSED IN THIS PART OF THE PRESENT STUDY BASED ON THE FINDINGS IN THE JAPANESE YELLOW TAIL. HERE THE SEX DETERMINING LOCUS WAS IDENTIFIED IN LG12. THE TRANSCRIPTS IDENTIFIED WITHIN THE HOMOLOGOUS IN SILICO GROUP OF THE GREATER AMBERJACK WERE EXAMINED IN THE LIGHT OF SEX DETERMINATION.
THE STUDY OF GENES HIGHLY DIFFERENTIAL EXPRESSED IN MALE VS FEMALE GONADS REVEALS TRANSCRIPTS WITH DISTINCT ROLES BETWEEN THE TWO GENDERS. IN GENERAL, TELEOSTS COMPRISE A GREAT DIVERSITY OF SEX DETERMINING MECHANISMS, AND SEX DIFFERENTIATION INCLUDES THE EXPRESSION OF A SUBSTANTIAL NUMBER OF GENES IN A SPATIAL AND SEQUENTIAL ORDER. ADDITIONALLY, THE GENE IDENTIFIED AS SEX DETERMINING GENE IN THE ONE SPECIES MAYBE SEX SPECIFIC IN THE OTHER.

2d. Related: the genome assembly is based on two individuals, a male and a female. Unfortunately, the sequencing libraries have been pooled (l. 377), otherwise it would have been relatively straightforward to identify (small) loci not present in males (assuming ZW sex determination). It may still be possible to identify contigs with relatively low coverage - these could be candidate sex determination regions.

R: AUTHORS AGREE WITH THE REFEREE BUT AT THE PRESENT STAGE THIS WAS NOT POSSIBLE (HIGHER THROUGHPUT NECESSARY) AND WILL BE ADDRESSED IN A LATER STUDY.

2e. What is the difference between the differential expression analyses of lines 164-165 and lines 170-171?

R: THE REFEREE IS RIGHT HERE. THERE IS NO DIFFERENCE IN THE ANALYSIS. 164-165 AND LINES 170-171 ARE REDUNDANT THE FIRST NUMBER IN LINE 165 IS INCORRECT. THIS HAS NOW BEEN CORRECTED IN THE MANUSCRIPT.

3. Line 181: please mention that these transcriptomes are of muscle tissue (the current phrasing suggests complete animals have been profiled). For this part of the study, animals at the same age were used. I assume the slow- and fast-growing animals were at the same developmental stage here?

R: IT IS NOW ALSO INDICATED HERE THAT THE MUSCLE TRANSCRIPTOME WAS STUDIED. AS MENTIONED IN THE MATERIAL AND METHOD SECTION, INDIVIDUALS WERE TAKEN AT THE AGE OF 5 MONTHS.

4. The Discussion of the genome assembly quality in the light of other Mediterranean fish species is not entirely appropriate (lines 195-219). The species are not closely related. Although genome size and sequence coverage are factors influencing short-read assembly contiguity, genome structure (i.e. repeat landscape) and heterozygosity level are probably much more important. Both have not been analyzed. The Seriola assembly may well have ended up more fragmented than necessary by pooling the genomes of two individuals. This choice should be

discussed, and an analysis of overall heterozygosity (e.g. using a k-mer profile) would be relevant here.

R: AS SUGGESTED BY THE REVIEWER THE K-MER PROFILE AND HETEROZYGOSITY LEVELE HAS BEEN CALCULATED USING KMERGENIE V1.6982 (HTTP://KMERGENIE.BX.PSU.EDU/; HTTPS://DOI.ORG/10.1093/BIOINFORMATICS/BTT310) AS WELL AS GENOMESCOPE: FAST REFERENCE-FREE GENOME PROFILING. METHOD HAS BEEN ADDED TO THE MATERIAL AND METHOD SECTION AND RESULTS ARE PRESENT IN THE RESULT SECTION. PLOTS ARE PROVIDED IN SUPPLEMENTAL FIGURE 1.
