# Supplementary material for: Full genome survey and dynamics of gene expression in the greater amberjack Seriola dumerili
Source: Gigascience. 2017 Nov 8;6(12):1–13. doi: 10.1093/gigascience/gix108 (PMC5751066; doi:10.1093/gigascience/gix108)
Supplement: Response-to-Reviewer-Comments_Revision-1.pdf [file gix108_response-to-reviewer-comments_revision-1.pdf]

Author's Response To Reviewer Comments

POINT-BY-POINT RESPONSE

REVIEWER #1:

1) Thank you for updating your gene models.
Please update your supplementary tables to reflect the new gene models.

R: TABLES ARE NOW ALL UPDATED. THANK YOU FOR PINPOINTING TO THIS.

2) Thank you for running BUSCO, In the future consider running an orthologous gene set such as vertebrata_odb9 that is closer to your species to get a more accurate assessment of genome assembly.
R: THE COMMENT IS VERY MUCH APPRECIATED, THANK YOU. THE QUALITY WAS CHECKED IN THE PRESENT WORK USING EUKARYOTA_ODB9 AND ZEBRAFISH AS MENTION IN LINE 407, PAGE15.
3) Please be sure to release the SRA SRP105319 after publication, I was not able to find it and is likely be held until publication.

R: ALL THE SEQUENCE DATA SUBMITTED TO THE SRA DATABASE ARE HOLD UP UNTIL PUBLICATION. NEW SRA PUBLICATION DATE HAS BEEN SET TO 2017-11-30. IF PUBLICATION OCCURS EARLIER OF COURSE ALL DATA WILL BE PUBLISHED BEFORE THIS DATE.

4) You might consider taking out the two paragraphs (1 in results, 1 in discussion) about fast and slow growing fish and writing it up as a separate paper, it would tighten up the story and doesn't add a lot of value to this paper. (suggestion not a requirement).

R: WE WOULD LIKE TO THANK THE REVIEWER FOR SHARING HIS VIEWPOINT WITH US. IN CAPTIVITY GREATER AMBERJACK REPRODUCTION HAS PROVED TO BE CHALLENGING AND OBSERVED GROWTH DYSFUNCTION HINDERS THEIR DOMESTICATION. INSIGHTS INTO MOLECULAR MECHANISMS MAY CONTRIBUTE TO A BETTER UNDERSTANDING OF TRAITS LIKE GROWTH (AND SEX) BUT INVESTIGATIONS TO UNRAVEL THE MOLECULAR BACKGROUND OF AMBERJACKS HAVE BEGUN ONLY RECENTLY. THE PRESENT MANUSCRIPT IS THE FIRST TO ATTEMPT TO ASSESS THE MOLECULAR BACKGROUND / DIFFERENCES OF SLOW-GROWING VS. FAST/NORMAL GROWING GREATER AMBERJACKS AS WELL AS OF MALE AND FEMALE INDIVIDUALS. WE SHOW THAT SPECIFIC EXPRESSION PATTERNS ARE DETECTED BETWEEN SLOW AND FAST GROWING FISH WITH SLOW GROWING FISH COMPRISING ENRICHED TRANSCRIPTS INVOLVED IN OXYGEN TRANSPORT. TAKEN TOGETHER WE PREFER TO KEEP THIS PART WITHIN THE PRESENT STUDY AS WE BELIEVE THAT THE RESULTS OF FAST AND SLOW GROWING FISH ARE OF GREAT IMPORTANCE.

Reviewer #3:
I thank the authors for the answers they provided, and I am satisfied with most of the changes to the manuscript. For three issues, I would still like to suggest some minor clarification and/or context.

1. Please also revise the figure order/labels, fig 2 of the text is included as fig 8.

R: PLEASE ACCEPT OUR APOLOGIES HERE. APPARENTLY THE FIGURE WAS UPLOADED TWICE. THIS HAS NOW BEEN CORRECTED.

2. Suggestion 1 (previously 1f): The Seriola transcripts are mapped to the medaka genome in order to select genes putatively located at the sex-determining locus on chromosome 12. This is a technical sorting procedure, which is perfectly suitable. Figure 8c (2c) then illustrates the quality of this sorting procedure, as does figure 2a (3a). The use of the word 'synteny' in the text (l 154) and the use of Circos plots could suggest these are analyses of genome evolution, which is not studied. Perhaps 'synteny' could be avoided here?

R: INDEED THE ANALYSIS OF GENOME EVOLUTION WAS NOT WITHIN THE SCOPE OF THE PRESENT STUDY. THE TERM “SYNTENY” ACCORDING TO ©NATURE EDUCATION (WWW.NATURE.COM/SCITABLE/DEFINITION) IS A “TERM USED TO DESCRIBE THE STATE OF TWO OR MORE GENES BEING PRESENT IN THE SAME CHROMOSOME, THOUGH NOT NECESSARILY LINKED.” THE FAO CORPORATE DOCUMENT REPOSITORY (WWW.FAO.ORG) DEFINES THE TERM “SYNTENY” IN THE PUBLICATION ENTITLED WITH “GLOSSARY OF BIOTECHNOLOGY AND GENETIC ENGINEERING” AS “THE OCCURRENCE OF TWO OR MORE LOCI ON THE SAME CHROMOSOME, WITHOUT REGARD TO THE DISTANCE BETWEEN THEM. AN ALTERNATIVE TERM TO “SYNTENY” WOULD BE THE TERM “CO-LOCATE”. THIS TERM HOWEVER PINPOINTS TO A MORE DISTINCT LOCATION ON THE CHROMOSOME RATHER JUST THE PRESENCE OF GENES IN THE SAME CHROMOSOME. GIVEN THE DEFINITIONS ABOVE AS WELL AS THE FACT THAT THE PRESENT WORK REFERS TO THE WORK PREVIOUSLY PUBLISHED BY AOKI ET AL. IN BMC GENOMICS WHERE THE TERM WAS USED IN THE SAME MANNER AS IN THE PRESENT STUDY WE WOULD LIKE TO KEEP THE EXPRESSION “SYNTENY” HERE.

3. Suggestion 2 (previously 2b): I am aware of the ubiquitous use of DESeq2 and tools like it. That they are widely used does not mean that they are always used appropriately. I was getting at the issue of between-sample normalization, which is mostly automatic in these tools and assumes samples are comparable. In this case, I doubt the samples are comparable under the implicit assumptions of DESeq normalization. The illustrations in figure 3 are post-analysis, and do not address this concern. If anything, the heatmap and the skew towards higher expression in one tissue (line 178) imply that the samples are not well comparable. This issue is widespread in RNA-seq analysis, and I do not expect this paper to address it at a fundamental level; however, I would suggest caution in interpreting these differential expression results, with perhaps a caveat in the Results, and including more details on DESeq2 deployment in the Methods.

R: CERTAINLY EVERY TOOL HAS SOME DISADVANTAGES AND SEVERAL PAPERS TRY TO COMPARE DIFFERENT ANALYSIS TOOLS WITH EACH OTHER. THEREFORE IN THE PRESENT STUDY A STRINGENT THRESHOLD HAS BEEN CHOSEN AND CONCLUSIONS WERE FORMULATED WITH CAVEAT.
MORE DETAILS ON DESeq2 DEPLOYMENT HAVE BEEN ADDED.

4. Suggestion 3 (previously 2c): I concede that the assumption I made about the link between sex determination and sex-linked gene expression is not emphasized in the manuscript. My apologies for this. I do, however, think it is a natural question to ask, which other readers may do as well. Both issues are discussed immediately after each other, with the sentences linking them (l246-250) discussing a gene (GIPC1) that might be sex-determining, but is not differentially expressed, thus encouraging my interpretation of an implicit assumption. Perhaps you could clarify the difference between the two analyses for the readers, for instance by mentioning there that such a link between sex determination and sex-linked expression is neither necessary nor expected.

R: WE AGREE WITH THE REFEREE THAT CLARIFYING THE DIFFERENCE FACILITATES THE READER TO FOLLOW THE PRESENTED WORK. WE ADDED THE SUGGESTED SENTENCE IN LINE 267.

Details:
- l145 conversed - > conserved.

R: DONE THANK YOU!

- fig 3: one species is not labeled, I assume this is Tetraodon (based on the Methods)?

R: ALL SPECIES ARE LABELED. PLEASE HAVE A LOOK AT THE FIGURE LEGEND AS WELL AS LINE 161, PAGE 6.

- The k-mer analyses are useful. There is an additional bump (modeled as errors) before the heterozygous peak. Could this be the result of mixing genomic information from two individuals?

R: GENOMESCOPE ATTEMPS SEVERAL ROUNDS OF MODEL FITTING IN ORDER TO COME UP WITH THE FINAL ONE. THE PRESENTED PROFILE HERE REFLECTS, ACCORDING TO GENOMESCOPE A HOMOZYGOUS GENOME. HETEROZYGOUS ONES INSTEAD SHOULD SHOW A CHARACTERISTIC BIMODAL PROFILES. (KAJITANI ET AL. 2014). REFERENCE: VURTURE GW, SEDLAZECK FJ, NATTESTAD M, UNDERWOOD CJ, FANG H, GURTOWSKI J, ET AL. GENOMESCOPE: FAST REFERENCE-FREE GENOME PROFILING FROM SHORT READS. BIOINFORMATICS . 2017;1–3.
