# Supplementary material for: Full genome survey and dynamics of gene expression in the greater amberjack Seriola dumerili
Source: Gigascience. 2017 Nov 8;6(12):1–13. doi: 10.1093/gigascience/gix108 (PMC5751066; doi:10.1093/gigascience/gix108)
Supplement: Response-to-Reviewer-Comments_Revision-2.pdf [file gix108_response-to-reviewer-comments_revision-2.pdf]

Author's Response To Reviewer Comments

All comments of the reviewer are addressed and the manuscript has been transformed to the Data note format as requested by the editor following the instruction at https://academic.oup.com/gigascience/pages/data_note
