# Supplementary material for: Full genome survey and dynamics of gene expression in the greater amberjack Seriola dumerili
Source: Gigascience. 2017 Nov 8;6(12):1–13. doi: 10.1093/gigascience/gix108 (PMC5751066; doi:10.1093/gigascience/gix108)
Supplement: Reviewer-1-Report-(Original-Submission).pdf [file gix108_reviewer-1-report-(original-submission).pdf]

Reviewer Report

Title: Full Genome Survey and Dynamics of Gene Expression in the Greater Amberjack Seriola dumerili

Version: Original Submission **Date:** 7/6/2017

Reviewer name: Andrew Severin

Reviewer Comments to Author:

The authors describe the genome of Seriola dumerili a teolost fish and provide summary statistics and go enrichment of two RNA-Seq experiments slow vs fast growing fish and male vs female gonads. This appears to be the first of several Seriola genomes that will likely come out in 2017. The authors use commonly used software programs (Maker, Masurca, trinity etc) for analysis. However, authors should take care of how the data is presented, specifically in relation to gene model naming convention (see below).

Please add in references to all Supplementary files in main body of text. I was only able to find two references (supp 1 +4) in the main body of the text.
Please add a descriptive paragraph for each supplementary file that you provide.
Please add summary statistics on the genome annotation output from Maker. How many genes were identified, isoforms etc.
Line 396: Please use BUSCO instead of CEGMA for determining quality of this assembly. CEGMA is now outdated as stated by authors. Please also report the results of your quality check. How many genes are missing, fragmented etc?
Please include how close is the zona pellucida sperm-binding protein 4 gene you identify on Linkage group 12 to the Japanese marker that identifies the sex locus in S. quinqueradiata. It would be nice to see this explored more to build up more of a story to the article.

Please describe developmental differences observed in the 20 dph and 40dph small vs large fish. In RNA-seq experiments related to growth rate, it can be difficult to separate out differences in size due to genetics vs differences in developmental stage. In your experimental design it is possible that these two factors are confounded and it should be stated as such or not.

The biggest revision I have is that the authors are using the gene naming schema output from maker. A typical gene model schema for Seriola dumerili would be Sedum.G00000001 for Genes and Sedum.T00000001.1 Sedum.T00000001.2 etc for transcript isoforms of the genes. Renaming the gene models is important as other researchers will start using your gene model annotations and referring to them by name. Ideally, the authors will rename all the figures and tables with the new names. However, at a bare minimum the GFF file should be updated with new names and a 2 column reference file associating the maker names with the new naming schema supplied.

Please upload the renamed GFF file to GIGA Science.

Methods

Are the methods appropriate to the aims of the study, are they well described, and are necessary controls included? Yes

Conclusions

Are the conclusions adequately supported by the data shown? Yes

Reporting Standards

Does the manuscript adhere to the journal's guidelines on minimum standards of reporting? Yes Choose an item.

Statistics

Are you able to assess all statistics in the manuscript, including the appropriateness of statistical tests used? Yes, and I have assessed the statistics in my report.

Quality of Written English

Please indicate the quality of language in the manuscript: Acceptable

Declaration of Competing Interests

Please complete a declaration of competing interests, considering the following questions:

- Have you in the past five years received reimbursements, fees, funding, or salary from an organisation that may in any way gain or lose financially from the publication of this manuscript, either now or in the future?
- Do you hold any stocks or shares in an organisation that may in any way gain or lose financially from the publication of this manuscript, either now or in the future?
- Do you hold or are you currently applying for any patents relating to the content of the manuscript?
- Have you received reimbursements, fees, funding, or salary from an organization that holds or has applied for patents relating to the content of the manuscript?
- Do you have any other financial competing interests?
- Do you have any non-financial competing interests in relation to this paper?

If you can answer no to all of the above, write 'I declare that I have no competing interests' below. If your reply is yes to any, please give details below.

I am in the process of submitting the Seriola dorsalis genome assembly and annotation with emphasis on sex determination (also on linkage group 12). We are also writing a transcriptome paper on fast vs slow growing fish in Seriola dorsalis. While the data are similar, the species are different. I am very familiar with the Seriola genus and these kind of data analyses. I can provide an unbiased review.

I agree to the open peer review policy of the journal. I understand that my name will be included on my report to the authors and, if the manuscript is accepted for publication, my named report including any attachments I upload will be posted on the website along with the authors' responses. I agree for my report to be made available under an Open Access Creative Commons CC-BY license (http://creativecommons.org/licenses/by/4.0/). I understand that any comments which I do not wish to be included in my named report can be included as confidential comments to the editors, which will not be published.

I agree to the open peer review policy of the journal

To further support our reviewers, we have joined with Publons, where you can gain additional credit to further highlight your hard work (see: https://publons.com/journal/530/gigascience). On publication of this paper, your review will be automatically added to Publons, you can then choose whether or not to claim your Publons credit. I understand this statement.

Yes
