# Supplementary material for: Full genome survey and dynamics of gene expression in the greater amberjack Seriola dumerili
Source: Gigascience. 2017 Nov 8;6(12):1–13. doi: 10.1093/gigascience/gix108 (PMC5751066; doi:10.1093/gigascience/gix108)
Supplement: Reviewer-1-Report-(Revision-1).pdf [file gix108_reviewer-1-report-(revision-1).pdf]

Reviewer Report

Title: Full Genome Survey and Dynamics of Gene Expression in the Greater Amberjack Seriola dumerili

Version: Revision 1 **Date:** 9/15/2017

Reviewer name: Andrew Severin

Reviewer Comments to Author:

1) Thank you for updating your gene models.
Please update your supplementary tables to reflect the new gene models.

2) thank you for running BUSCO, In the future consider running an orthologous gene set such as vertebrata_odb9 that is closer to your species to get a more accurate assessment of genome assembly.

3) Please be sure to release the SRA SRP105319 after publication, i was not able to find it and is likely be held until publication.

4) You might consider taking out the two paragraphs (1 in results, 1 in discussion) about fast and slow growing fish and writing it up as a separate paper, it would tighten up the story and doesn't add a lot of value to this paper. (suggestion not a requirement)

Methods

Are the methods appropriate to the aims of the study, are they well described, and are necessary controls included? Yes

Conclusions

Are the conclusions adequately supported by the data shown? Yes

Reporting Standards

Does the manuscript adhere to the journal's guidelines on minimum standards of reporting? Yes Choose an item.

Statistics

Are you able to assess all statistics in the manuscript, including the appropriateness of statistical tests used? Yes, and I have assessed the statistics in my report.

Quality of Written English

Please indicate the quality of language in the manuscript: Acceptable

Declaration of Competing Interests

Please complete a declaration of competing interests, considering the following questions:

- Have you in the past five years received reimbursements, fees, funding, or salary from an organisation that may in any way gain or lose financially from the publication of this manuscript, either now or in the future?
- Do you hold any stocks or shares in an organisation that may in any way gain or lose financially from the publication of this manuscript, either now or in the future?
- Do you hold or are you currently applying for any patents relating to the content of the manuscript?
- Have you received reimbursements, fees, funding, or salary from an organization that holds or has applied for patents relating to the content of the manuscript?
- Do you have any other financial competing interests?
- Do you have any non-financial competing interests in relation to this paper?

If you can answer no to all of the above, write 'I declare that I have no competing interests' below. If your reply is yes to any, please give details below.

I am currently working on a Seriola dorsalis genome assembly and annotation. I have no competing interests related to Seriola Dumerili.

I agree to the open peer review policy of the journal. I understand that my name will be included on my report to the authors and, if the manuscript is accepted for publication, my named report including any attachments I upload will be posted on the website along with the authors' responses. I agree for my report to be made available under an Open Access Creative Commons CC-BY license (http://creativecommons.org/licenses/by/4.0/). I understand that any comments which I do not wish to be included in my named report can be included as confidential comments to the editors, which will not be published.

I agree to the open peer review policy of the journal

To further support our reviewers, we have joined with Publons, where you can gain additional credit to further highlight your hard work (see: https://publons.com/journal/530/gigascience). On publication of this paper, your review will be automatically added to Publons, you can then choose whether or not to claim your Publons credit. I understand this statement.

Yes
