# Supplementary material for: Full genome survey and dynamics of gene expression in the greater amberjack Seriola dumerili
Source: Gigascience. 2017 Nov 8;6(12):1–13. doi: 10.1093/gigascience/gix108 (PMC5751066; doi:10.1093/gigascience/gix108)
Supplement: Reviewer-2-Report-(Original-Submission).pdf [file gix108_reviewer-2-report-(original-submission).pdf]

Reviewer Report

Title: Full Genome Survey and Dynamics of Gene Expression in the Greater Amberjack Seriola dumerili

Version: Original Submission **Date:** 7/10/2017

Reviewer name: Benjamin Reading

Reviewer Comments to Author:

Full Genome Survey and Dynamics of Gene Expression in the Greater Amberjack Seriola dumerili

General Comments:

Lines 147-149: "Out of the 53,023 obtained transcripts 44,371 were successfully mapped to the generated in silico groups of the greater amberjack and 30,342 transcripts to the genome of medaka." Do the authors have any indication as to why more than 20,000 predicted gene transcripts were identified and mapped to the generated in silico groups compared to the medaka genome? This (i.e., 53,000 gene transcripts) is substantially more than would be predicted for a typical diploid teleost genome (about 30,000). Splice variation or other reasons such as gene duplications?

Line 232: What do the authors mean by "sex-linked chromosomes"? Genes/loci that are found on sex chromosomes are "sex-linked", however the chromosomes themselves are considered "sex chromosomes" or "sex determining chromosomes". Please clarify.

Lines 349-350: "Gonad samples of four female and four male amberjacks (4n) were received from fish maintained at an aquaculture facility in Salamina." What were the sizes and maturity status of the fish? (i.e., sexually mature, and reproductive state, etc.). The "reproductive season (May)" is indicated, but were the males spermiating and females in post-vitellogenesis/ovarian maturation/ovulated?

Lines 252-253: "Thus, the present finding may indicate the importance of ubiquitination during sex determination." The role of the ubiquitin path also is related to control of the cell cycle (expression and degradation of regulatory proteins such as cyclins), including meiosis and germ cell proliferation and differentiation for gametogenesis. Especially when considering Figure 2, where "Histone Deubiquitination", "Protein Ubiquitination", and "Ubiquitin-dependent protein catabolic process" are considered enriched processes on putative sex chromosomes. Were any such gene transcripts identified in the gonad transcriptomes? See below references and citations within:

Chapman, R.W., Reading, B.J., and Sullivan, C.V. 2014. Ovary transcriptome profiling via artificial intelligence reveals a transcriptomic fingerprint predicting egg quality in striped bass, Morone saxatilis. PLoS ONE 9(5):e96818.

Reading, B.J., Chapman, R.W., Schaff, J.E., Scholl, E.H., Opperman, C.H., and Sullivan, C.V. 2012. An ovary

transcriptome for all maturational stages of the striped bass (Morone saxatilis), a highly advanced perciform fish. BMC Research Notes 5:111 (doi: 10.1186/1756-0500-5-111).

Also, "evidence points to the important role of ubiquitination in the regulation of spermatogenesis from the very beginning up to the spermatid differentiation". The same applies to oogenesis (from primary growth through ovulation and also directing early embryonic development; see citations above).

Line 345-346: "EDTA containing tubes". What concentration (mM) of EDTA was used as an anticoagulant?

Specific Comments:

Line 88: Plural/singular agreement. "sex determining mechanism" should be "sex determining mechanisms" or "sex determination mechanisms".

Line 92: Plural/singular agreement. "highly targeted research field" should be "highly targeted research fields".

Line 95: The word "been" can be omitted from the sentence "has been proved to be" ("has proved to be").

Line 138: "NCBI nr" the authors may consider stating the definition of "nr" as "NCBI non-redundant".

Line 165: The authors may consider changing "as differential expressed resulting" to "to be differentially expressed, resulting".

Line 166: The authors may consider changing "illustrated in form of a" to "illustrated in the form of a".

Line 167: Plural/singular agreement. "gonads gene expression" should be "gonad gene expression".

Line 221: The authors may consider revising the sentence "determined the number of chromosomes in the greater amberjack to 24" to "determined the haploid number of chromosomes in the greater amberjack to be 24".

Lines 230-231: The sentence "The homologous to the greater amberjack in silico group 12 in medaka were found to be Chr.8 and in three-spine stickleback Chr.V and XI" is awkward. Do the authors mean to state "The greater amberjack in silico group 12 was found to be homologous to medaka Chr.8 and three-spine stickleback Chr.V and XI"?

Line 246: "Enrichment analysis of transcripts successfully in the present study" is unclear ("successfully" what?).

Line 295: "hypothesis that RA signaling pathway" should be "hypothesis that the RA signaling pathway".

Line 306: "their processes" should be "the processes".

Line 344: Should "HCMR" be spelled out at first use?

Lines 346-349: The authors might consider defining "n" here as "fish" in relation to biological sample size (e.g., n = 4 fish).

Table 4: Approved gene names should be in italic font.

Methods

Are the methods appropriate to the aims of the study, are they well described, and are necessary controls included? Yes

Conclusions

Are the conclusions adequately supported by the data shown? Yes

Reporting Standards

Does the manuscript adhere to the journal's guidelines on minimum standards of reporting? Yes Choose an item.

Statistics

Are you able to assess all statistics in the manuscript, including the appropriateness of statistical tests used? Yes, and I have assessed the statistics in my report.

Quality of Written English

Please indicate the quality of language in the manuscript: Needs some language corrections before being published

Declaration of Competing Interests

Please complete a declaration of competing interests, considering the following questions:

- Have you in the past five years received reimbursements, fees, funding, or salary from an organisation that may in any way gain or lose financially from the publication of this manuscript, either now or in the future?
- Do you hold any stocks or shares in an organisation that may in any way gain or lose financially from the publication of this manuscript, either now or in the future?

- Do you hold or are you currently applying for any patents relating to the content of the manuscript?
- Have you received reimbursements, fees, funding, or salary from an organization that holds or has applied for patents relating to the content of the manuscript?
- Do you have any other financial competing interests?
- Do you have any non-financial competing interests in relation to this paper?

If you can answer no to all of the above, write 'I declare that I have no competing interests' below. If your reply is yes to any, please give details below.

I declare that I have no competing interests.

I agree to the open peer review policy of the journal. I understand that my name will be included on my report to the authors and, if the manuscript is accepted for publication, my named report including any attachments I upload will be posted on the website along with the authors' responses. I agree for my report to be made available under an Open Access Creative Commons CC-BY license (http://creativecommons.org/licenses/by/4.0/). I understand that any comments which I do not wish to be included in my named report can be included as confidential comments to the editors, which will not be published.

I agree to the open peer review policy of the journal

To further support our reviewers, we have joined with Publons, where you can gain additional credit to further highlight your hard work (see: https://publons.com/journal/530/gigascience). On publication of this paper, your review will be automatically added to Publons, you can then choose whether or not to claim your Publons credit. I understand this statement.

Yes
