# Supplementary material for: Full genome survey and dynamics of gene expression in the greater amberjack Seriola dumerili
Source: Gigascience. 2017 Nov 8;6(12):1–13. doi: 10.1093/gigascience/gix108 (PMC5751066; doi:10.1093/gigascience/gix108)
Supplement: Reviewer-3-Report-(Original-Submission).pdf [file gix108_reviewer-3-report-(original-submission).pdf]

Reviewer Report

Title: Full Genome Survey and Dynamics of Gene Expression in the Greater Amberjack Seriola dumerili

Version: Original Submission **Date:** 7/12/2017

Reviewer name: Christiaan Henkel

Reviewer Comments to Author:

This manuscript describes a genome assembly and annotation for the greater amberjack, a species for which almost no genomic resources are available. As the assembly itself is based on Illumina technology only, it is highly fragmented. Therefore, the authors have complemented it with a genetic linkage map from a closely related species. The assembly is used to support the analysis of two RNA-seq studies, one aimed at understanding the sex determination mechanism, and another on muscle growth characteristics, both topic highly relevant to aquaculture. As these analyses yield neither clear sex-determination or growth markers, nor mechanistic insight, they are best interpreted as data resources for future in-depth studies.

There are several issues I would ask the authors to address:

1. The full assembly process is somewhat unconventional, and the manuscript could be improved with additional details and clarifications. Specifically:
1a. The 'in silico groups' (first introduced in the abstract, l. 58, and then in Results l. 145) are hypothetical linkage groups/pseudochromosomes. It would be helpful if they are immediately introduced as such.
1b. Why did you normalize the genomic reads (l. 133 and l. 394) using a transcriptome assembler? Is this standard practice for the MaSuRCA assembler? Doesn't it introduce artifacts?
1c. It is not immediately clear that the scaffolds and contigs in l. 135 are two different representations of the same assembly, i.e. there is no single dataset consisting of the combined 45909 scaffolds and 62353 contigs.
1d. The genome completeness analysis results (l. 396) should be included in the Results, and preferably performed using BUSCO instead of/in addition to CEGMA.
1e. According to l. 143, 14990 scaffolds were mapped to the medaka genome to construct the 'in silico groups'. The number suggests this is only a small fraction of the genome, but I assume these are primarily larger scaffolds. A more informative number would therefore be the percentage of the assembly that could be assigned to a group. (I believe it is ~80%, based on a sequence file provided for review but not listed in the manuscript).
1f. The Results suggest 'synteny analysis' (l. 145) is possible with the hypothetical groups. This is not really performed, except for figures 1b-c. In the end, the 'on-to-one relationship' between Seriola and medaka (Discussion l. 225-227) has some aspects of circular reasoning, as it is based on only 468 marker matches. Therefore, this alignment is mostly based on an indirect alignment of the medaka genome with itself. (Incidentally - the manuscript in different places refers to 'circus plots', which should be 'Circos

plots'.)

2. The gonadal transcriptome analysis is based on a (technically sound) RNA-seq comparison of testis with ovaries. However, I think the interpretation could be improved:
2a. These are completely different tissues. Therefore, 'upregulation' and 'downregulation' are not really suitable descriptions of gene expression level differences, as there is no developmental path leading from one condition to the other by tweaking expression levels. Therefore, 'higher/lower expression in' would be more appropriate.
2b. Related: when comparing different tissues, these differences are best interpreted qualitatively rather than quantitatively (and without statistical tests), as there is no reasonable way to align the profiles. In this study, standard DESeq2 normalization (l. 444) is presumably applied, but using different tissues almost certainly violates the assumptions behind its method (i.e. minimize gene expression differences). Its validity needs to be demonstrated for this data, for example by showing the overall similarity between the profiles and their successful normalization.
2c. In general, this study examines sex-specific gene expression, but also discusses sex determination. Is there a reason to assume the genes highly 'differentially expressed' in gonads are the genes responsible for sex determination?
2d. Related: the genome assembly is based on two individuals, a male and a female. Unfortunately, the sequencing libraries have been pooled (l. 377), otherwise it would have been relatively straightforward to identify (small) loci not present in males (assuming ZW sex determination). It may still be possible to identify contigs with relatively low coverage - these could be candidate sex determination regions.
2e. What is the difference between the differential expression analyses of lines 164-165 and lines 170-171?

3. Line 181: please mention that these transcriptomes are of muscle tissue (the current phrasing suggests complete animals have been profiled). For this part of the study, animals at the same age were used. I assume the slow- and fast-growing animals were at the same developmental stage here?

4. The Discussion of the genome assembly quality in the light of other Mediterranean fish species is not entirely appropriate (lines 195-219). The species are not closely related. Although genome size and sequence coverage are factors influencing short-read assembly contiguity, genome structure (i.e. repeat landscape) and heterozygosity level are probably much more important. Both have not been analyzed. The Seriola assembly may well have ended up more fragmented than necessary by pooling the genomes of two individuals. This choice should be discussed, and an analysis of overall heterozygosity (e.g. using a k-mer profile) would be relevant here.

Methods

Are the methods appropriate to the aims of the study, are they well described, and are necessary controls included? Yes

Conclusions

Are the conclusions adequately supported by the data shown? Yes

Reporting Standards

Does the manuscript adhere to the journal's guidelines on minimum standards of reporting? Yes Choose an item.

Statistics

Are you able to assess all statistics in the manuscript, including the appropriateness of statistical tests used? Yes, and I have assessed the statistics in my report.

Quality of Written English

Please indicate the quality of language in the manuscript: Acceptable

Declaration of Competing Interests

Please complete a declaration of competing interests, considering the following questions:

- Have you in the past five years received reimbursements, fees, funding, or salary from an organisation that may in any way gain or lose financially from the publication of this manuscript, either now or in the future?
- Do you hold any stocks or shares in an organisation that may in any way gain or lose financially from the publication of this manuscript, either now or in the future?
- Do you hold or are you currently applying for any patents relating to the content of the manuscript?
- Have you received reimbursements, fees, funding, or salary from an organization that holds or has applied for patents relating to the content of the manuscript?
- Do you have any other financial competing interests?
- Do you have any non-financial competing interests in relation to this paper?

If you can answer no to all of the above, write 'I declare that I have no competing interests' below. If your reply is yes to any, please give details below.

I declare that I have no competing interests

I agree to the open peer review policy of the journal. I understand that my name will be included on my report to the authors and, if the manuscript is accepted for publication, my named report including any attachments I upload will be posted on the website along with the authors' responses. I agree for my report to be made available under an Open Access Creative Commons CC-BY license (http://creativecommons.org/licenses/by/4.0/). I understand that any comments which I do not wish to be included in my named report can be included as confidential comments to the editors, which will not be published.

I agree to the open peer review policy of the journal

To further support our reviewers, we have joined with Publons, where you can gain additional credit to further highlight your hard work (see: https://publons.com/journal/530/gigascience). On publication of this paper, your review will be automatically added to Publons, you can then choose whether or not to claim your Publons credit. I understand this statement.

Yes
