# Supplementary material for: Full genome survey and dynamics of gene expression in the greater amberjack Seriola dumerili
Source: Gigascience. 2017 Nov 8;6(12):1–13. doi: 10.1093/gigascience/gix108 (PMC5751066; doi:10.1093/gigascience/gix108)
Supplement: Reviewer-3-Report-(Revision-1).pdf [file gix108_reviewer-3-report-(revision-1).pdf]

Reviewer Report

Title: Full Genome Survey and Dynamics of Gene Expression in the Greater Amberjack Seriola dumerili

Version: Revision 1 **Date:** 9/15/2017

Reviewer name: Christiaan Henkel

Reviewer Comments to Author:

I thank the authors for the answers they provided, and I am satisfied with most of the changes to the manuscript. For three issues, I would still like to suggest some minor clarification and/or context.

Please also revise the figure order/labels, fig 2 of the text is included as fig 8.

Suggestion 1 (previously 1f): The Seriola transcripts are mapped to the medaka genome in order to select genes putatively located at the sex-determining locus on chromosome 12. This is a technical sorting procedure, which is perfectly suitable. Figure 8c (2c) then illustrates the quality of this sorting procedure, as does figure 2a (3a). The use of the word 'synteny' in the text (l 154) and the use of Circos plots could suggest these are analyses of genome evolution, which is not studied. Perhaps 'synteny' could be avoided here?

Suggestion 2 (previously 2b): I am aware of the ubiquitous use of DESeq2 and tools like it. That they are widely used does not mean that they are always used appropriately. I was getting at the issue of between-sample normalization, which is mostly automatic in these tools and assumes samples are comparable. In this case, I doubt the samples are comparable under the implicit assumptions of DESeq normalization. The illustrations in figure 3 are post-analysis, and do not address this concern. If anything, the heatmap and the skew towards higher expression in one tissue (line 178) imply that the samples are not well comparable. This issue is widespread in RNA-seq analysis, and I do not expect this paper to address it at a fundamental level; however, I would suggest caution in interpreting these differential expression results, with perhaps a caveat in the Results, and including more details on DESeq2 deployment in the Methods.

Suggestion 3 (previously 2c): I concede that the assumption I made about the link between sex determination and sex-linked gene expression is not emphasized in the manuscript. My apologies for this. I do, however, think it is a natural question to ask, which other readers may do as well. Both issues are discussed immediately after each other, with the sentences linking them (l246-250) discussing a gene (GIPC1) that might be sex-determining, but is not differentially expressed, thus encouraging my interpretation of an implicit assumption. Perhaps you could clarify the difference between the two analyses for the readers, for instance by mentioning there that such a link between sex determination and sex-linked expression is neither necessary nor expected.

Details:

- l145 conversed - > conserved
- fig 3: one species is not labeled, I assume this is Tetraodon (based on the Methods)?
- The k-mer analyses are useful. There is an additional bump (modeled as errors) before the heterozygous peak. Could this be the result of mixing genomic information from two individuals?

Methods

Are the methods appropriate to the aims of the study, are they well described, and are necessary controls included? Yes

Conclusions

Are the conclusions adequately supported by the data shown? Yes

Reporting Standards

Does the manuscript adhere to the journal's guidelines on minimum standards of reporting? Yes Choose an item.

Statistics

Are you able to assess all statistics in the manuscript, including the appropriateness of statistical tests used? Yes, and I have assessed the statistics in my report.

Quality of Written English

Please indicate the quality of language in the manuscript: Acceptable

Declaration of Competing Interests

Please complete a declaration of competing interests, considering the following questions:

- Have you in the past five years received reimbursements, fees, funding, or salary from an organisation that may in any way gain or lose financially from the publication of this manuscript, either now or in the future?
- Do you hold any stocks or shares in an organisation that may in any way gain or lose financially from the publication of this manuscript, either now or in the future?
- Do you hold or are you currently applying for any patents relating to the content of the manuscript?
- Have you received reimbursements, fees, funding, or salary from an organization that holds or has applied for patents relating to the content of the manuscript?
- Do you have any other financial competing interests?
- Do you have any non-financial competing interests in relation to this paper?

If you can answer no to all of the above, write 'I declare that I have no competing interests' below. If your reply is yes to any, please give details below.

I declare that I have no competing interests

I agree to the open peer review policy of the journal. I understand that my name will be included on my report to the authors and, if the manuscript is accepted for publication, my named report including any attachments I upload will be posted on the website along with the authors' responses. I agree for my report to be made available under an Open Access Creative Commons CC-BY license (http://creativecommons.org/licenses/by/4.0/). I understand that any comments which I do not wish to be included in my named report can be included as confidential comments to the editors, which will not be published.

I agree to the open peer review policy of the journal

To further support our reviewers, we have joined with Publons, where you can gain additional credit to further highlight your hard work (see: https://publons.com/journal/530/gigascience). On publication of this paper, your review will be automatically added to Publons, you can then choose whether or not to claim your Publons credit. I understand this statement.

Yes
